# Effects of traditional Chinese mind–body exercises on depressive symptoms in middle-aged and older adults: a multilevel meta-analysis with exploratory dose–response and machine learning analyses

**DOI:** 10.3389/fpsyg.2026.1887582

**Published:** 2026-07-13

**Authors:** Xuesong Yang, Jianwei Guo, Bingquan Luo

**Affiliations:** 1 School of Wushu and Performance, Capital University of Physical Education and Sports, Beijing, China; 2 Shanghai University of Sport, Shanghai, China; 3 School of Sport Management and Communication, Capital University of Physical Education and Sports, Beijing, China

**Keywords:** traditional Chinese mind–body exercises, tai chi, qigong, middle-aged adults, depressive symptoms, meta-analysis, dose–response analysis, machine learning

## Abstract

**Objective:**

This study evaluated the effects of traditional Chinese mind–body exercises on depressive symptoms in middle-aged and older adults and examined potential dose-related and study-level moderators.

**Methods:**

PubMed, Web of Science, Embase, Cochrane Library, CNKI, VIP, and Wanfang were searched through May 3, 2026, for randomized controlled trials. A three-level random-effects model was used to account for dependent effect sizes. Subgroup analyses, meta-regression, exploratory dose–response analyses, and an XGBoost-based machine-learning extension were conducted. The risk of bias and the certainty of evidence were assessed using RoB 2 and GRADE.

**Results:**

Thirty randomized controlled trials were included. Traditional Chinese mind–body exercises were associated with a statistically significant reduction in depressive symptoms among middle-aged and older adults (SMD = −0.88; 95% CI, −1.18 to −0.58; *p* < 0.001). Sensitivity and supplementary analyses generally supported the robustness of the main findings; however, substantial heterogeneity and risk of bias contributed to low certainty of the evidence. Subgroup analyses indicated that most estimates favored traditional Chinese mind–body exercises, with statistically significant subgroup differences observed only by country. Meta-regression showed that older age was associated with a smaller intervention effect (*β* = 0.03, *p* = 0.025). Exploratory dose–response analyses did not identify a statistically supported optimal dose, although descriptive patterns were observed. XGBoost and SHAP analyses suggested that age, scale type, total dose, and session duration may be important study-level features, though these findings were exploratory.

**Conclusion:**

Traditional Chinese mind–body exercises may reduce depressive symptoms in middle-aged and older adults; however, the certainty of the evidence is low because of substantial heterogeneity and risk of bias. Dose–response and machine-learning findings should be considered exploratory. Further high-quality randomized controlled trials are warranted to confirm these findings and refine intervention parameters.

**Systematic review registration:**

https://www.crd.york.ac.uk/PROSPERO/view/CRD420261388878, identifier PROSPERO (CRD420261388878).

## 1 Introduction

Depression is a major public health concern among middle-aged and older adults, and its public health relevance is increasing as populations age worldwide. The number of adults aged 60 years and older has exceeded 1 billion and is expected to reach 1.4 billion by 2030 (Hu et al., 2022). A recent systematic review reported a pooled prevalence of depression of 28.4% in older adults and suggested that differences in geographic region, screening tools, sample representativeness, and study quality may partly explain the observed heterogeneity (Hu et al., 2022). Depression in this age group is also associated with emotional distress, higher healthcare costs, increased suicide risk, and greater mortality (Aziz and Steffens, 2013; Jiang et al., 2020), underscoring the need for effective and accessible prevention and management strategies.

The burden of depression in middle-aged and older adults is shaped by multiple biological and psychosocial factors. Biologically, age-related changes in neuroendocrine function, including dysregulation of the hypothalamic–pituitary–adrenal axis, chronic inflammation, and reduced neuroplasticity, may affect emotional regulation (Stetler and Miller, 2011; Read et al., 2017; Alexopoulos, 2019; Manandhar et al., 2019; Szymkowicz et al., 2023). Psychosocial factors are also important. Reduced social support, loneliness, and adjustment difficulties after retirement may lower self-esteem and increase vulnerability to depression (Laks and Engelhardt, 2010; Donovan and Blazer, 2020). Moreover, symptoms such as insomnia, fatigue, and changes in appetite may be perceived by some older adults as part of normal aging, which can delay recognition and treatment (Sadavoy, 2009; Wu et al., 2012).

Medication remains an important treatment option for depression. However, long-term antidepressant use may be accompanied by adverse effects, including dizziness, fatigue, digestive symptoms, and increased risk of falls (Van Poelgeest et al., 2021; Niarchou et al., 2024). Polypharmacy and poor adherence may also reduce long-term effectiveness in older adults (Stahl et al., 2023; Niarchou et al., 2024). Therefore, safe, accessible, and sustainable nonpharmacological approaches are needed. In recent years, traditional Chinese mind–body exercises have received increasing attention as potential interventions for mental health. These exercises combine physical movement, breath regulation, and mental focus, and include practices such as Tai Chi, Qigong, Baduanjin, Yijinjing, Liuzijue, and Daoyin (Zou et al., 2018b; Lin et al., 2022; Qi et al., 2022; Wang et al., 2025). Although these modalities differ in movement form and training routines, they share several core features, including slow coordinated movement, breathing regulation, attentional focus, and body awareness (Wayne and Kaptchuk, 2008; Larkey et al., 2009; Zou et al., 2018b). Therefore, they can be considered as a broad category of traditional Chinese mind–body exercises, while potential differences between modalities can be examined through subgroup and moderator analyses. Previous studies suggest that these exercises may improve depressive symptoms through pathways related to neuroendocrine regulation, sleep quality, inflammation, and psychological well-being (Chan et al., 2020; Chen et al., 2024, 2025; Qiu et al., 2024; Feng et al., 2025).

Although traditional Chinese mind–body exercise and related multicomponent interventions may alleviate depressive symptoms, existing studies vary widely in participant characteristics, intervention modalities, comparator conditions, and outcome measures. These differences may contribute to inconsistent findings and make it difficult to determine whether intervention effects differ across populations, intervention types, and dose-related characteristics. In addition, previous syntheses have not fully addressed dependence among multiple effect sizes from the same study or potential nonlinear and interactive patterns among study-level features. Accordingly, this study primarily aimed to examine whether traditional Chinese mind–body exercises and related multicomponent interventions were associated with greater reductions in depressive symptoms than control conditions among middle-aged and older adults. To address this aim, we used a multilevel meta-analytic approach to synthesize the available evidence while accounting for dependent effect sizes. Conventional subgroup and meta-regression analyses were used to examine potential moderators, while exploratory dose–response analyses were used to investigate possible dose-related patterns. Given the limited number of effect sizes, the XGBoost-based machine learning extension was not intended for confirmatory prediction or causal inference but was used as a supplementary hypothesis-generating approach to explore the relative importance of study-level features and potential nonlinear or interactive patterns. These analyses were interpreted alongside the primary multilevel meta-analysis to provide a more comprehensive assessment of the available evidence.

## 2 Materials and methods

### 2.1 Study design

This systematic review and meta-analysis were conducted in accordance with the PRISMA guidelines (Moher et al., 2015). Before initiating the literature screening, the review protocol was registered with the PROSPERO (registration ID: CRD420261388878; available at: https://www.crd.york.ac.uk/PROSPERO/view/CRD420261388878), and the review closely followed the PRISMA framework.

### 2.2 Study inclusion criteria

Studies were considered eligible if they met the following criteria: (1) they used an RCT design to assess the effects of traditional Chinese mind–body exercises or related multicomponent interventions on depressive symptoms in middle-aged and older adults; (2) the intervention included traditional Chinese mind–body exercises, either as a standalone intervention, as an adjunct to usual care, or as part of a multicomponent intervention, while the comparator did not include the same traditional Chinese mind–body exercises component; (3) participants were adults aged 45 years or older, with those aged 45–59 years categorized as middle-aged and those aged 60 years or older categorized as older adults; (4) depressive symptoms were assessed after the intervention using validated depression scales. Because the target outcome of this review was depressive symptoms rather than a clinically diagnosed depressive disorder, a formal diagnosis of depression was not required for eligibility. When reported, information on clinical diagnosis or baseline depressive status was extracted to describe study characteristics and interpret heterogeneity; and (5) studies were published in English or Chinese with full texts available.

Studies were excluded if they were non-original or non-experimental articles, including theoretical papers, case reports, animal or cell studies, reviews, meta-analyses, duplicate publications, conference abstracts, unpublished or non-peer-reviewed grey literature, and informal single-session counseling studies. Grey literature was excluded because its methodological details, outcome data, and risk-of-bias information are often insufficiently reported, potentially limiting consistent data extraction and quality assessment. Reference lists of relevant reviews and meta-analyses were manually screened to identify additional eligible primary studies.

### 2.3 Search strategy

A comprehensive search was conducted across several databases, including PubMed, Web of Science, Embase, Cochrane Library, Chinese National Knowledge Infrastructure (CNKI), Chongqing VIP Database (VIP), and Wanfang Data Knowledge Service Platform (Wanfang), from inception to May 3, 2026. The objective was to identify all RCTs assessing the impact of traditional Chinese mind–body exercises, such as Tai Chi, Baduanjin, Qigong, standing meditation, and Wuqinxi, on depressive symptoms among middle-aged and older adults.

The search approach was formulated using the PICOS framework: the target population (P) included adults aged 45 and older; the intervention (I) encompassed any traditional Chinese mind–body exercises, with a minimum of one session; the comparator (C) involved wait-list control, standard care, or conventional treatment; the primary outcome (O) focused on the standardized depression scale score after the intervention; and the study design (S) was an RCT. A comprehensive search strategy was employed, integrating free-text keywords and database-specific subject headings, including MeSH terms, related to “traditional Chinese mind–body exercises,” “middle-aged and older adults,” and “depressive symptoms.” Detailed search strategies for each database are provided in Table S1 of the Supplementary materials. During screening, only studies published in English or Chinese were considered eligible, in accordance with the predefined inclusion criteria.

### 2.4 Study selection process

The study selection process followed the PRISMA guidelines. All records were imported into Zotero 7.0 to remove duplicates. Two reviewers independently screened titles and abstracts, and screening decisions were coded as inclusion (1) or exclusion (0). Inter-rater agreement was assessed using Cohen’s *κ* (Cohen, 1960). Potentially eligible studies were then assessed in full text according to the PICOS criteria. Disagreements during title-and-abstract screening and full-text assessment were resolved through discussion or consultation with a third reviewer. Data were independently extracted by two reviewers using a standardized form and were cross-checked before analysis. Because the extracted information included continuous variables, categorical variables, and descriptive fields, a single agreement coefficient was not calculated for data extraction. All extraction discrepancies were reviewed item by item and resolved by consensus before the final dataset was used for analysis.

### 2.5 Data synthesis

All statistical analyses were conducted in R version 4.3.3 using the packages meta, metafor, dmetar, dosresmeta, clubSandwich, and ggplot2. The package names were checked against the analysis code and corrected if necessary. When studies reported age separately by group, overall means and pooled standard deviations were calculated using sample-size-weighted formulas for subsequent meta-regression analyses. The overall mean was determined as follows: $\mu =\frac{n_{1}\bar{x_{1}}+n_{2}\bar{x_{2}}}{n_{1}+n_{2}}$ . The pooled standard deviation was derived as: $s_{p}=\sqrt{\frac{(n_{1}-1)s_{1}^{2}+(n_{2}-1)s_{2}^{2}}{n_{1}+n_{2}-2}}$ . For continuous outcomes, post-intervention depression scores were used to calculate between-group effect sizes because change-score standard deviations were inconsistently reported across studies. Given the randomized design of the included trials, post-intervention between-group comparisons were considered appropriate, though potential baseline imbalance was taken into account when interpreting the results. Because different depression scales were used across studies, effect sizes were calculated as Hedges’ g standardized mean differences (SMDs). Negative SMDs indicated greater reductions in depressive symptoms in the intervention group.

The primary synthesis used a three-level random-effects model with the rma.mv function in the metafor package (Assink and Wibbelink, 2016). Effect sizes were nested within studies using the random-effects structure random = ~1|study_id/effect_id. Multiple eligible effect sizes from the same study were retained when studies reported more than one depression scale, intervention group, or eligible comparison to avoid selective outcome reporting. The dependence among these effect sizes was addressed by a three-level model. Model parameters were estimated using restricted maximum likelihood (REML), and heterogeneity was assessed using the Q statistic, variance components, and multilevel I^2^ estimates. Funnel plots, Egger’s regression test, and trim-and-fill analysis were used to assess possible small-study effects or publication bias (Egger et al., 1997). Influence diagnostics were based on standardized residuals and Cook’s distance (Viechtbauer and Cheung, 2010). A study was considered influential if it had a standardized residual with |z| > 2.5 and a Cook’s distance greater than three times the mean Cook’s distance. When an influential study had a disproportionate impact on the pooled estimate, an influence-adjusted model was also fitted, and the full-data model was retained and reported. Leave-one-out analysis was used to assess the stability of the pooled effect.

Pre-specified subgroup analyses and REML-based meta-regression were conducted to assess potential sources of heterogeneity (Van Houwelingen et al., 2002). Additional analyses were conducted to address complementary questions: cumulative meta-analysis examined the accumulation of evidence over time (Lau et al., 1992); p-curve analysis assessed evidential value among statistically significant findings (Simonsohn et al., 2014); and trial sequential analysis evaluated whether the accumulated information size was sufficient under specified assumptions (Wetterslev et al., 2008). TSA used a two-sided *α* of 0.05 and *β* of 0.20, with the required information size estimated within a random-effects framework based on the observed effect size and heterogeneity. CR2 cluster-robust variance estimation was used as a small-sample robustness check. Dose–response analyses were conducted as exploratory extensions using nonlinear and Bayesian approaches (Crippa and Orsini, 2016; Hamza et al., 2021). XGBoost and SHAP analyses were used solely to explore the relative contributions of study-level features to predicted SMD values and were not interpreted as confirmatory predictions or causal inference (Crippa and Orsini, 2016; Hamza et al., 2021; Ponce-Bobadilla et al., 2024). Because these supplementary analyses were based on aggregate study-level data and a small number of effect sizes, their findings were considered exploratory and hypothesis-generating.

### 2.6 Risk of Bias and certainty of evidence assessment

The evaluation of bias was conducted using the Cochrane RoB 2 tool (2019 edition), which encompassed five key areas: (1) randomization process; (2) deviations from planned interventions; (3) missing outcome data; (4) outcome measurement; and (5) selection of reported results (Sterne et al., 2019). Two reviewers independently evaluated each area and categorized the risk of bias as “low risk,” “some concerns,” or “high risk.” Disagreements were resolved through discussion or by a third reviewer. Cohen’s *κ* was calculated before consensus, and the final RoB 2 judgments used in the synthesis were based on consensus ratings. The overall confidence in the evidence regarding the primary outcome (scores on the depressive symptom scale) and all subgroup analyses was evaluated using the GRADE framework and established downgrading criteria. The results were compiled into tables created with the online GRADEpro GDT tool (Guyatt et al., 2008).

## 3 Results

### 3.1 Study selection

**Figure 1:**
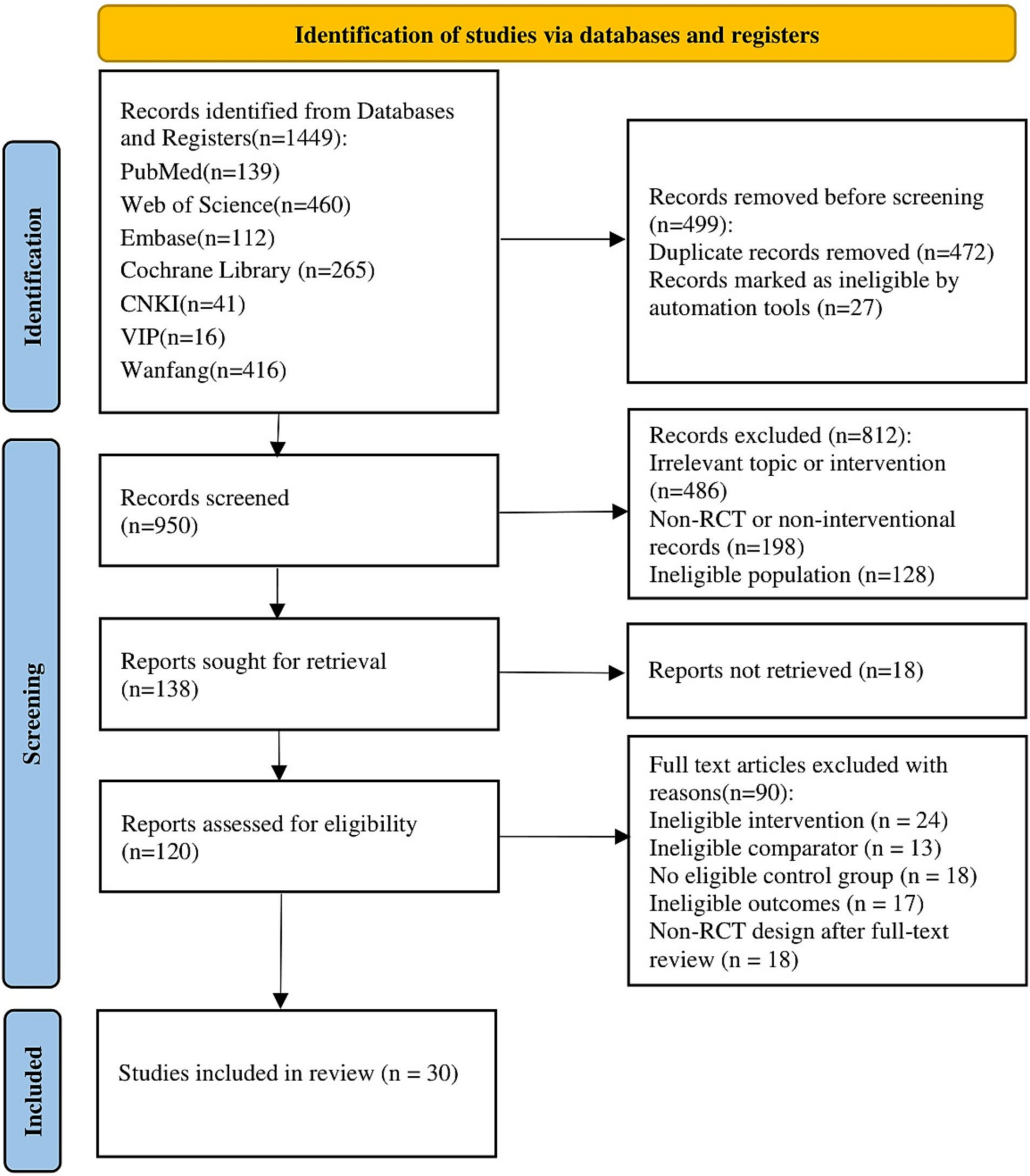
Flow diagram of the selection process.

From seven databases, a total of 1,449 records were identified (Figure 1). Before formal screening, Zotero was used to remove 472 duplicate entries, and ASReview LAB was used to identify and exclude 27 records that were clearly ineligible. This left 950 records for the title-and-abstract screening phase. Agreement between raters during this phase was high (Cohen’s *κ* = 0.87), indicating strong reliability. Following this, 812 records that failed to meet the inclusion criteria were discarded, and efforts were made to retrieve 138 reports. Of these, 18 could not be obtained, leaving 120 reports available for full-text eligibility evaluation. The inter-rater agreement at this stage was similarly high (Cohen’s κ = 0.87). Ultimately, 90 full-text articles were excluded for the reasons detailed in Figure 1, leaving 30 randomized controlled trials for inclusion in the systematic review. For data extraction, all items were independently checked by two reviewers, and discrepancies were resolved by consensus before analysis (Detailed calculations of screening agreement are provided in Supplementary Text S1).

### 3.2 Risk of Bias of included studies

**Figure 2:**
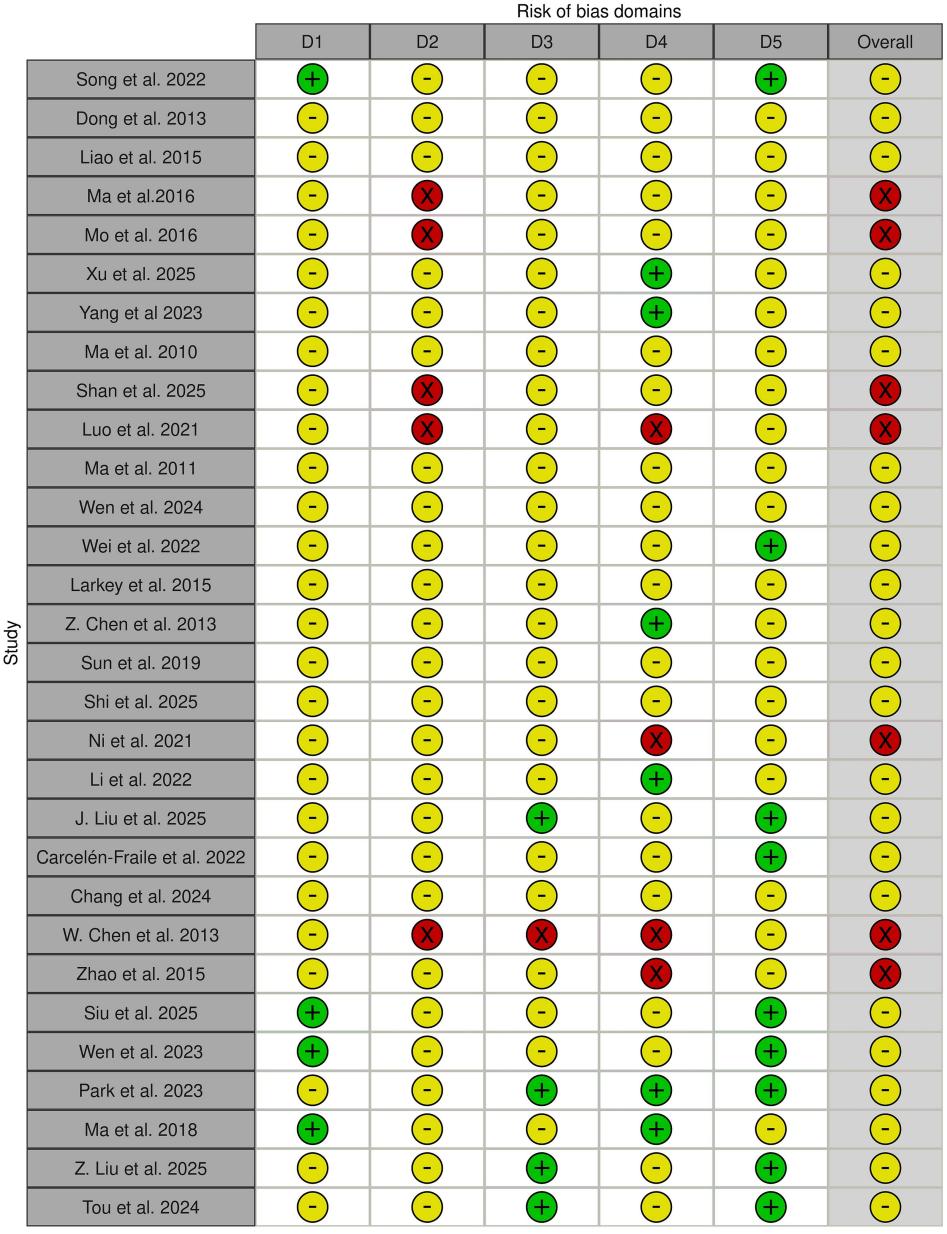
Risk of bias summary. Low risk, green; some concerns, yellow; high risk, red.

**Figure 3:**
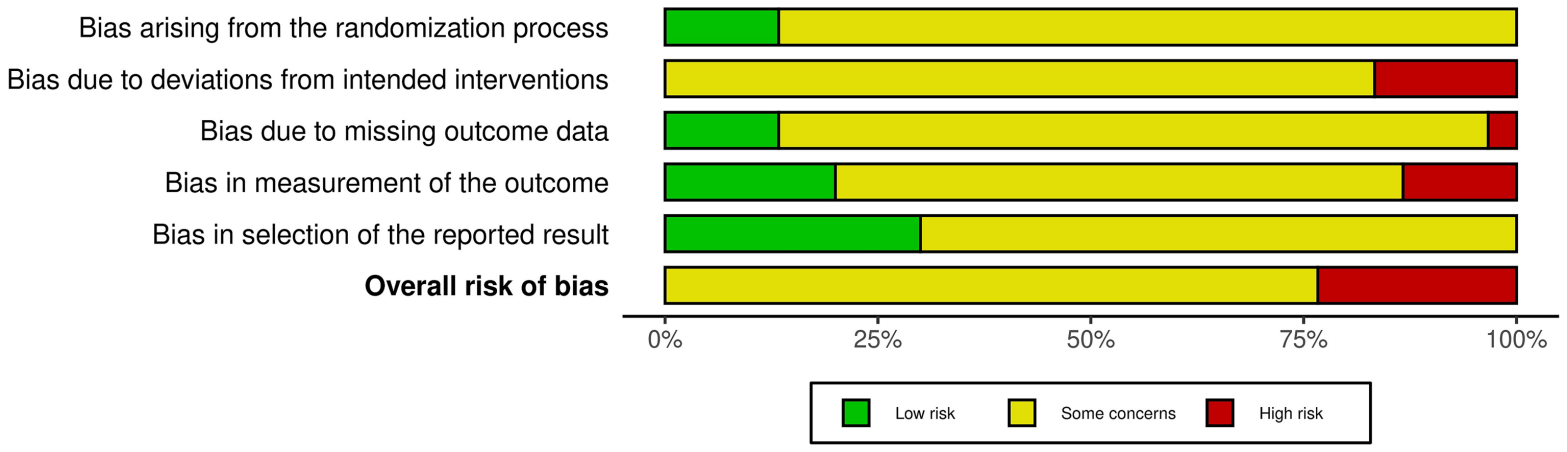
Risk of bias graph.

Figures 2, 3 present the risk-of-bias assessment of the included studies. Two reviewers independently assessed the 30 studies using the five RoB 2 domains. Overall, 23 studies were judged as having “some concerns” and 7 studies as having “high risk” of bias, while no study was rated as overall “low risk.” The main concerns were related to randomization reporting, deviations from intended interventions, missing outcome data, and outcome measurement.

Initial inter-rater agreement varied across RoB 2 domains. Before consensus discussion, Cohen’s κ was 0.12 for the randomization process, 0.31 for deviations from intended interventions, 0.05 for missing outcome data, 0.41 for measurement of the outcome, and 0.15 for selection of the reported result (Supplementary Table S3). These values indicated low initial agreement in several domains, particularly D1, D3, and D5. All disagreements were then reviewed item by item and resolved through discussion or third-reviewer adjudication before the final RoB 2 judgments were used in the analysis (see Supplementary File 1 for justifications for judgments and incomplete outcome data). Nevertheless, the low initial agreement suggested uncertainty in interpreting trial reporting, which reduced confidence in the risk-of-bias assessment and was considered when rating the GRADE certainty of evidence. Detailed domain-level RoB 2 judgments are provided in Supplementary Table S2.

### 3.3 Study characteristics

A total of 30 studies were included (Table 1), published between 2010 and 2025. Most studies were conducted in China, with others from the USA, Singapore, Canada, Spain, and South Korea. The average age of participants ranged from approximately 45 to 72 years. Sample sizes ranged from 23 to 200 individuals. The included studies involved both healthy and clinical populations, including patients with breast cancer, insomnia, COPD, bipolar disorder, and osteoporosis. This population diversity was considered an important source of clinical heterogeneity.

**Table 1:** Characteristics of the included randomized controlled trials.

Authors	Country	Age mean (SD) (Years)	Sample size, n (female)	Participants	Intervention and comparator	Frequency (sessions/week)	Duration (weeks)	Session length (min)	Outcome measure
Song et al. (2022)	China	64.15 (8.56)	40 (40)	Older adults with knee osteoarthritis	Modified Tai Chi vs. health education	3	12	60	SDS
Dong et al. (2013)	South Korea	68.5 (NR)	102 (102)	Older women living alone	Yijinjing vs. no-intervention control, Liuzijue vs. no-intervention control	3	12	60	GDS-15
Liao (2015)	China	63.1 (4.20)	80 (80)	Healthy older women	24-form Tai Chi vs. usual lifestyle	3	24	60	SCL-90-D, POMS-D
Ma et al. (2016)	China	60.6 (4.6)	78 (78)	Middle-aged and older women	Mawangdui Daoyin vs. no-intervention control	3	20	60	POMS-D
Mo and Wang (2016)	China	66.2 (4.3)	79 (79)	Older women	Tai Chi stick vs. no-intervention control	3	24	60	SCL-90-D
Xu et al. (2025)	China	54.44 (7.10)	100 (100)	Female breast cancer patients after surgery	ORTCC management combined with Baduanjin vs. usual rehabilitation care	5	12	20–30	HADS-D
Yang and Wang (2023)	China	48.32 (2.46)	80 (80)	Perimenopausal women with COPD	Baduanjin plus usual treatment vs. usual treatment	7	4	30	SDS
Ma et al. (2010)	China	49.19 (2.68)	100 (100)	Perimenopausal women	Baduanjin vs. no-intervention control	7	12	45	CES-D
Shan et al. (2025)	China	50.6 (3.1)	80 (80)	Perimenopausal women with insomnia	Baduanjin combined with Du meridian fumigation vs. Du meridian fumigation	7	4	15	SDS
Luo et al. (2021)	China	48.9 (3.5)	70 (70)	Female breast cancer patients undergoing chemotherapy	Baduanjin combined with five-element music therapy vs. usual care	14	4	30	SDS
Ma et al. (2011)	China	47.2 (2.5)	145 (145)	Perimenopausal women with depressive symptoms	Baduanjin vs. non-exercise control, Baduanjin vs. walking	5	12	45	CES-D
Wen et al. (2024)	China	45.3 (5.1)	128 (128)	Female breast cancer patients after modified radical mastectomy	Early rehabilitation through ERAS, when paired with the initial four variations of Baduanjin, compared to ERAS early rehabilitation alone.	7	12	30	SDS
Wei et al. (2022)	China	55 (3.5)	70 (70)	Female breast cancer patients undergoing chemotherapy	Baduanjin vs. wait-list control	5	12	30	HADS-D
Larkey et al. (2015)	USA	57.5 (8.8)	101 (101)	Postmenopausal breast cancer survivors with fatigue	Qigong/Tai Chi vs. sham qigong	1	12	60	BDI
Chen et al. (2013)	China	45.0 (8.1)	96 (96)	Female breast cancer patients receiving radiotherapy	Qigong vs. wait-list control	5	6	40	CES-D
Sun et al. (2019)	China	50.0 (9.7)	67 (67)	Breast cancer patients	Baduanjin combined with breathing and meditation vs. usual pharmacotherapy	7	4	30	SDS
Shi et al. (2025)	China	55.2 (6.3)	118 (113)	Breast cancer patients	Multimodal psychological support combined with Baduanjin vs. usual care	14	10	30	SCL-90-D
Ni et al. (2021)	China	69.1 (6.3)	162 (162)	Older female patients undergoing chemotherapy for breast tumors	Honghuang decoction combined with Baduanjin vs. Honghuang decoction	2	16	30	SDS
Li et al. (2022)	China	60.0 (5.0)	57 (57)	Postmenopausal women with osteoporosis	Erxian decoction combined with Baduanjin vs. Baduanjin, Erxian decoction combined with Baduanjin vs. Erxian decoction	5	16	45	SDS
Liu J. et al. (2025)	China	47.5 (1.8)	72 (72)	Perimenopausal women with depression Symptoms	Tai Chi vs. no-intervention control	5	12	60	SDS
Carcelén-Fraile et al. (2023)	Spain	69.7 (6.4)	125 (125)	Postmenopausal women	Qigong vs. control	2	12	60	HADS-D
Chang et al. (2024)	China	65.5 (3.5)	124 (124)	Older women	Long-duration Tai Chi vs. control	5	24	60	BDI
Chen (2013)	China	70.2 (7.5)	180 (89)	Community-dwelling older adults	Baduanjin vs. usual lifestyle	6	22	60	SCL-90-D
Zhao et al. (2015)	China	60.1 (6.5)	52 (29)	Middle-aged and older adults with mild depression	Tai Chi vs. usual lifestyle	3	52	30	GDS-15
Siu et al. (2025)	China	64.3 (6.2)	200 (161)	Middle-aged and older adults with chronic insomnia	Tai Chi vs. cognitive behavioral therapy for insomnia	2	12	60	HADS-D
Wen et al. (2023)	China	46.3 (9.2)	88 (21)	Patients with nasopharyngeal carcinoma after chemoradiotherapy	Baduanjin vs. usual care	5	12	40	PHQ-9
Park et al. (2023)	Canada	58.1 (9.4)	23 (15)	Middle-aged and older adults with bipolar disorder	Qigong/Tai Chi vs. light exercise	1	12	60	MADRS, QIDS-SR
Ma et al. (2018)	China	70.0 (10.6)	158 (49)	Community-dwelling older adults with hypertension	Group-based Tai Chi vs. usual care	4	24	60	CES-D
Liu Z. et al. (2025)	China	67.9 (4.6)	110 (68)	Older adults with sleep disorders and mild cognitive impairment	Tai Chi plus real rTMS vs. Tai Chi plus sham rTMS	5	6	60	HDRS
Tou et al. (2024)	Singapore	72.8 (7.0)	56 (49)	Community-dwelling older adults with low handgrip strength	Baduanjin vs. wait-list control (health education)	2.5	16	60	GDS-15

NR, Not Reported; COPD, Chronic Obstructive Pulmonary Disease; ORTCC, Orem’s Rehabilitation Theory-based Continuous Care; ERAS, Enhanced Recovery After Surgery; rTMS, repetitive Transcranial Magnetic Stimulation; SDS, Self-Rating Depression Scale; GDS-15, Geriatric Depression Scale-15; SCL-90-D, Symptom Checklist-90; POMS-D, POMS Depression-Dejection subscale; HADS-D, Hospital Anxiety and Depression Scale - Depression subscale; CES-D, Center for Epidemiologic Studies Depression Scale; BDI, Beck Depression Inventory; PHQ-9, Patient Health Questionnaire-9; MADRS, Montgomery-Asberg Depression Rating Scale; QIDS-SR, Quick Inventory of Depressive Symptomatology-Self-Report; HDRS, Hamilton Depression Rating Scale.

The interventions predominantly featured traditional Chinese mind–body exercises, such as Tai Chi, Baduanjin, Qigong, Yijinjing, Liuzijue, and Daoyin. Some studies combined these exercises with standard care, psychological support, music therapy, or Chinese medicine. These co-interventions may have contributed to the observed effects and could confound interpretation of the independent effect of mind–body exercises. Control groups received no intervention, were wait-listed, received standard care, health education, or alternative interventions. Exercise sessions occurred 1 to 14 times per week, with most studies reporting 3 to 7 sessions per week. Each session lasted 15 to 60 min, and the overall intervention period ranged from 4 to 52 weeks, with 12 weeks the most common duration. Outcomes were primarily assessed using established depression scales, including the Self-Rating Depression Scale (SDS), Geriatric Depression Scale-15 (GDS-15), Symptom Checklist-90 (SCL-90), the Depression-Dejection subscale of the Profile of Mood States (POMS), and other validated measures. In summary, the studies varied in intervention strategies, comparator conditions, and outcome assessments.

### 3.4 Multilevel Meta-analysis results

The primary analysis used post-intervention depression scores to assess the effects of traditional Chinese mind–body exercises on depressive symptoms in middle-aged and older adults. The initial full-data analysis incorporated 30 randomized controlled trials and 35 effect sizes. Given that several studies reported multiple effect sizes and the three-level random-effects model demonstrated a superior fit compared to the standard two-level model (AIC: 109.93 vs. 112.66), the former was selected as the primary analytical approach to account for interdependence among effect sizes. In the initial full-data model, the pooled estimate suggested a statistically significant reduction in depressive symptoms favoring the intervention group (SMD = −1.05, 95% CI: −1.49 to −0.62, *p* < 0.001). However, substantial heterogeneity was detected across studies (*Q* = 467.31, *p* < 0.001). Variance component analysis revealed an overall I^2^ of 96.2%, indicating considerable heterogeneity. The contributions to variance from study-level and within-study effect sizes were 88.3 and 7.9%, respectively, with corresponding variance proportions of 91.8 and 8.2% (Supplementary Figure S1). These results indicated that most heterogeneity arose from between-study differences, likely related to variations in participant characteristics, intervention design, comparator conditions, and outcome measurement.

**Figure 4:**
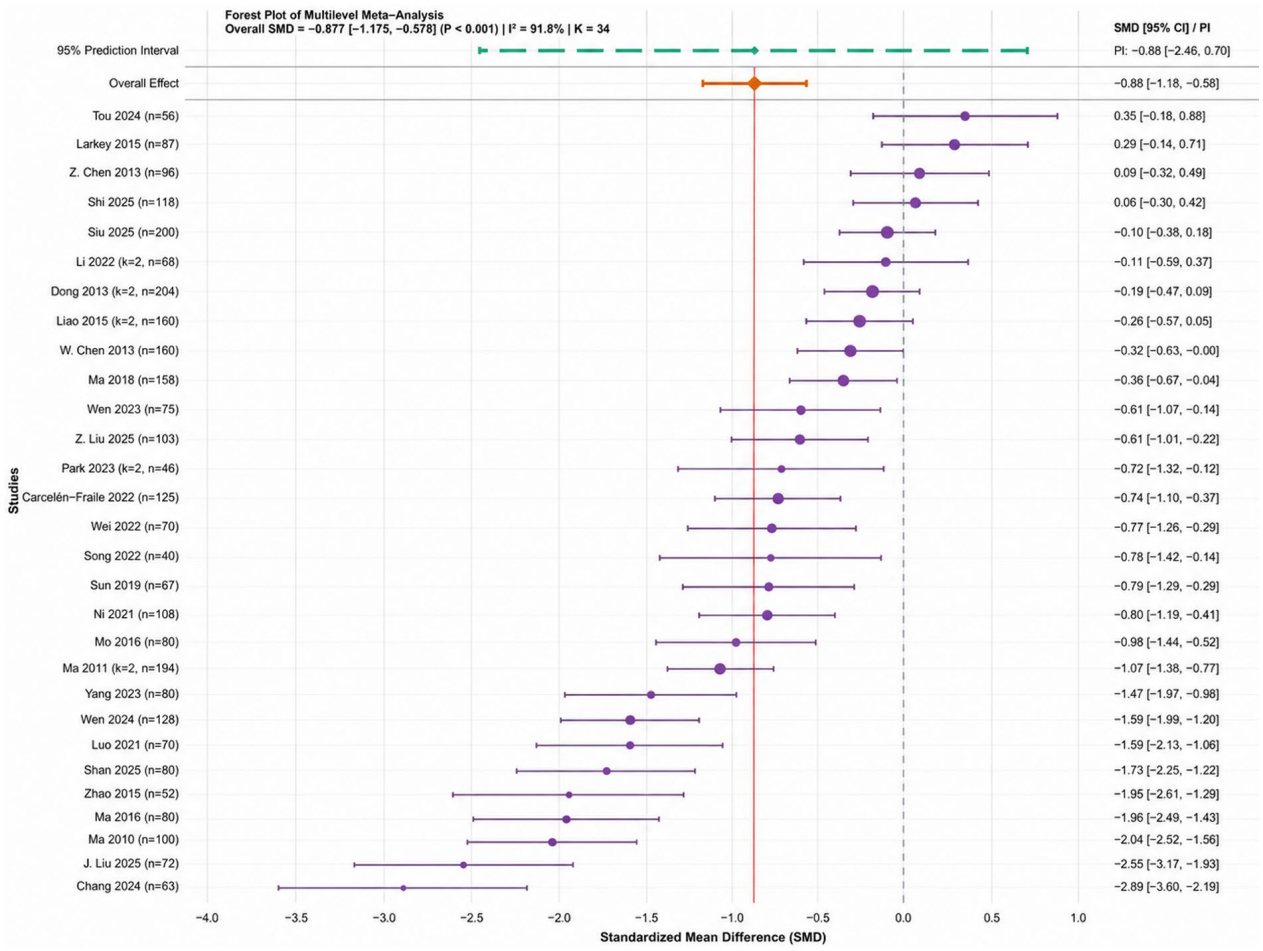
Forest plot of the effects of traditional Chinese mind–body exercises on depressive symptoms in middle-aged and older adults. The model included 29 studies and 34 effect sizes. SMD, standardized mean difference; CI, confidence interval; I^2^, heterogeneity statistic. Statistical significance was defined as a 95% confidence interval (95% CI) that did not include zero; k indicates the number of effect sizes rather than the number of independent trials.

The preliminary Egger’s regression analysis indicated a possible publication bias (*t* = 2.881, *p* = 0.004) (Egger et al., 1997). An influence assessment using standardized residuals and Cook’s distance revealed that Xu et al. (2025) exceeded the standardized residual threshold and had a Cook’s distance significantly above the predefined influence threshold (Viechtbauer and Cheung, 2010). Leave-one-out sensitivity analysis of the full-data model showed that the pooled effects remained significant after sequentially removing each effect size; SMDs ranged from −1.155 to −0.877, and all 95% CIs remained below zero. Therefore, an influence-adjusted sensitivity model excluding this study was also fitted, while the full-data model was retained and reported for transparency and comparison. In the influence-adjusted model, 29 studies and 34 effect sizes were included, and the pooled estimate also suggested a statistically significant reduction in depressive symptoms favoring the intervention group (SMD = −0.88, 95% CI: −1.18 to −0.58, *p* < 0.001) (Figure 4). Egger’s test was no longer significant after this adjustment (*t* = 1.083, *p* = 0.279). Furthermore, the other studies with elevated Cook’s distances did not meet the exclusion criteria and were retained in the analysis. The leave-one-out sensitivity assessment indicated that the pooled effect sizes ranged from −0.918 to −0.809, with no significant changes, supporting the robustness of the findings. The trim-and-fill analysis imputed 6 additional studies. After incorporating these imputed studies into the multilevel model, the pooled effect was attenuated but remained statistically significant (SMD = −0.518, 95% CI: −0.895 to −0.140). This attenuation suggests that the original pooled effect may have been overestimated to some extent, and therefore, the possibility of small-study effects or publication bias should be considered when interpreting the results. Comprehensive diagnostic and sensitivity results are provided in Supplementary Figures S2–S8.

**Figure 5:**
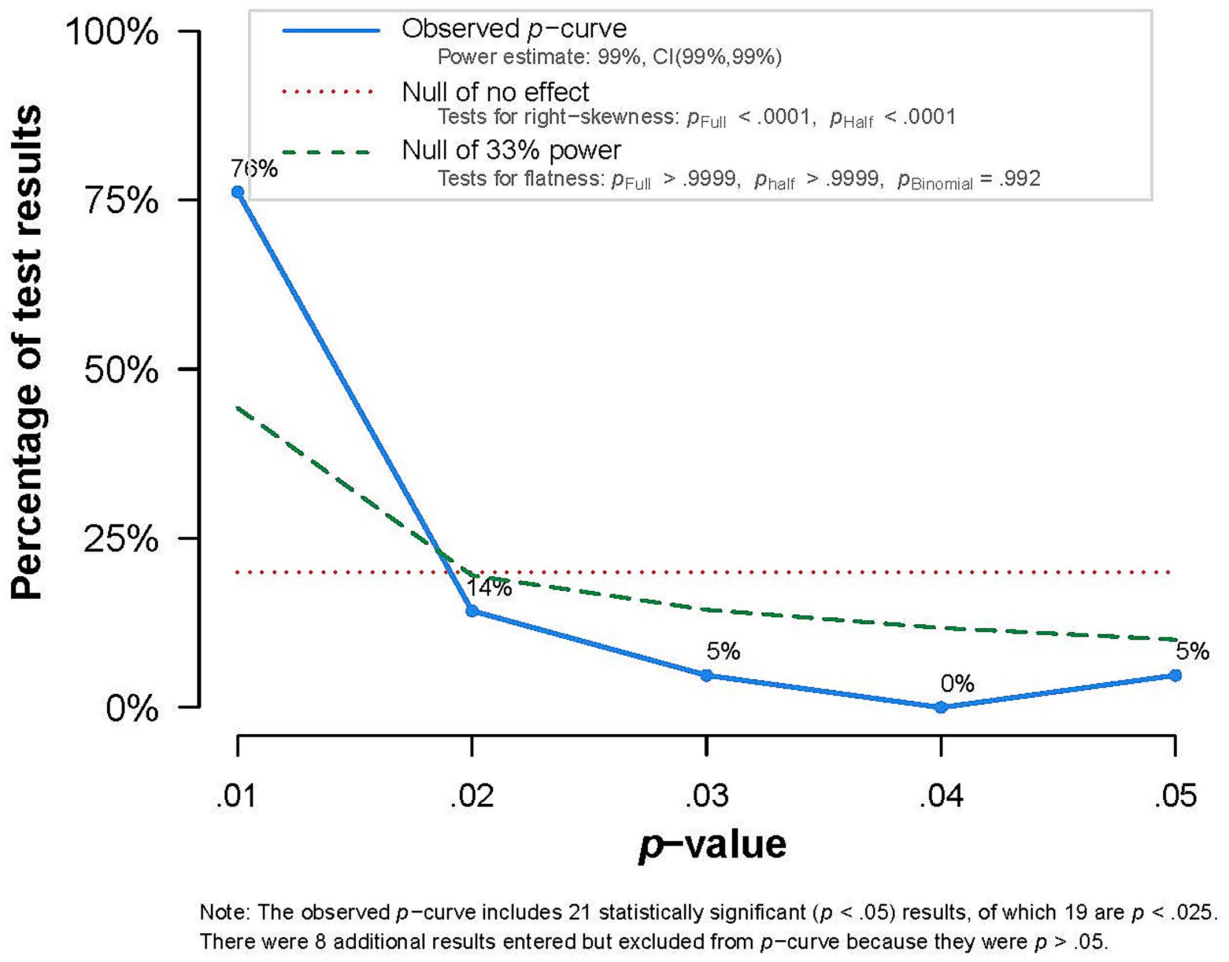
P-curve analysis of statistically significant effects. The p-curve includes statistically significant results (*p* < 0.05). A right-skewed distribution indicates evidential value and supports the presence of a true effect.

**Figure 6:**
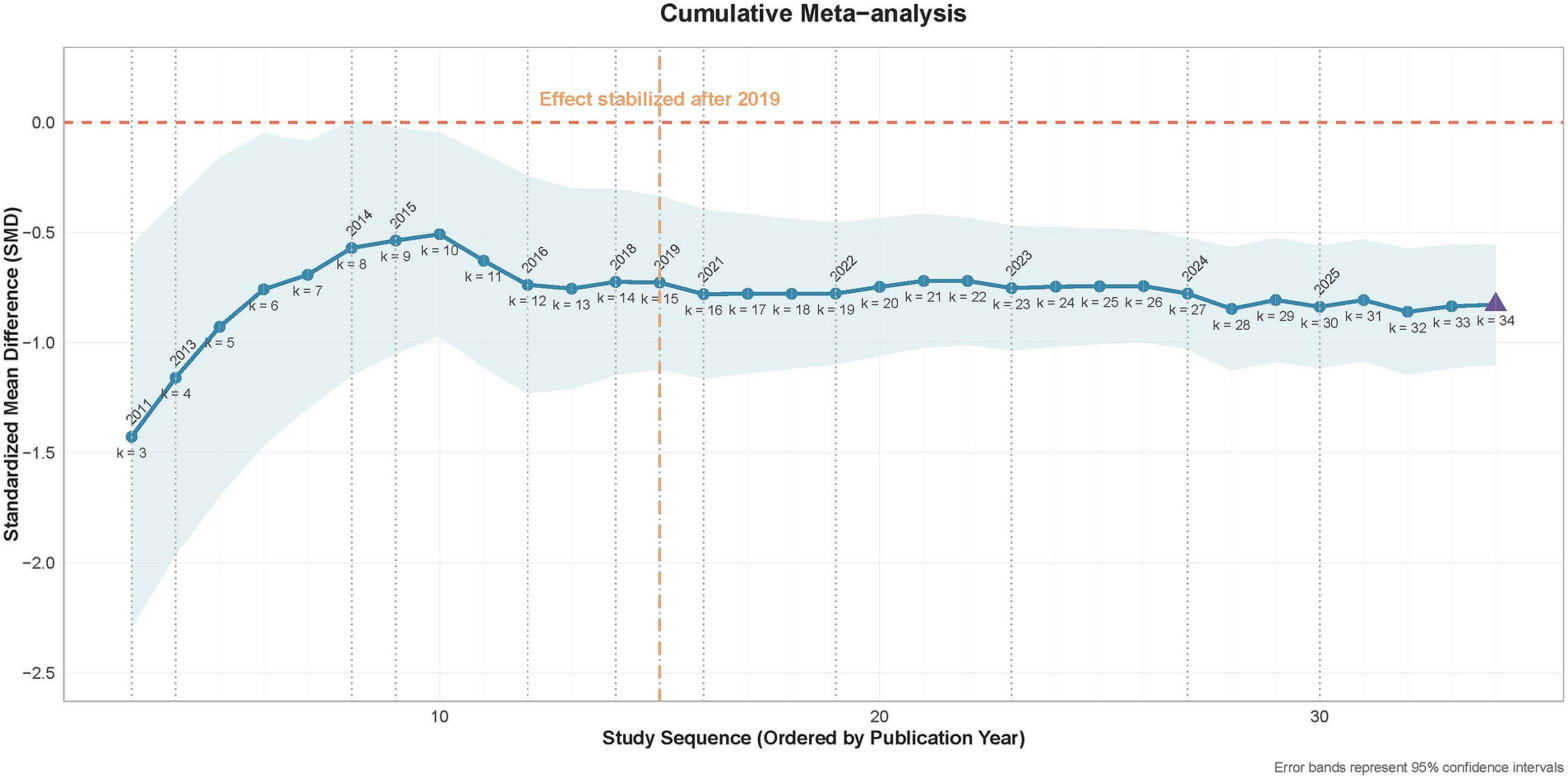
Cumulative meta-analysis of intervention effects over time. Studies were sequentially added according to publication year. Each point represents the pooled SMD after inclusion of the corresponding study, and the shaded area represents the 95% CI. Negative SMD values indicate greater reductions in depressive symptoms, favoring traditional Chinese mind–body exercise. The vertical dashed line marks the year after which the cumulative effect estimate became relatively stable.

Additional analyses provided supplementary support for the primary findings. The p-curve analysis showed that statistically significant outcomes were predominantly in the lower range of *p*-values, with 76% falling below *p* < 0.01. The overall distribution was right-skewed (*p* < 0.001), suggesting evidential value (Figure 5). The cumulative meta-analysis showed that the effect size stabilized after 2019 as the number of studies increased (Figure 6). Under the TSA assumptions above, the cumulative information size was 717.9, exceeding the required information size of 42.5, and the cumulative Z-curve crossed the monitoring boundary (Z = −5.97) (Supplementary Figure S9). This suggests that the accumulated evidence was sufficient to support the primary effect direction under the specified assumptions. However, because the required information size was driven by the large observed effect and substantial heterogeneity, the TSA result should be interpreted with caution. Furthermore, cluster-robust variance estimation (CRVE) using the CR2 method from the clubSandwich package was employed to assess robustness. The robust pooled effect at post-intervention was −0.877 (SE = 0.152), *t*(df = 27.9) = −5.76, *p* < 0.001, indicating that the outcome remained statistically significant after accounting for effect-size dependence. While these supplementary analyses supported the statistical consistency of the findings, the GRADE assessment rated the certainty of the evidence as low, primarily due to the absence of studies at low risk, several high-risk judgments, and substantial heterogeneity among studies (Supplementary Table S4). Therefore, the pooled effect should be interpreted as having low certainty rather than as definitive evidence of efficacy.

Overall, the influence-adjusted model showed a statistically significant but conventionally large effect, whose clinical interpretation should account for comparator type, co-interventions, heterogeneity, and risk of bias.

### 3.5 Subgroup analysis

**Figure 7:**
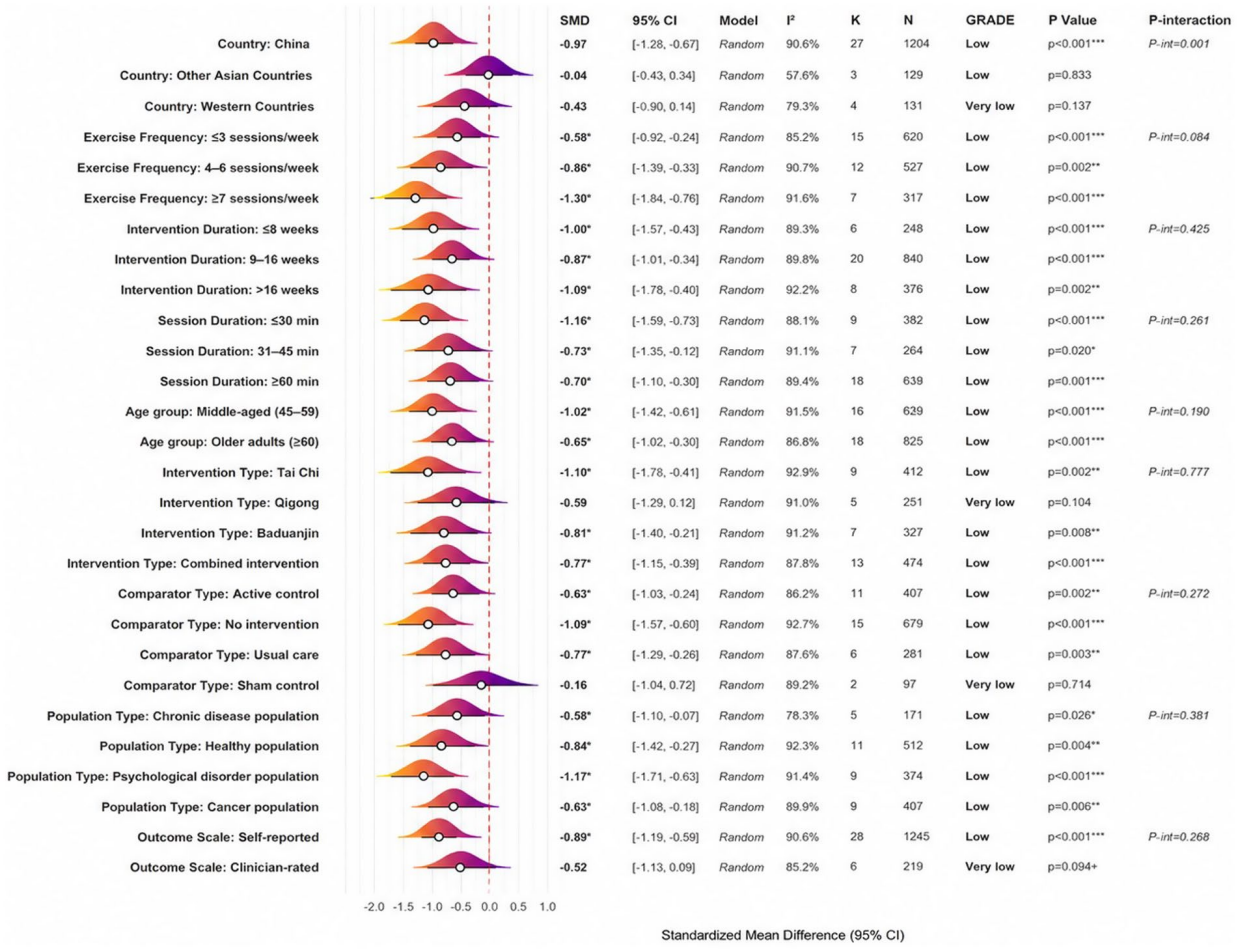
Subgroup analyses of intervention effects on depressive symptoms in middle-aged and older adults. k, number of effect sizes; N, total number of participants; *I*^2^, heterogeneity statistic; GRADE, certainty of evidence; P-int, test for between-subgroup differences. Negative SMD values indicate greater reductions in depressive symptoms, favoring traditional Chinese mind–body exercise. The model column indicates the meta-analytic model used for each subgroup. The *p*-value represents the statistical significance of the pooled effect within each subgroup, whereas P-int represents the statistical significance of differences between subgroups. Combined intervention refers to interventions in which traditional Chinese mind–body exercises is delivered alongside other active components or co-interventions, such as usual care, psychological support, music therapy, Chinese medicine, rehabilitation programs, or continuing-care management; *p < 0.05, **p < 0.01, ***p < 0.001.

To investigate potential effect modifiers, pre-specified subgroup analyses were conducted by study country, exercise frequency, intervention duration, session length, population type, age category, intervention type, comparator type, and outcome scale (Figure 7 and Supplementary Table S5). Among these variables, only the study country showed a statistically significant between-subgroup difference (P-interaction = 0.0009). Studies conducted in China showed a larger pooled effect (SMD = −0.97, *p* < 0.001; GRADE: Low), whereas studies from other regions did not show statistically significant effects. However, this finding should be interpreted cautiously because it may reflect differences in study design, intervention delivery, or the limited number of non-Chinese studies, rather than a true geographical effect.

For intervention and population characteristics, no significant subgroup differences were observed by exercise frequency, intervention duration, session length, population type, or age category (all P-interaction values > 0.05). Although interventions with ≥7 sessions/week, durations >16 weeks, and session lengths ≤30 min showed larger pooled effects descriptively, the between-subgroup differences were not statistically significant. Therefore, these results should not be interpreted as evidence that higher frequency, longer duration, or shorter session length is superior.

Similarly, no significant subgroup differences were observed by intervention type, comparator type, or outcome scale (P-interaction = 0.777, 0.272, and 0.288, respectively). Some subgroups, such as Tai Chi, Baduanjin, combined interventions, and self-reported scales, showed effects favoring traditional Chinese mind–body exercises; however, these descriptive findings do not establish a distinct advantage for any specific intervention modality, comparator condition, or measurement approach.

Overall, most subgroup estimates favored traditional Chinese mind–body exercises, but statistically significant subgroup differences were observed only by study country. Given the low certainty of evidence in several subgroup estimates and the small number of studies in some categories, these subgroup findings should be considered exploratory.

### 3.6 Multilevel meta-regression analysis

**Figure 8:**
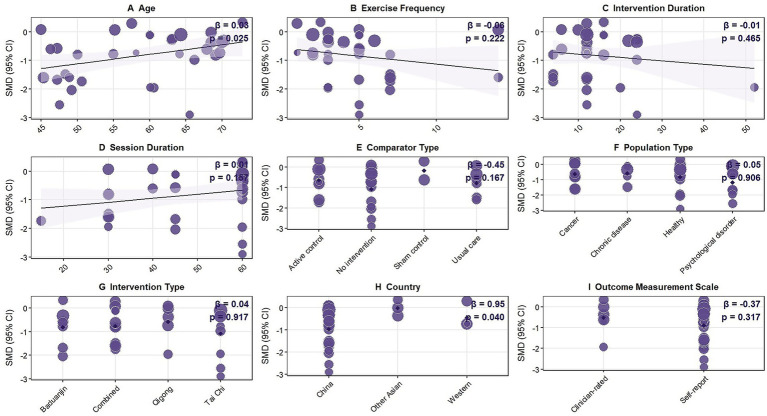
Multilevel meta-regression analyses of potential study-level moderators. Each point represents an individual effect size. **(A–D)** show continuous moderators with fitted regression lines and 95% CIs, while **(E–I)** show categorical moderators. *β* and *p*-values indicate the estimated moderator effect and its statistical significance, respectively.

Multilevel meta-regression was conducted to assess whether study-level characteristics explained variation in intervention effects (Figure 8). Overall, most study-level variables did not significantly moderate the effect of traditional Chinese mind–body exercises on depressive symptoms.

Among the continuous variables analyzed, only age showed a significant positive correlation with the effect size (*β* = 0.03, *p* = 0.025). Because negative SMD values indicate greater reductions in depressive symptoms, this positive coefficient suggests that older participants may have slightly smaller intervention effects. However, the coefficient was small, indicating limited practical significance. Exercise frequency (β = −0.06, *p* = 0.222), intervention duration (β = −0.01, *p* = 0.465), and session duration (β = 0.01, *p* = 0.157) showed no significant linear associations with the effect size. For categorical variables, no significant moderating effects were observed for comparator type, population type, intervention type, or outcome scale. The study country was the only categorical variable that reached statistical significance (β = 0.95, *p* = 0.040), but this result should be interpreted with caution because the number of studies from the “Other Asia” and “Western” regions was limited.

Some patterns observed in the subgroup analyses were not consistently supported by the meta-regression results, suggesting that these descriptive subgroup differences may have been influenced by other study-level factors or by imbalanced category distributions. Overall, the meta-regression analyses did not establish a clear dose–response relationship or identify reliable study-level moderators. Given the substantial heterogeneity, uneven variable distributions, and a limited number of studies in some categories, these findings should be regarded as exploratory rather than conclusive.

### 3.7 Nonlinear regression and dose–response analysis

**Figure 9:**
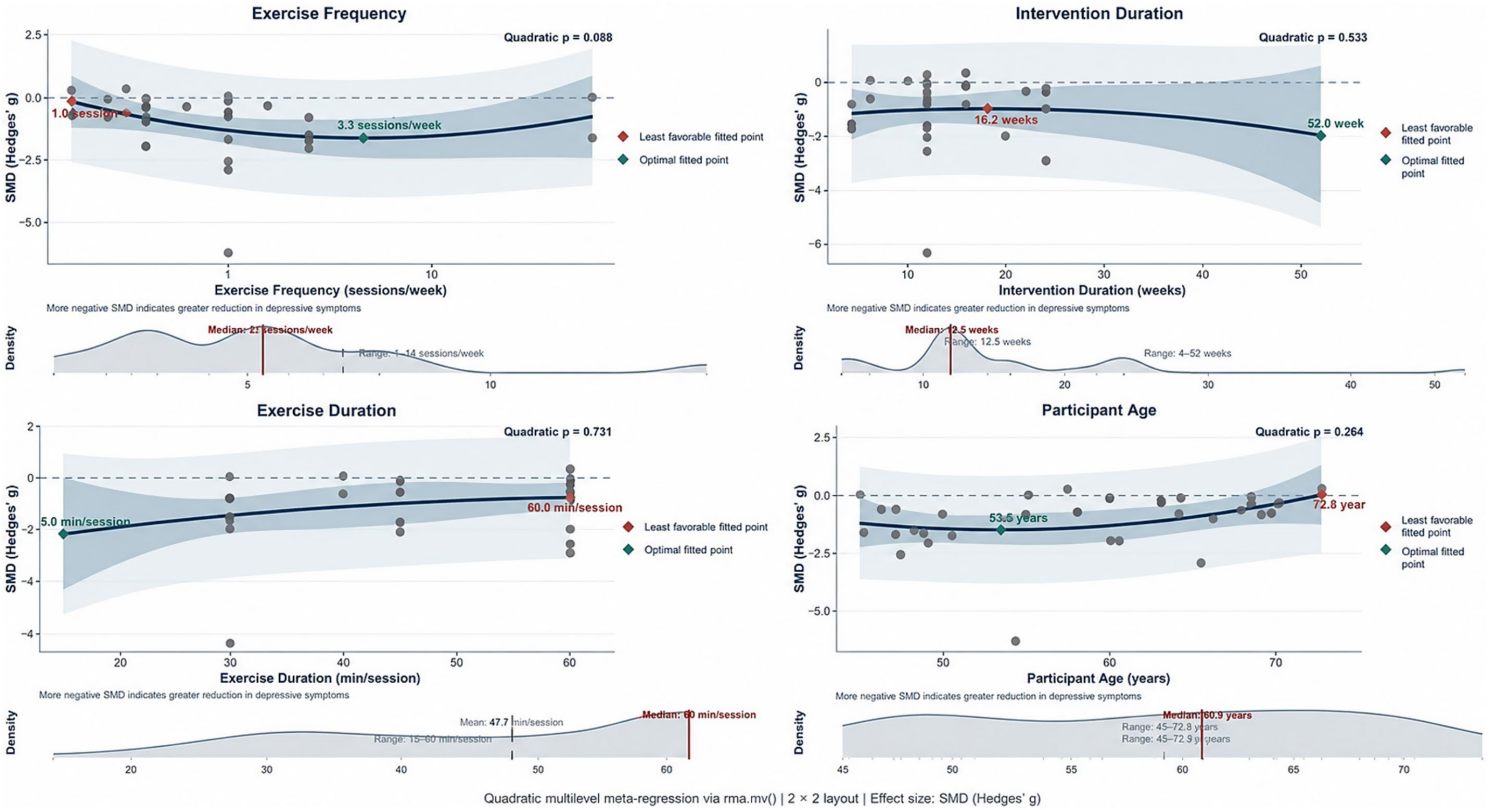
Multilevel nonlinear dose–response relationship between traditional Chinese mind–body exercises and depressive symptoms in middle-aged and older adults. Each point represents an individual effect size. The solid curve and shaded area indicate the fitted nonlinear relationship and 95% CI. The red triangle and green diamond indicate exploratory fitted reference points rather than evidence-based optimal doses.

**Figure 10:**
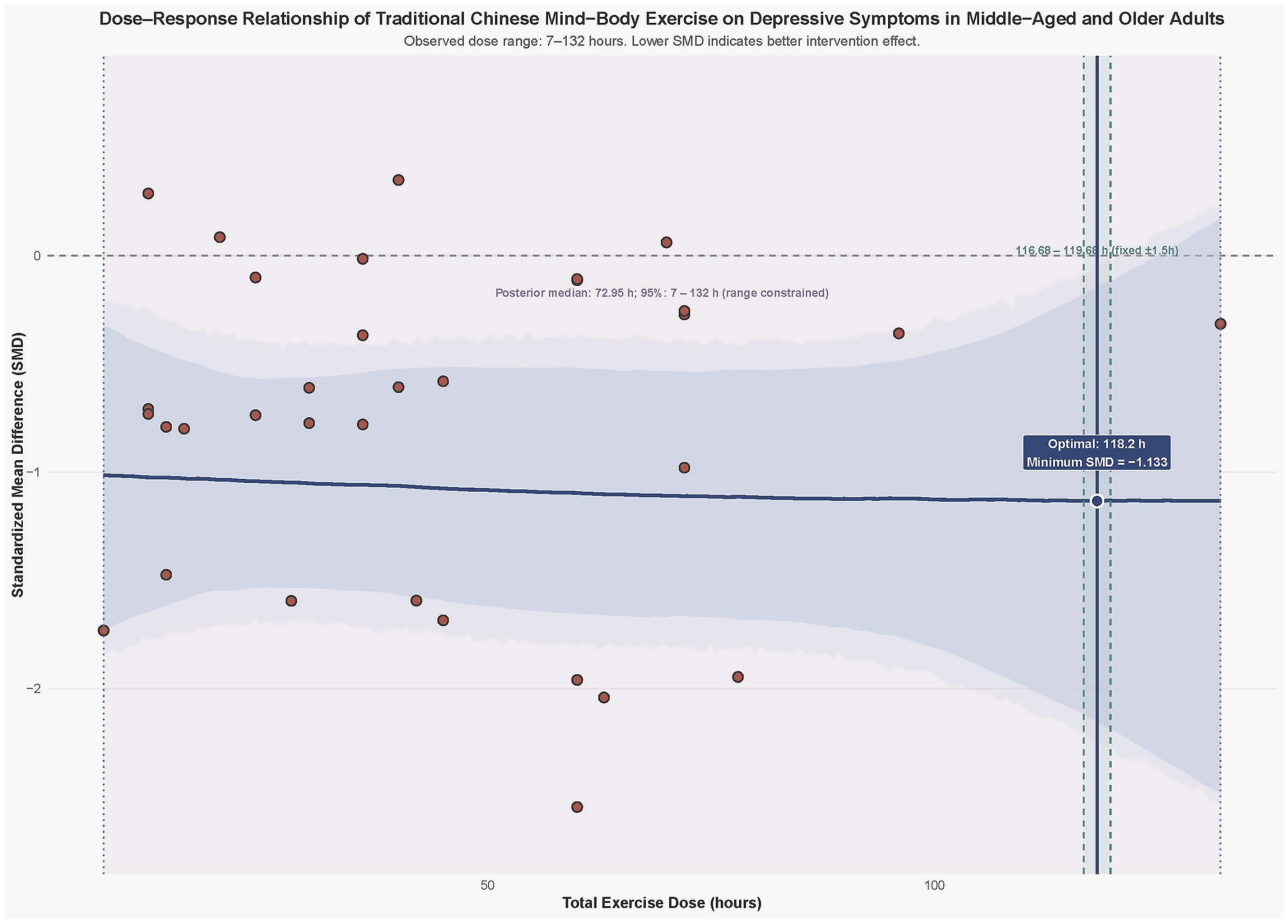
Bayesian multilevel dose–response relationship between total intervention dose and depressive symptoms. The estimated low point should be treated as exploratory because it was close to the upper boundary of the observed dose range and was associated with substantial uncertainty.

According to the multilevel nonlinear dose–response evaluation (Figure 9) and the Bayesian multilevel dose–response assessment (Figure 10), traditional Chinese mind–body exercises were associated with a general reduction in depressive symptoms at the pooled level. However, the dose–response patterns were inconsistent and did not support a statistically significant optimal dose. The posterior distribution of the exploratory low point and Bayesian model diagnostic results are provided in Supplementary Figures S10A–D. Nonlinear analysis revealed descriptive dose-related patterns for exercise frequency and intervention duration. However, none of the quadratic terms were statistically significant (all *p* > 0.05), indicating that no statistically supported nonlinear dose–response relationship or optimal dose could be identified.

The Bayesian analysis showed wide uncertainty in the estimated total exercise dose, with a posterior median of 72.95 h and a broad 95% posterior interval of 7 to 132 h. Because the exploratory low point was close to the upper boundary of the observed dose distribution, it should not be interpreted as an optimal dose.

In conjunction with subgroup analyses, increased exercise frequency (at least 7 sessions per week) and an extended intervention period (over 16 weeks) were associated with larger effect sizes at the descriptive level. Nonetheless, the observed differences between subgroups were not statistically significant, and these results were not consistently validated by meta-regression or nonlinear analyses. Consequently, the available evidence does not support the conclusion that higher-frequency or longer-duration interventions are more effective than other training approaches. These descriptive, dose-related findings may inform hypotheses for future trials, but should not be treated as evidence-based dose recommendations.

### 3.8 XGBoost machine learning extension analysis

The XGBoost-based analysis was conducted as an exploratory extension to assess the relative contributions of study-level features to predicted SMD values. Model performance was limited. Leave-one-study-out cross-validation yielded a low R^2^ of 0.022 (Supplementary Figure S11A), and the learning curve showed a clear gap between training and validation errors. Across training-set sizes, the validation RMSE remained relatively high, approximately 1.3–1.7, whereas the training RMSE remained much lower, approximately 0.10–0.20 (Supplementary Figure S11B). These findings indicate limited out-of-sample predictive capacity and possible overfitting. Therefore, the XGBoost and SHAP results should be interpreted as exploratory feature-importance signals rather than as evidence from a reliable predictive model. Additional model performance diagnostics and SHAP dependence plots are provided in Supplementary Figures S12, 13.

**Figure 11:**
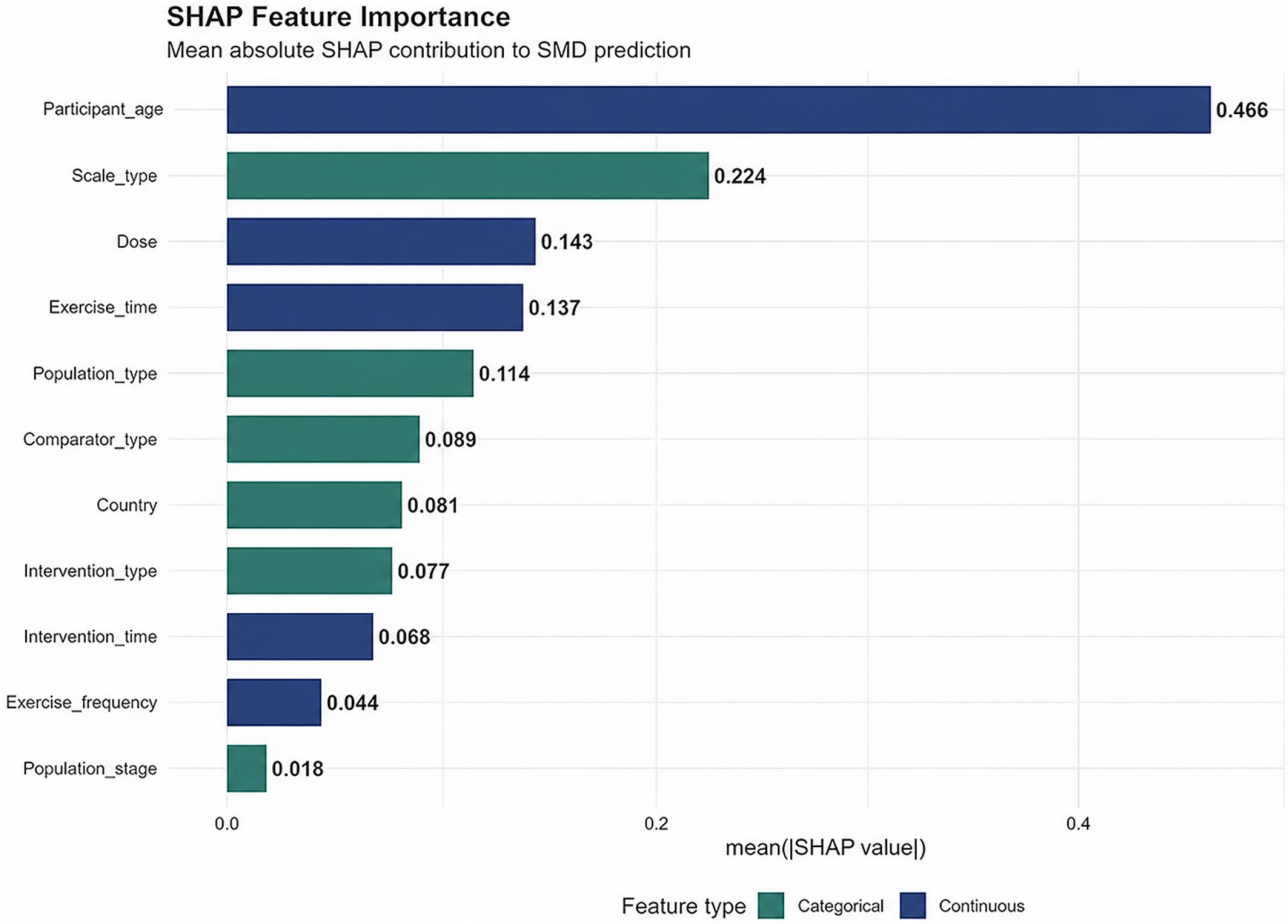
SHAP-based feature importance for predicted SMD in the exploratory XGBoost analysis. Feature importance is expressed as the mean absolute SHAP value, reflecting the average magnitude of each feature’s contribution to predicted SMD.

In the exploratory SHAP analysis, participant age had the highest mean absolute SHAP value (approximately 0.466), followed by scale type (approximately 0.224). However, given the limited sample size and the potential instability of feature-importance estimates, this ranking should be interpreted cautiously. Other variables, such as dose, exercise time, population type, comparator type, country, intervention type, and intervention time, had smaller mean absolute SHAP values, ranging from approximately 0.06 to 0.14. Exercise frequency and population stage had the lowest mean absolute SHAP values, each ≤0.05 (Figure 11).

**Figure 12:**
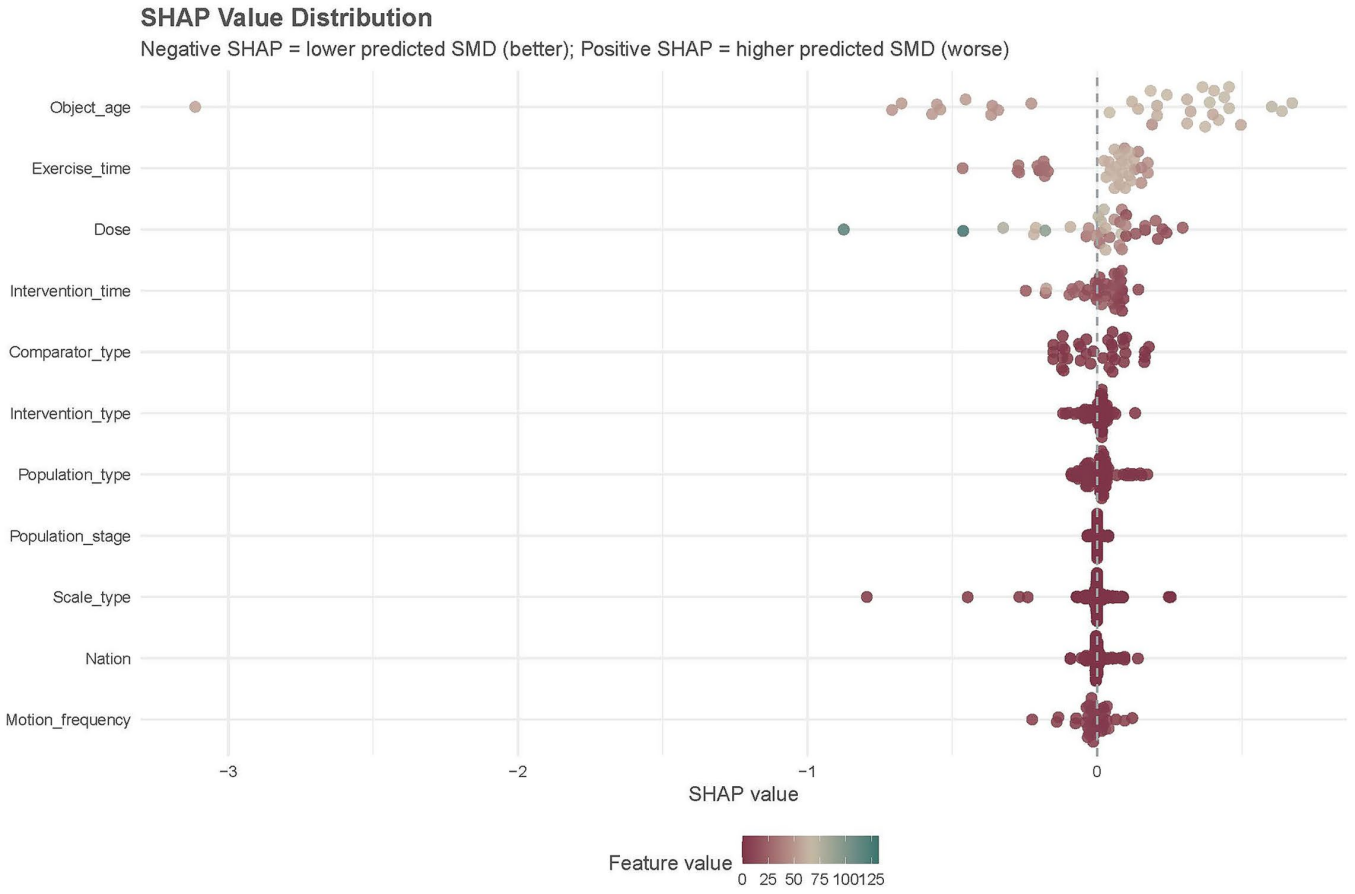
Distribution of SHAP values illustrating feature contributions to predicted SMD in the exploratory XGBoost analysis. Each dot represents an effect size. SHAP values indicate feature contributions to predicted SMD (negative = better, positive = worse), with color representing the feature value.

A deeper analysis of the distribution of SHAP values revealed that feature influence varied across value ranges (Figure 12). In general, negative SHAP values were associated with lower predicted SMD values, indicating greater improvement in depressive symptoms, whereas positive SHAP values were associated with less effective intervention outcomes. For instance, participant age and exercise duration exhibited both positive and negative SHAP distributions across multiple value ranges, suggesting that their effects may be characterized by nonlinear or interactive relationships rather than a straightforward directional link.

The machine learning extension provided supplementary feature importance information. In this model, variables such as age, intervention timing, and intervention type appeared to contribute to predicted SMD values. Overall, the XGBoost and SHAP results should be regarded as supplementary feature-importance signals rather than confirmatory predictive or causal evidence.

## 4 Discussion

### 4.1 Evidence summary

This study synthesized evidence from randomized controlled trials examining the effects of traditional Chinese mind–body exercises on depressive symptoms in middle-aged and older adults. The pooled estimate indicated a statistically significant reduction in depressive symptoms (SMD = −0.88, 95% CI: −1.18 to −0.58, *p* < 0.001), but the certainty of the evidence was low due to substantial heterogeneity and risk-of-bias concerns. Although the pooled effect was significant, the 95% prediction interval was wide and crossed the null value (−2.45 to 0.70). This suggests that, given substantial heterogeneity, the benefit may not be consistent across all future settings and may be small or absent in some contexts. Therefore, the pooled mean effect should be interpreted cautiously. Sensitivity and supplementary analyses generally supported the statistical consistency of the main effect, whereas subgroup analysis and meta-regression provided only exploratory evidence regarding potential moderators. Similarly, the dose–response and XGBoost/SHAP analyses should be interpreted as exploratory extensions and did not establish confirmatory mechanisms, causal predictors, or optimal intervention parameters.

The results of this study are broadly consistent with previous research on traditional Chinese mind–body exercises and depressive symptoms (Lavretsky et al., 2022; Zeng et al., 2023; Zhu et al., 2024). Lavretsky et al. found in a randomized controlled trial that both Tai Chi Chih and health education contributed to symptom improvement when combined with standard antidepressant therapy, with additional benefits observed in some health-related outcomes in the Tai Chi group (Lavretsky et al., 2022). Zeng et al. (2023)reported that Tai Chi significantly reduced depressive symptoms in middle-aged and older adults, although considerable variability was observed across studies, particularly in intervention duration and total exercise exposure. Zhu et al. (2024) further focused on adults aged 60 years and older with depression and found that Tai Chi was more effective than no exercise or health education in reducing depressive symptoms; however, the authors also noted that evidence remained insufficient to determine the optimal frequency, duration, or overall dose of Tai Chi exercise. More recently, Bayesian dose-oriented evidence has suggested that exercise parameters may be relevant to psychological outcomes, but optimal dose recommendations remain uncertain and may vary by population, comparator condition, and exercise modality (Chen et al., 2025). The pooled effect observed in the present study appears larger than that reported in some previous reviews. This difference may be partly explained by the inclusion of both clinical and non-clinical populations with depressive symptoms, the use of no-intervention or usual-care controls in several included trials, and the presence of co-interventions in some studies, which may amplify between-group effects compared with active comparator conditions.

### 4.2 Potential mechanisms

The observed effects of traditional Chinese mind–body exercises may be partly explained by their combined influence on emotional regulation, sleep and stress-related processes, and sport psychology factors. Exercises such as Tai Chi, Qigong, and Baduanjin typically combine gentle movement, breathing regulation, attentional focus, and body awareness, which have been associated with relaxation and emotional regulation (Wang et al., 2023). Previous studies have also suggested that traditional Chinese mind–body exercises may be linked to improved sleep quality (Nabizadehchianeh et al., 2024; Lin et al., 2026), lower perceived stress (Zou et al., 2018a), greater social engagement (Wu et al., 2024), and enhanced self-confidence (Chair et al., 2025). From a sport psychology perspective, these exercises may also be understood as structured health-related physical activity that integrates bodily movement with attentional and emotional regulation (Wayne and Kaptchuk, 2008; Larkey et al., 2009). For middle-aged and older adults, the relatively low physical demands, routine structure, and group practice format may support adherence, self-efficacy, and social participation (Koren et al., 2021; Wu et al., 2024). These pathways may help explain the pooled effect, but because they were not directly tested in this meta-analysis, they should be treated as hypotheses rather than as confirmed mechanisms.

### 4.3 Dose–response evidence

The dose–response analyses supported an exploratory rather than confirmatory interpretation. Although some dose-related subgroups showed descriptive differences in effect size, subgroup analysis, meta-regression, nonlinear dose–response modeling, and Bayesian analysis did not provide consistent evidence for a specific optimal dose. The estimated low point for total exercise dose was near the upper boundary of the observed dose distribution and was associated with substantial uncertainty, making it unsuitable for defining an optimal dose. The XGBoost and SHAP analyses further suggested that age, scale type, total dose, and session length may be relevant study-level features, but these findings should be treated as supplementary signals of feature importance rather than as confirmatory predictors or causal evidence (Ponce-Bobadilla et al., 2024). Overall, the current evidence suggests that dose-related factors may influence intervention effects, but it does not support firm dose recommendations.

### 4.4 Practical applications

From a practical standpoint, traditional Chinese mind–body exercises are generally mild, affordable, adaptable, and conducive to long-term participation, making them a potential complementary, non-drug approach to alleviating depressive symptoms in middle-aged and older adults. This study used a three-level meta-analysis to address effect-size interdependence and incorporated exploratory dose–response and machine-learning analyses to provide preliminary insights into dose-related factors and potential moderators. However, because no statistically supported optimal dose was identified, specific training parameters should not be interpreted as evidence-based prescriptions. Instead, common intervention characteristics observed across the included studies, such as at least 3 sessions per week, intervention durations of 8–18 weeks, and session lengths of 30–60 min, may serve as hypothesis-generating ranges for future trials. Practical implementation should be individualized based on health status, exercise experience, comorbidities, and professional guidance.

### 4.5 Study limitations

This study has several limitations. First, most included studies were conducted in China, which may limit the generalizability of the findings to other cultural and healthcare settings. Second, substantial heterogeneity was observed across studies, possibly related to differences in participant characteristics, intervention composition, comparator conditions, exercise dose, and outcome measures. Some studies evaluated traditional Chinese mind–body exercises combined with usual care or other active components; therefore, the pooled effect may partly reflect the contribution of co-interventions rather than the independent effect of traditional Chinese mind–body exercises alone. Third, the included studies did not consistently report whether participants had a clinically diagnosed depressive disorder. Thus, the findings should be interpreted primarily as effects on depressive symptoms rather than as direct evidence for treating clinically diagnosed depression. Fourth, the dose–response and XGBoost/SHAP analyses were based on aggregate study-level data and a limited number of effect sizes; these findings should therefore be regarded as exploratory and hypothesis-generating. Future studies should use more rigorous randomized designs, clearly report baseline depressive status and co-intervention components, and include longer follow-up assessments. Trials in more diverse cultural and healthcare settings are also needed to clarify generalizability and determine the clinically meaningful dose of traditional Chinese mind–body exercises.

## 5 Conclusion

This multilevel meta-analysis suggests that traditional Chinese mind–body exercises may reduce depressive symptoms among middle-aged and older adults. However, the certainty of the evidence was low due to substantial heterogeneity and concerns about the risk of bias. Exploratory subgroup analyses, meta-regression, nonlinear dose–response analyses, and machine learning analyses suggested that intervention effects may be influenced by multiple factors, including participant age, population characteristics, comparator type, and intervention design, rather than by a single dosage factor. Therefore, traditional Chinese mind–body exercises may serve as a complementary, non-pharmacological approach for this population, but further well-designed RCTs are needed to clarify intervention parameters and long-term effects.

## Data Availability

The original contributions presented in the study are included in the article/Supplementary material, further inquiries can be directed to the corresponding author.

## References

[ref1] Alexopoulos G. S. (2019). Mechanisms and treatment of late-life depression. Transl. Psychiatry 9:188. doi: 10.1038/s41398-019-0514-6, 31383842 PMC6683149

[ref2] Assink M. Wibbelink C. J. M. (2016). Fitting three-level meta-analytic models in R: a step-by-step tutorial. Quant. Methods Psychol. 12, 154–174. doi: 10.20982/tqmp.12.3.p154

[ref3] Aziz R. Steffens D. C. (2013). What are the causes of late-life depression? Psychiatr. Clin. North Am. 36, 497–516. doi: 10.1016/j.psc.2013.08.001, 24229653 PMC4084923

[ref4] Carcelén-Fraile M. d. C. Hita-Contreras F. Martínez-Amat A. Loureiro V. B. de Loureiro N. E. M. Jiménez-García J. D. . (2023). Impact of qigong exercises on the severity of the menopausal symptoms and health-related quality of life: a randomised controlled trial. Eur. J. Sport Sci. 23, 656–664. doi: 10.1080/17461391.2022.2044915, 35179431

[ref5] Chair S. Y. Law B. M. H. Chan A. W. K. Gao R. (2025). The effect of the tai chi intervention on self-esteem and self-confidence perception in adult populations: a systematic review and meta-analysis. BMC Nurs. 24:174. doi: 10.1186/s12912-025-02792-9, 39953472 PMC11829346

[ref6] Chan S. H. Chan W. W. Chao J. Y. Chan P. K. (2020). A randomized controlled trial on the comparative effectiveness of mindfulness-based cognitive therapy and health qigong-based cognitive therapy among Chinese people with depression and anxiety disorders. BMC Psychiatry 20:590. doi: 10.1186/s12888-020-02994-2, 33317481 PMC7734764

[ref7] Chang S. Cheng L. Liu H. (2024). Effects of three-duration tai-chi exercises on depression and sleep quality in older women. Eur. Geriatr. Med. 15, 1141–1148. doi: 10.1007/s41999-024-00981-4, 38693298

[ref8] Chen W. (2013). Effects of Baduanjin on mental health of urban community-dwelling older adults. Chin. J. Gerontol. 33, 3472–3473. doi: 10.3969/j.issn.1005-9202.2013.14.106

[ref9] Chen S. Luo L. Yuan Y. (2025). Optimal tai chi dose for improving anxiety, depression, and sleep quality in older adults: a Bayesian meta-analysis. Clin. Rehabil. 39, 1170–1180. doi: 10.1177/02692155251355083, 40625025

[ref10] Chen Z. Meng Z. Milbury K. Bei W. Zhang Y. Thornton B. . (2013). Qigong improves quality of life in women undergoing radiotherapy for breast cancer: results of a randomized controlled trial. Cancer 119, 1690–1698. doi: 10.1002/cncr.27904, 23355182 PMC3852682

[ref11] Chen Y. Tang Y. Zhou Z. (2024). The effects of tai chi and Baduanjin on breast cancer patients: systematic review and meta-analysis of randomized controlled trials. Front. Oncol. 14:1434087. doi: 10.3389/fonc.2024.1434087, 39529823 PMC11551136

[ref12] Cohen J. (1960). A coefficient of agreement for nominal scales. Educ. Psychol. Meas. 20, 37–46. doi: 10.1177/001316446002000104

[ref13] Crippa A. Orsini N. (2016). Multivariate dose-response meta-analysis: the dosresmeta R package. J. Stat. Softw. 72, 1–15. doi: 10.18637/jss.v072.c01

[ref14] Dong L. Lee J.-B. Kim Y.-K. Kim Y.-S. (2013). The effects of health qigong training of elderly single women on pain consciousness and depression. Int. J. Appl. Sports Sci. 25:118–126. doi: 10.24985/ijass.2013.25.2.118

[ref15] Donovan N. J. Blazer D. (2020). Social isolation and loneliness in older adults: review and commentary of a national academies report. Am. J. Geriatr. Psychiatry 28, 1233–1244. doi: 10.1016/j.jagp.2020.08.005, 32919873 PMC7437541

[ref16] Egger M. Smith G. D. Schneider M. Minder C. (1997). Bias in meta-analysis detected by a simple, graphical test. BMJ 315, 629–634. doi: 10.1136/bmj.315.7109.629, 9310563 PMC2127453

[ref17] Feng H. Li Y. Wang Q. Tao Y. Wang Z. (2025). The effects of traditional chinese exercises on anxiety and depression in adults: a systematic review and network meta-analysis. Front. Public Health 13:1582923. doi: 10.3389/fpubh.2025.1582923, 40270736 PMC12016670

[ref18] Guyatt G. H. Oxman A. D. Vist G. E. Kunz R. Falck-Ytter Y. Alonso-Coello P. . (2008). GRADE: an emerging consensus on rating quality of evidence and strength of recommendations. BMJ 336, 924–926. doi: 10.1136/bmj.39489.470347.AD, 18436948 PMC2335261

[ref19] Hamza T. Cipriani A. Furukawa T. A. Egger M. Orsini N. Salanti G. (2021). A Bayesian dose–response meta-analysis model: a simulations study and application. Stat. Methods Med. Res. 30, 1358–1372. doi: 10.1177/0962280220982643, 33504274 PMC8209313

[ref20] Hu T. Zhao X. Wu M. Li Z. Luo L. Yang C. . (2022). Prevalence of depression in older adults: a systematic review and meta-analysis. Psychiatry Res. 311:114511. doi: 10.1016/j.psychres.2022.114511, 35316691

[ref21] Jiang C. Zhu F. Qin T. (2020). Relationships between chronic diseases and depression among middle-aged and elderly people in China: a prospective study from CHARLS. Curr. Med. Sci. 40, 858–870. doi: 10.1007/s11596-020-2270-5, 33123901

[ref22] Koren Y. Leveille S. You T. (2021). Tai chi interventions promoting social support and interaction among older adults: a systematic review. Res. Gerontol. Nurs. 14, 126–137. doi: 10.3928/19404921-20210325-02, 34039148 PMC9836824

[ref23] Laks J. Engelhardt E. (2010). Peculiarities of geriatric psychiatry: a focus on aging and depression. CNS Neurosci. Ther. 16, 374–379. doi: 10.1111/j.1755-5949.2010.00196.x, 20875046 PMC6493790

[ref24] Larkey L. Jahnke R. Etnier J. Gonzalez J. (2009). Meditative movement as a category of exercise: implications for research. J. Phys. Act. Health 6, 230–238. doi: 10.1123/jpah.6.2.230, 19420401

[ref25] Larkey L. K. Roe D. J. Weihs K. L. Jahnke R. Lopez A. M. Rogers C. E. . (2015). Randomized controlled trial of qigong/tai chi easy on cancer-related fatigue in breast cancer survivors. Ann. Behav. Med. 49, 165–176. doi: 10.1007/s12160-014-9645-4, 25124456 PMC4329282

[ref26] Lau J. Antman E. M. Jimenez-Silva J. Kupelnick B. Mosteller F. Chalmers T. C. (1992). Cumulative meta-analysis of therapeutic trials for myocardial infarction. N. Engl. J. Med. 327, 248–254. doi: 10.1056/NEJM199207233270406, 1614465

[ref27] Lavretsky H. Milillo M. M. Kilpatrick L. Grzenda A. Wu P. Nguyen S. A. . (2022). A randomized controlled trial of tai chi chih or health education for geriatric depression. Am. J. Geriatr. Psychiatry 30, 392–403. doi: 10.1016/j.jagp.2021.07.008, 34404606 PMC10208421

[ref28] Li K. Yu H. Lin X. Su Y. Gao L. Song M. . (2022). The effects of ER xian decoction combined with baduanjin exercise on bone mineral density, lower limb balance function, and mental health in women with postmenopausal osteoporosis: a randomized controlled trial. Evid. Based Complement. Altern. Med. 2022:8602753. doi: 10.1155/2022/8602753 PMC9262512 35815264

[ref29] Liao J. (2015). Effects of 24-week tai chi exercise on mental health of urban elderly women. Chin. J. Gerontol. 35, 7232–7233. doi: 10.3969/j.issn.1005-9202.2015.24.128

[ref30] Lin J. Gao Y. F. Guo Y. Li M. Zhu Y. You R. . (2022). Effects of qigong exercise on the physical and mental health of college students: a systematic review and meta-analysis. BMC Complementary Med. Ther. 22:287. doi: 10.1186/s12906-022-03760-5, 36348349 PMC9641907

[ref31] Lin W.-T. Lee B.-O. Wirojratana V. Taplo Y. M. Tonapa S. I. (2026). Beneficial effects of community-based traditional chinese exercises on sleep disturbance among older adults: systematic review, meta-analysis, and meta-regression of randomized controlled trials. J. Transcult. Nurs. 37, 419–432. doi: 10.1177/10436596251412642, 41553843

[ref32] Liu J. Si J. Zhao W. (2025). Investigation of the effect of tai chi training on depressive symptoms in perimenopausal women on the basis of serum kynurenine metabolites. Exp. Aging Res. 51, 331–349. doi: 10.1080/0361073X.2024.2377427, 39023066

[ref33] Liu Z. Zhang L. Bai L. Guo Z. Gao J. Lin Y. . (2025). Repetitive transcranial magnetic stimulation and tai chi chuan for older adults with sleep disorders and mild cognitive impairment: a randomized clinical trial. JAMA Netw. Open 8:e2454307. doi: 10.1001/jamanetworkopen.2024.54307, 39792383 PMC12548080

[ref34] Luo Y. Chen S. Shao L. Xie J. Zhu A. Zhu C. . (2021). Effects of baduanjin combined with wuxing music on anxiety and depression in patients with breast cancer chemotherapy. Chin. Community Dr. 37, 181–181. doi: 10.3969/j.issn.1007-614x.2021.19.085

[ref35] Ma S. Chen C. Zhao Y. Guo Q. Li C. (2011). Effect of baduanjin on quality of life in perimenopausal women with functional constipation in the community. Chin. J. Gerontol. 31, 926–928. doi: 10.3969/j.issn.1005-9202.2011.06.006

[ref36] Ma S. Dou N. Chen C. Zhao Y. Li S. (2010). Effect of the traditional baduanjin exercise in women with peri-menopausal syndrome and depression. Chin. Gen. Pract. 13, 2864–2865. doi: 10.3969/j.issn.1007-9572.2010.25.030

[ref37] Ma Z. Wang B. Xi B. (2016). Effects of health qigong mawangdui daoyinshu exercise on mood state and anxiety level in middle-aged and elderly women. Chin. J. Gerontol. 36, 3248–3249. doi: 10.3969/j.issn.1005-9202.2016.13.075

[ref38] Ma C. Zhou W. Tang Q. Huang S. (2018). The impact of group-based tai chi on health-status outcomes among community-dwelling older adults with hypertension. Heart Lung 47, 337–344. doi: 10.1016/j.hrtlng.2018.04.007, 29778251

[ref39] Manandhar K. Risal A. Shrestha O. Manandhar N. Kunwar D. Koju R. . (2019). Prevalence of geriatric depression in the Kavre district, Nepal: findings from a cross sectional community survey. BMC Psychiatry 19:271. doi: 10.1186/s12888-019-2258-5, 31481037 PMC6724336

[ref40] Mo G. Wang B. (2016). Effects of health qigong taiji yangsheng staff exercise on mood state and mental health in elderly women. Chin. J. Gerontol. 36, 5401–5403. doi: 10.3969/j.issn.1005-9202.2016.21.086

[ref41] Moher D. Shamseer L. Clarke M. Ghersi D. Liberati A. Petticrew M. . (2015). Preferred reporting items for systematic review and meta-analysis protocols (PRISMA-P) 2015 statement. Syst. Rev. 4:1. doi: 10.1186/2046-4053-4-1, 25554246 PMC4320440

[ref42] Nabizadehchianeh G. Morsaljahan S. Walker D. I. Nosratabad T. H. (2024). The effectiveness of tai chi chuan exercise on depression, sleep quality, and mental health. doi: 10.21203/rs.3.rs-4253409/v1

[ref43] Ni T. Sun L. Gao L. Wang N. Li M. Fu J. . (2021). Effect of honghuang decoction combined with baduanjin on negative emotions, fatigue degree, and quality of life in elderly patients with breast tumors undergoing chemotherapy. J. Clin. Pathol. Res. 41, 2012–2017. doi: 10.3978/j.issn.2095-6959.2021.09.007

[ref44] Niarchou E. Roberts L. Naughton B. D. (2024). What is the impact of antidepressant side effects on medication adherence among adult patients diagnosed with depressive disorder: a systematic review. J. Psychopharmacol. (Oxf. Engl.) 38, 127–136. doi: 10.1177/02698811231224171, 38344912 PMC10863360

[ref45] Park H. Rigas C. Ibrahim M. Su C.-L. Eyler L. Thomas Z. . (2023). Efficacy of virtually-delivered qigong/tai chi for depression in middle-and older-age adults with bipolar disorder (QT-BD): a pilot randomized controlled trial during the COVID-19 pandemic. J. Affect. Disord. Rep. 13:100604. doi: 10.1016/j.jadr.2023.100604

[ref46] Ponce-Bobadilla A. V. Schmitt V. Maier C. S. Mensing S. Stodtmann S. (2024). Practical guide to SHAP analysis: explaining supervised machine learning model predictions in drug development. Clin. Transl. Sci. 17:e70056. doi: 10.1111/cts.70056, 39463176 PMC11513550

[ref47] Qi F. Soh K. G. Mohd Nasirudddin N. J. Mai Y. (2022). Effects of taichi on physical and psychological health of college students: a systematic review. Front. Physiol. 13:1008604. doi: 10.3389/fphys.2022.1008604, 36246109 PMC9562146

[ref48] Qiu T. Zhang G. Zhou F. Jiang H. (2024). The socialization of older adults with depression in group qigong training, − taijiquan, Baduanjin, yi jin jing. Curr. Psychol. 43, 30417–30428. doi: 10.1007/s12144-024-06636-8

[ref49] Read J. R. Sharpe L. Modini M. Dear B. F. (2017). Multimorbidity and depression: a systematic review and meta-analysis. J. Affect. Disord. 221, 36–46. doi: 10.1016/j.jad.2017.06.009, 28628766

[ref50] Sadavoy J. (2009). An integrated model for defining the scope of psychogeriatrics: the five Cs. Int. Psychogeriatr. 21, 805–812. doi: 10.1017/S104161020999010X, 19505355

[ref51] Shan B. Tang H. Wang S. (2025). Effect of Ba duan jin combined with du mai fumigation on improving sleep quality and negative emotions in perimenopausal insomnia patients. Prog. Mod. Biomed. 25, 2351–2356. doi: 10.13241/j.cnki.pmb.2025.14.014

[ref52] Shi T. Zhou M. He Y. Lin T. Zhu X. Lu J. (2025). Intervention effect of multi-track psychological support combined with traditional Chinese medicine Baduanjin on patients with breast cancer. Hebei J. Tradit. Chin. Med. 47, 567–571. doi: 10.3969/j.issn.1002-2619.2025.04.009

[ref53] Simonsohn U. Nelson L. D. Simmons J. P. (2014). P-curve and effect size: correcting for publication bias using only significant results. Perspect. Psychol. Sci. 9, 666–681. doi: 10.1177/1745691614553988, 26186117

[ref54] Siu P. M. Yu D. J. Yu A. P. Recchia F. Li S. X. Chan R. N. . (2025). Tai chi or cognitive behavioural therapy for treating insomnia in middle aged and older adults: randomised non-inferiority trial. BMJ 391:e084320. doi: 10.1136/bmj-2025-084320, 41297969 PMC12651913

[ref55] Song J. Wei L. Cheng K. Lin Q. Xia P. Wang X. . (2022). The effect of modified tai chi exercises on the physical function and quality of life in elderly women with knee osteoarthritis. Front. Aging Neurosci. 14:860762. doi: 10.3389/fnagi.2022.860762, 35721018 PMC9204295

[ref56] Stahl S. T. Kincman J. Karp J. F. Anne Gebara M. (2023). Psychosocial interventions to improve adherence in depressed and anxious older adults prescribed antidepressant pharmacotherapy: a scoping review. Ther. Adv. Psychopharmacol. 13:1212322. doi: 10.1177/20451253231212322, 38022838 PMC10664420

[ref57] Sterne J. A. C. Savović J. Page M. J. Elbers R. G. Blencowe N. S. Boutron I. . (2019). RoB 2: a revised tool for assessing risk of bias in randomised trials. BMJ l4898:l4898. doi: 10.1136/bmj.l4898, 31462531

[ref58] Stetler C. Miller G. E. (2011). Depression and hypothalamic-pituitary-adrenal activation: a quantitative summary of four decades of research. Psychosom. Med. 73, 114–126. doi: 10.1097/PSY.0b013e31820ad12b, 21257974

[ref59] Sun J. Miao W. Kang C. Yang C. Li X. Gao J. . (2019). Effect of Baduanjin combined with resting and meditation on negative emotion and immune function of breast cancer patients. Tradit. Chin. Med. Chin. Mater. Med. 4, 131–132. doi: 10.19347/j.cnki.2096-1413.201928053

[ref60] Szymkowicz S. M. Gerlach A. R. Homiack D. Taylor W. D. (2023). Biological factors influencing depression in later life: role of aging processes and treatment implications. Transl. Psychiatry 13:160. doi: 10.1038/s41398-023-02464-9, 37160884 PMC10169845

[ref61] Tou N. X. Goh S. F. Harding S. Tsao M. A. Ng T. P. Wee S.-L. (2024). Effectiveness of community-based Baduanjin exercise intervention for older adults with varying frailty status: a randomized controlled trial. Eur. Rev. Aging Phys. Act. 21:28. doi: 10.1186/s11556-024-00363-6, 39390362 PMC11465814

[ref62] Van Houwelingen H. C. Arends L. R. Stijnen T. (2002). Advanced methods in meta-analysis: multivariate approach and meta-regression. Stat. Med. 21, 589–624. doi: 10.1002/sim.1040, 11836738

[ref63] Van Poelgeest E. P. Pronk A. C. Rhebergen D. Van Der Velde N. (2021). Depression, antidepressants and fall risk: therapeutic dilemmas—a clinical review. Eur. Geriatr. Med. 12, 585–596. doi: 10.1007/s41999-021-00475-7, 33721264 PMC8149338

[ref64] Viechtbauer W. Cheung M. W. (2010). Outlier and influence diagnostics for meta-analysis. Res. Synth. Methods 1, 112–125. doi: 10.1002/jrsm.11, 26061377

[ref65] Wang G. Liu Y. Peng C. Shen T. Du B. Yi L. (2025). Physical and psychological impacts of tai chi on college students and the determination of optimal dose: a systematic review and meta-analysis of randomized controlled trials. Eur. J. Integr. Med. 76:102450. doi: 10.1016/j.eujim.2025.102450

[ref66] Wang J. S. Meng J. Guo Z. Z. Jiang C. (2023). Effects of mind-body exercise on sleep quality: a systematic review. Chinese Journal of Rehabilitation Theory and Practice, 29:205–213. doi: 10.3969/j.issn.1006-9771.2023.02.009

[ref67] Wayne P. M. Kaptchuk T. J. (2008). Challenges inherent to t’ai chi research: part I— t’ai chi as a complex multicomponent intervention. J. Altern. Complement. Med. 14, 95–102. doi: 10.1089/acm.2007.7170A, 18199021

[ref68] Wei X. Yuan R. Yang J. Zheng W. Jin Y. Wang M. . (2022). Effects of Baduanjin exercise on cognitive function and cancer-related symptoms in women with breast cancer receiving chemotherapy: a randomized controlled trial. Support Care Cancer 30, 6079–6091. doi: 10.1007/s00520-022-07015-4, 35416502

[ref69] Wen L. Chen X. Cui Y. Zhang M. Bai X. (2023). Effects of Baduanjin exercise in nasopharyngeal carcinoma patients after chemoradiotherapy: a randomized controlled trial. Support Care Cancer 31:79. doi: 10.1007/s00520-022-07548-8, 36562869

[ref70] Wen C. Jiang W. Li R. Zhang X. Zhou T. (2024). Study on the application effect of early rehabilitation training based on the concept of enhanced recovery after surgery combined with the first four types of Baduanjin in patients after modified radical mastectomy for breast cancer. Prog. Mod. Biomed. 24, 264–267, 279. doi: 10.13241/j.cnki.pmb.2024.02.011

[ref71] Wetterslev J. Thorlund K. Brok J. Gluud C. (2008). Trial sequential analysis may establish when firm evidence is reached in cumulative meta-analysis. J. Clin. Epidemiol. 61, 64–75. doi: 10.1016/j.jclinepi.2007.03.013, 18083463

[ref72] Wu Z. Schimmele C. M. Chappell N. L. (2012). Aging and late-life depression. J. Aging Health 24, 3–28. doi: 10.1177/0898264311422599, 21956098

[ref73] Wu B. Xiong G. Zhang P. Ma X. (2024). Effects of tai chi, Ba duan jin, and walking on the mental health status of urban older people living alone: the mediating role of social participation and the moderating role of the exercise environment. Front. Public Health 12:1294019. doi: 10.3389/fpubh.2024.1294019, 38389938 PMC10881673

[ref74] Xu Z. Li Y. Mei X. Li Y. Meng X. (2025). Application of ORTCC management combined with Baduanjin in postoperative rehabilitation of breast cancer patients. Beijing J. Tradit. Chin. Med. 44, 697–701. doi: 10.16025/j.1674-1307.2025.06.005

[ref75] Yang X. Wang Y. (2023). Effect of Baduanjin on pulmonary function and mental state in perimenopausal women with chronic obstructive pulmonary disease. J. Women Child. Health 2, 61–63. doi: 10.3969/j.issn.2097-115X.2023.5.whbb202305020

[ref76] Zeng L. Zhao X. Yu Y. Hu T. Li C. Wu M. . (2023). Effects of tai chi on depression of middle-aged and older adults: an updated systematic review and meta-analysis. BMC Complementary Med. Ther. 23:382. doi: 10.1186/s12906-023-04207-1, 37891569 PMC10605936

[ref77] Zhao G. Cheng R. Jie C. Sun Q. Chen R. Wu B. . (2015). Effect of shadowboxing on mild depression among middle-aged and senior people. Chin. J. Conval. Med. 24, 452–454. doi: 10.13517/j.cnki.ccm.2015.05.002

[ref78] Zhu F. Wang Y. Yin S. Liu J. Zhong Y. Li L. (2024). The effect of tai chi on elderly depression: a systematic review and meta-analysis of randomized controlled trials. Front. Psychol. 15:1489384. doi: 10.3389/fpsyg.2024.1489384, 39679159 PMC11637854

[ref79] Zou L. Sasaki J. E. Wei G.-X. Huang T. Yeung A. S. Neto O. B. . (2018a). Effects of mind–body exercises (tai chi/yoga) on heart rate variability parameters and perceived stress: a systematic review with meta-analysis of randomized controlled trials. J. Clin. Med. 7:404. doi: 10.3390/jcm7110404, 30384420 PMC6262541

[ref80] Zou L. Yeung A. Quan X. Hui S. S.-C. Hu X. Chan J. S. M. . (2018b). Mindfulness-based baduanjin exercise for depression and anxiety in people with physical or mental illnesses: a systematic review and meta-analysis. Int. J. Environ. Res. Public Health 15:321. doi: 10.3390/ijerph15020321, 29439556 PMC5858390

