# Supplementary material for: Effects of traditional Chinese mind–body exercises on depressive symptoms in middle-aged and older adults: a multilevel meta-analysis with exploratory dose–response and machine learning analyses
Source: Front Psychol. 2026 Jul 13;17:1887582. doi: 10.3389/fpsyg.2026.1887582 (PMC13402133; doi:10.3389/fpsyg.2026.1887582)
Supplement: Supplementary file 1 [file Supplementary_file_1.DOCX]

**1.ROB2 Assessment for Song et al., 2022**

D1 — Bias arising from the randomization process​

R1 (Low risk): The document explicitly describes the random sequence generation method: "the participants were randomly assigned... using a computer-generated random number," indicating a standardized and reproducible process.

R2 (Low risk): Allocation concealment is clearly detailed: "A random number was placed in a sealed opaque envelope, which was opened for each subject separately after signing the informed consent form," effectively preventing selection bias.

Consensus: Low risk​

Rationale: Both random sequence generation and allocation concealment are rigorously described, and baseline groups are well-balanced, indicating minimal bias risk.

D2 — Bias due to deviations from the intended interventions​

R1 (Some concerns): The intervention (modified Tai Chi) is behavioral, making blinding of participants impossible, which may influence adherence or behavior; however, attendance rates were high (Tai Chi group 88%, control group 81%).

R2 (Some concerns): Assessor blinding is stated ("assessors were blinded to group assignment"), but the primary outcome (WOMAC) is subjective (patient-reported), and lack of participant blinding could introduce performance or detection bias, especially for self-reported measures.

Consensus: Some concerns​

Rationale: The inability to blind participants and the reliance on subjective primary outcomes introduce potential bias, though assessor blinding and high adherence mitigate some risks.

D3 — Bias due to missing outcome data​

R1 (Low risk): Missing data rates are low (total completion: 35/40, 87.5%; Tai Chi group 90%, control group 85%), with attrition primarily due to time conflicts or health issues.

R2 (Some concerns): The document states intention-to-treat (ITT) analysis ("all analyses were based on the intention to treat") but provides no details on methods for handling missing data (e.g., imputation or sensitivity analysis).

R3 (Some concerns): Although attrition is <15% and ITT is used, the lack of methodological clarity for missing data handling introduces uncertainty.

Consensus: Some concerns​

Rationale: Low attrition supports low risk, but insufficient details on ITT implementation (e.g., how missing data were incorporated) lead to some concerns.

D4 — Bias in measurement of the outcome​

R1 (Some concerns): The primary outcome (WOMAC) is subjective (patient-reported), and while assessors were blinded, the lack of participant blinding may introduce expectation bias.

R2 (Some concerns): Secondary outcomes include a mix of objective (e.g., TUG time) and subjective measures (e.g., SF-36, SAS). Assessor blinding protects objective measures, but subjective outcomes remain vulnerable.

Consensus: Some concerns​

Rationale: Although assessor blinding is in place, the primary outcome's subjectivity and lack of participant blinding dominate, raising concerns about measurement bias.

D5 — Bias in selection of the reported result​

R1 (Low risk): The trial was prospectively registered in the Chinese Clinical Trial Registry (ChiCTR2000040721), with registration prior to participant enrollment.

R2 (Low risk): All predefined outcomes (e.g., WOMAC, BBS, TUG, SF-36) are reported comprehensively, including non-significant results at follow-up, with no evidence of selective reporting.

Consensus: Low risk​

Rationale: Prospective registration and alignment between registered and reported outcomes indicate low risk of selective reporting.

Overall (Consensus): Some concerns​

Rationale: The worst-rated domains are D2, D3, and D4 (all "Some concerns"). Key issues include the subjective nature of the primary outcome and lack of participant blinding (D2 and D4), combined with vague missing data methods (D3). However, the low risk in randomization (D1) and reporting (D5), along with assessor blinding and high adherence, support the study's overall rigor. The biases do not invalidate the results but warrant cautious interpretation.

**2.ROB2 Assessment for Dong et al., 2013**

D1 — Bias arising from the randomization process​

R1 (Some concerns): The document states that participants were "randomly placed into three groups" but provides no description of the random sequence generation method (e.g., computer-generated, random number table). The lack of detail on how randomization was performed introduces uncertainty.

R2 (Some concerns): No information is provided on allocation concealment (e.g., sealed envelopes or central randomization). The absence of safeguards against selection bias raises concerns.

Consensus: Some concerns​

Rationale: The lack of details on random sequence generation, allocation concealment, and baseline comparability introduces significant uncertainty about the randomization process.

D2 — Bias due to deviations from the intended interventions​

R1 (Some concerns): The intervention (Health Qigong) is behavioral, and blinding of participants was not feasible, potentially affecting adherence. However, the document does not report attendance rates or adherence monitoring.

R2 (Some concerns): The document does not mention whether outcome assessors were blinded. The outcomes (pain consciousness and depression) are subjective self-reported measures, making them vulnerable to detection bias if assessors were unblinded.

Consensus: Some concerns​

Rationale: High risk of performance and detection bias due to the behavioral nature of the intervention, subjective outcomes, and absence of blinding details.

D3 — Bias due to missing outcome data​

R1 (Some concerns): Initial recruitment included 114 participants, but 12 were excluded due to "insincere responses or missing questions," leaving 102 for analysis (attrition rate: 10.5%). Reasons for exclusion are vague, and it is unclear whether these exclusions introduced bias.

R2 (High risk): The document does not mention the use of intention-to-treat (ITT) analysis or methods for handling missing data (e.g., imputation). Analyses appear per-protocol, as excluded participants were not included in the final analysis.

R3 (Some concerns): The attrition rate (>10%) and lack of ITT analysis suggest potential bias, particularly if exclusions were related to outcomes (e.g., participants with worse pain/depression dropping out).

Consensus: Some concerns​

Rationale: Attrition rate and lack of ITT analysis introduce concerns about bias from missing data, though the rate is marginally acceptable.

D4 — Bias in measurement of the outcome​

R1 (Some concerns): The primary outcomes (pain consciousness via Roland-Morris Back scale, depression via geriatric depression scale) are subjective self-reported measures. Although the tools are validated, their subjectivity increases susceptibility to measurement bias.

R2 (Some concerns): No information is provided on whether assessors were trained to standardize outcome assessments or if protocols were consistently applied. The document notes that assistants helped participants with physical/cognitive limitations, which may have introduced variability.

Consensus: Some concerns​

Rationale: Subjective outcomes and unclear assessment protocols dominate the risk of bias in this domain.

D5 — Bias in selection of the reported result​

R1 (High risk): The document does not mention prospective trial registration (e.g., in a clinical trials registry), making it impossible to verify whether all planned outcomes were reported.

R2 (Low risk): All outcomes described in the methods (pain consciousness and depression) are fully reported, including between-group comparisons (e.g., YiJinJing vs. LiuZiJue).

Consensus: Some concerns​

Rationale: While results appear comprehensively reported, the lack of prospective registration introduces uncertainty about selective reporting.

Overall (Consensus): Some concerns​

Rationale: All domains (D1–D5) are rated "Some concerns" or higher. Critical issues include poor randomization reporting, lack of blinding, subjective outcomes, attrition without ITT analysis, and no prospective registration. While the study addresses an important population, methodological limitations necessitate cautious interpretation of the results.

**3.ROB2 Assessment for Liao et al., 2015**

D1 — Bias arising from the randomization process​

R1 (Some concerns): The document states that participants were "randomly divided" into groups but provides no description of the random sequence generation method (e.g., computer-generated, random number table, or drawing lots). The lack of methodological details introduces uncertainty.

R2 (Some concerns): No information is provided on allocation concealment (e.g., sealed envelopes or central randomization). The absence of safeguards against selection bias raises concerns.

Consensus: Some concerns​

Rationale: The lack of details on random sequence generation and allocation concealment introduces uncertainty, though baseline comparability partially mitigates risk.

D2 — Bias due to deviations from the intended interventions​

R1 (Some concerns): The intervention (Tai Chi) is behavioral, and blinding of participants was not feasible, potentially affecting adherence. However, the intervention was supervised ("under the guidance of experimental personnel"), which may standardize delivery.

R2 (Some concerns): The document does not mention whether outcome assessors were blinded. The outcomes (SCL-90 and POMS scores) are subjective self-reported measures, and lack of assessor blinding may introduce detection bias.

Consensus: Some concerns​

Rationale: High risk of performance and detection bias due to the behavioral intervention, subjective outcomes, and absence of blinding details.

D3 — Bias due to missing outcome data​

R1 (Low risk): The document reports 80 participants enrolled, with all 80 included in the analysis (no dropouts mentioned). The attrition rate appears to be 0%.

R2 (Some concerns): No intention-to-treat (ITT) analysis or methods for handling missing data are described, though the absence of attrition may render this less critical. Statistical analyses used t-tests, suggesting per-protocol analysis.

R3 (Low risk): With no missing data, the risk of bias from attrition is minimal.

Consensus: Some concerns​

Rationale: While no attrition reduces risk, the lack of explicit ITT analysis or missing data methods introduces theoretical uncertainty.

D4 — Bias in measurement of the outcome​

R1 (Some concerns): The primary outcomes (SCL-90 and POMS scores) are subjective self-reported measures. Although the scales are validated (reliability 0.80–0.92), their subjectivity increases susceptibility to measurement bias.

R2 (Some concerns): No information is provided on whether assessors were trained to standardize assessments or if protocols were consistently applied. The document notes that scales were administered but does not detail procedures.

Consensus: Some concerns​

Rationale: Subjective outcomes and unclear assessment protocols dominate the risk of bias in this domain.

D5 — Bias in selection of the reported result​

R1 (Some concerns): The document does not mention prospective trial registration (e.g., in a clinical trials registry), raising concerns about selective reporting.

R2 (Low risk): All outcomes described in the methods (SCL-90 and POMS subscales) are fully reported, including significant and non-significant results (e.g., no change in "psychoticism" or "self-esteem" scores).

R1 (Some concerns): Lack of prospective registration introduces uncertainty, though comprehensive reporting aligns with methods.

Consensus: Some concerns​

Overall (Consensus): Some concerns​

Rationale: All domains (D1–D5) are rated "Some concerns." Key issues include inadequate randomization details, lack of blinding, subjective outcomes, and no prospective registration. However, the absence of attrition and balanced baseline characteristics partially mitigate risk. The study's limitations warrant cautious interpretation of the results.

**4.ROB2 Assessment for Ma et al., 2016**

D1 — Bias arising from the randomization process​

R1 (Some concerns): The document states participants were "randomly divided" into exercise and control groups but provides no description of the random sequence generation method (e.g., computer-generated, random number table).

R2 (Some concerns): No information is provided on allocation concealment (e.g., sealed envelopes or central randomization). The absence of safeguards introduces selection bias risk.

Consensus: Some concerns​

Rationale: Lack of details on randomization method and allocation concealment introduces uncertainty, though baseline balance mitigates risk.

D2 — Bias due to deviations from the intended interventions​

R1 (Some concerns): The intervention (Mawangdui Daoyinshu) is behavioral, and blinding of participants was not feasible. However, exercise intensity was monitored (Polar heart rate watches, 60%–80% max heart rate).

R2 (High risk): The primary outcomes (POMS and SAS scores) are subjective self-reported measures. The document does not mention whether outcome assessors were blinded.

R3 (High risk): Lack of participant blinding combined with subjective outcomes and unclear assessor blinding raises significant concerns about performance and detection bias.

Consensus: High risk​

Rationale: Subjective outcomes without blinding measures dominate the risk of bias in this domain.

D3 — Bias due to missing outcome data​

R1 (Low risk): Attrition was low (2/40 dropouts in exercise group, 5%; 0/40 in control group). Reasons included personal factors.

R2 (Some concerns): No intention-to-treat (ITT) analysis or methods for handling missing data are described. Analyses appear per-protocol.

R3 (Some concerns): Although attrition is low, the lack of ITT analysis introduces uncertainty.

Consensus: Some concerns​

Rationale: Low attrition supports lower risk, but absence of ITT analysis warrants caution.

D4 — Bias in measurement of the outcome​

R1 (Some concerns): Primary outcomes (POMS and SAS scores) are subjective self-reported measures. Although scales are validated (POMS reliability 0.71), subjectivity increases measurement bias risk.

R2 (Some concerns): No details are provided on whether assessors were trained to standardize assessments or if protocols were consistently applied.

Consensus: Some concerns​

Rationale: Subjective outcomes without clear assessment protocols or blinding introduce measurement bias risk.

D5 — Bias in selection of the reported result​

R1 (Some concerns): The document mentions a funding source but does not indicate prospective trial registration.

R2 (Low risk): All pre-specified outcomes (POMS subscales, SAS scores) are fully reported, including statistical results.

R3 (Some concerns): Lack of prospective registration introduces uncertainty, but comprehensive reporting aligns with methods.

Consensus: Some concerns​

Overall (Consensus): High risk​

Rationale: Domain D2 is rated "High risk" due to subjective outcomes without blinding measures. Additionally, D1, D3, D4, and D5 show "Some concerns" due to randomization limitations, missing data handling, subjective measurements, and no prospective registration. The reliance on subjective outcomes without blinding fundamentally undermines the validity of the results. The study's limitations necessitate cautious interpretation.

**5.ROB2 Assessment for Mo et al., 2016**

D1 — Bias arising from the randomization process​

R1 (Some concerns): The document states participants were (randomly divided into exercise and control groups) but provides no description of the random sequence generation method (e.g., computer-generated, random number table).

R2 (High risk): No information is provided on allocation concealment (e.g., sealed envelopes or central randomization). The absence of safeguards introduces significant selection bias risk.

R3 (Low risk): Baseline characteristics (age, height, weight) show no significant differences between groups (e.g., age: exercise group 65.7±5.1 vs. control group 66.4±4.9, p>0.05), supporting baseline comparability.

Consensus: Some concerns​

Rationale: The lack of randomization methodology details and complete absence of allocation concealment introduce uncertainty, though baseline balance partially mitigates risk.

D2 — Bias due to deviations from the intended interventions​

R1 (High risk): The intervention (Tai Ji Yang Sheng Zhang) is behavioral, and blinding of participants was not feasible. The exercise was supervised, but the behavioral nature introduces high risk of performance bias.

R2 (High risk): Primary outcomes (POMS and SCL-90 scores) are subjective self-reported measures. The document does not mention whether outcome assessors were blinded.

Consensus: High risk​

Rationale: Subjective outcomes without blinding measures dominate the risk of bias in this domain.

D3 — Bias due to missing outcome data​

R1 (Low risk): Attrition was low (1/40 dropouts in exercise group, 2.5%; 0/40 in control group). The overall attrition rate was 1.25%.

R2 (Some concerns): No intention-to-treat (ITT) analysis or specific methods for handling missing data are described. Analyses appear to be per-protocol.

R3 (Low risk): The very low attrition rate (<5%) minimizes potential bias impact.

Consensus: Some concerns​

Rationale: While attrition is minimal, the lack of ITT analysis introduces theoretical uncertainty.

D4 — Bias in measurement of the outcome​

R1 (Some concerns): Primary outcomes (POMS and SCL-90 scores) are subjective self-reported measures. Although scales are validated, their subjectivity increases susceptibility to measurement bias.

R2 (Some concerns): No information is provided on assessor training or standardization of assessment procedures. The document mentions "指导监控" (guidance and monitoring) but lacks methodological details.

Consensus: Some concerns​

Rationale: Subjective measurement tools without robust blinding protocols introduce measurement bias risk.

D5 — Bias in selection of the reported result​

R1 (High risk): The document does not mention prospective trial registration, and no protocol is referenced.

R2 (Low risk): All outcomes described in the methods (POMS subscales, SCL-90 dimensions) are fully reported with statistical results.

Consensus: Some concerns​

Rationale: Lack of prospective registration introduces significant uncertainty about selective reporting.

Overall (Consensus): High risk​

Rationale: Domain D2 is rated "High risk" due to subjective outcomes without blinding measures. Additionally, D1, D3, D4, and D5 show concerns related to randomization, missing data handling, subjective measurements, and trial registration. The reliance on subjective outcomes combined with inadequate blinding and methodological reporting limitations fundamentally undermines the study's validity. The results should be interpreted with caution due to these significant methodological limitations.

**6.ROB2 Assessment for Xu et al., 2025**

D1 — Bias arising from the randomization process​

R1 (Low risk): The document explicitly describes the random sequence generation method: (divided into two groups using a random number table method), indicating a standardized and reproducible process.

R2 (Some concerns): Although the sequence generation is clear, the document lacks a detailed description of allocation concealment (e.g., use of sealed envelopes or central randomization). It only states the randomization method without specifying how allocation was concealed.

R3 (Low risk): The baseline characteristics (age, cancer stage) show no significant differences between groups (e.g., age: observation group 53.67±7.45 vs. control group 55.21±6.71, P>0.05), supporting the effectiveness of randomization.

Consensus: Some concerns​

Rationale: The absence of allocation concealment details introduces uncertainty, but the baseline balance and clear sequence generation mitigate the risk. Overall, some concerns remain, but it is not high risk.

D2 — Bias due to deviations from the intended interventions​

R1 (Some concerns): The intervention (ORTCC management combined with Baduanjin) is behavioral, and blinding of participants was not feasible, potentially affecting adherence. However, the intervention was supervised by a multidisciplinary team with standardized protocols.

R2 (Low risk): The document states that (researchers responsible for data collection, entry, and statistical analysis were blinded to group allocation), reducing measurement bias.

R3 (Some concerns): Lack of participant blinding may introduce behavioral bias, but the objective outcomes (e.g., shoulder ROM) and assessor blinding alleviate the risk. Concerns persist due to subjective outcomes (e.g., DASH, HADS).

Consensus: Some concerns​

Rationale: The inability to blind participants and the presence of subjective outcomes raise concerns, though assessor blinding and objective measures mitigate some risk.

D3 — Bias due to missing outcome data​

R1 (Low risk): The document reports no dropouts (100 patients completed the study), indicating 0% attrition.

R2 (Some concerns): No intention-to-treat (ITT) analysis or specific methods for handling missing data are described, though the absence of attrition reduces concern.

R3 (Low risk): With no missing data, the risk of attrition bias is minimal.

Consensus: Some concerns​

Rationale: While no attrition supports low risk, the lack of explicit ITT analysis introduces theoretical uncertainty.

D4 — Bias in measurement of the outcome​

R1 (Some concerns): Primary outcomes include subjective measures (DASH, FACT-B, HADS) and objective measures (shoulder ROM). The subjective outcomes are patient-reported and may be influenced by lack of participant blinding.

R2 (Low risk): Objective outcomes (shoulder ROM) were measured using standardized tools (goniometer), and assessors were blinded, reducing measurement bias.

R3 (Some concerns): The combination of subjective and objective outcomes introduces mixed risk, but the blinding of assessors for objective measures supports lower risk.

Consensus: Low risk​

Rationale: The objectivity of shoulder ROM measurements and assessor blinding ensure low measurement bias risk for key outcomes.

D5 — Bias in selection of the reported result​

R1 (Some concerns): The document mentions funding sources but does not indicate prospective trial registration (e.g., in a clinical trials registry).

R2 (Low risk): All pre-specified outcomes (DASH, FACT-B, HADS, shoulder ROM) are fully reported, including statistical results and non-significant baseline comparisons.

R3 (Some concerns): Lack of prospective registration introduces uncertainty, but comprehensive outcome reporting aligns with methods.

Consensus: Some concerns​

Overall (Consensus): Some concerns​

Rationale: The worst-rated domains are D1, D2, D3, and D5 ("Some concerns"). Key limitations include insufficient allocation concealment details (D1), inability to blind participants (D2), no ITT analysis (D3), and no prospective registration (D5). However, the low risk in outcome measurement (D4) due to objective measures and assessor blinding, along with no attrition, supports the study's credibility. The biases do not invalidate the results but warrant cautious interpretation.

**7.ROB2 Assessment for Yang et al., 2023**

D1 — Bias arising from the randomization process​

R1 (Low risk): The document explicitly describes the random sequence generation method: (divided into experimental and control groups using a random number table method), indicating a standardized process.

R2 (Some concerns): No information is provided on allocation concealment (e.g., sealed envelopes or central randomization). The absence of allocation concealment mechanisms introduces potential selection bias.

R3 (Low risk): Baseline characteristics show no significant differences in age between groups (experimental group 47.88±2.49 vs. control group 48.75±2.41, P>0.05), supporting successful randomization.

Consensus: Some concerns​

Rationale: While random sequence generation is clearly described and baseline characteristics are balanced, the lack of allocation concealment details introduces uncertainty.

D2 — Bias due to deviations from the intended interventions​

R1 (Some concerns): The intervention (Baduanjin exercise) is behavioral, making participant blinding impossible. However, the exercise was conducted with professional supervision.

R2 (Some concerns): The document does not mention whether outcome assessors were blinded. While pulmonary function tests are objective, psychological assessments (SAS, SDS) are subjective and could be influenced by assessor knowledge.

Consensus: Some concerns​

Rationale: The behavioral nature of the intervention and lack of blinding details introduce potential bias, particularly for subjective psychological outcomes.

D3 — Bias due to missing outcome data​

R1 (Low risk): The document reports no dropouts (80 patients completed the study), indicating 0% attrition.

R2 (Some concerns): No intention-to-treat (ITT) analysis or specific methods for handling missing data are described, though the absence of attrition reduces concern.

R3 (Low risk): With no missing data, the risk of attrition bias is minimal.

Consensus: Some concerns​

Rationale: While no attrition supports low risk, the lack of explicit ITT analysis introduces theoretical uncertainty.

D4 — Bias in measurement of the outcome​

R1 (Low risk): Primary outcomes include objective measures (pulmonary function tests: FEV1, FEV1%, FVC) using standardized equipment and protocols.

R2 (Some concerns): Secondary outcomes include subjective measures (SAS, SDS scores) which are patient-reported and could be influenced by the lack of blinding.

R3 (Low risk): The objectivity of primary pulmonary outcomes and use of standardized protocols ensure low measurement bias risk for key outcomes.

Consensus: Low risk​

Rationale: Objective pulmonary function measurements provide strong protection against measurement bias, though subjective psychological measures introduce some concern.

D5 — Bias in selection of the reported result​

R1 (High risk): The document does not mention prospective trial registration in a clinical trials registry.

R2 (Low risk): All pre-specified outcomes (pulmonary function, 6MWD, CAT, SAS, SDS) are fully reported with statistical results.

R3 (Some concerns):Lack of prospective registration introduces significant uncertainty about selective reporting, though comprehensive outcome reporting is evident.

Consensus: Some concerns​

Overall (Consensus): Some concerns​

Rationale: Domains D1, D2, D3, and D5 are rated "Some concerns." Key limitations include inadequate allocation concealment details, lack of blinding information, no ITT analysis, and no prospective registration. However, the low risk in outcome measurement (D4) due to objective pulmonary function tests, along with no attrition and balanced baseline characteristics, supports the credibility of the primary findings. The study's limitations warrant cautious interpretation but do not invalidate the results.

**8.ROB2 Assessment for Ma et al., 2010**

D1 — Bias arising from the randomization process​

R1 (Some concerns): The document states that participants were (randomly divided into experimental and control groups) but provides no description of the random sequence generation method (e.g., computer-generated, random number table). The lack of details introduces uncertainty.

R2 (Some concerns): No information is provided on allocation concealment (e.g., use of sealed envelopes or central randomization). The absence of safeguards against selection bias raises concerns.

R3 (Low risk): Baseline characteristics (age) show no significant differences between groups (experimental group 49.08±2.76 vs. control group 49.30±2.59, P>0.05), and baseline Kupperman and CES-D scores appear similar (Table 1), supporting baseline comparability.

Consensus: Some concerns​

Rationale: The lack of details on random sequence generation and allocation concealment introduces uncertainty, though baseline balance mitigates some risk. Overall, some concerns remain due to inadequate randomization reporting.

D2 — Bias due to deviations from the intended interventions​

R1 (Some concerns): The intervention (Baduanjin exercise) is behavioral, and blinding of participants was not feasible, potentially affecting adherence. However, the exercise was supervised by instructors with standardized protocols.

R2 (Some concerns): The primary outcomes (Kupperman score and CES-D score) are subjective patient-reported measures. The document does not mention whether outcome assessors were blinded, which may introduce detection bias.

Consensus: Some concerns​

Rationale: The behavioral nature of the intervention, combined with subjective outcomes and lack of blinding details, raises concerns about performance and detection bias.

D3 — Bias due to missing outcome data​

R1 (Low risk): The document reports no dropouts or missing data (100 participants completed the study), indicating 0% attrition.

R2 (Some concerns): No intention-to-treat (ITT) analysis or methods for handling missing data are described. Statistical analyses (t-tests) appear per-protocol.

R3 (Low risk): With no missing data, the risk of attrition bias is minimal.

Consensus: Some concerns​

Rationale: While the absence of attrition supports low risk, the lack of ITT analysis or explicit missing data methods introduces theoretical uncertainty.

D4 — Bias in measurement of the outcome​

R1 (Some concerns): The primary outcomes (Kupperman score and CES-D score) are subjective measures based on patient-reported scales. Although the scales are validated, their subjectivity increases susceptibility to measurement bias.

R2 (Some concerns): No information is provided on whether assessors were trained to standardize assessments or if blinding was implemented. The document does not mention procedures to minimize observer bias.

Consensus: Some concerns​

Rationale: The reliance on subjective outcomes without robust blinding or assessment protocols introduces measurement bias concerns.

D5 — Bias in selection of the reported result​

R1 (Some concerns): The document mentions funding (Tangshan healthcare research project 09130202A-3-5) but does not indicate prospective trial registration (e.g., in a clinical trials registry).

R2 (Low risk): All pre-specified outcomes (Kupperman score and CES-D score) are fully reported in the results, including statistical comparisons and P-values.

Consensus: Some concerns​

Rationale: Lack of prospective registration introduces uncertainty about selective reporting, though comprehensive outcome reporting aligns with the methods.

Overall (Consensus): Some concerns​

Rationale: The worst-rated domains are D1, D2, D3, D4, and D5 (all "Some concerns"). Key limitations include inadequate randomization details (D1), lack of blinding for subjective outcomes (D2 and D4), no ITT analysis (D3), and no prospective registration (D5). However, the absence of attrition and baseline balance mitigate some risks. The study's design has methodological weaknesses that warrant cautious interpretation, but the biases do not invalidate the results entirely.

**9.ROB2 Assessment for Shan et al., 2025**

D1 — Bias arising from the randomization process​

R1 (Low risk): The document explicitly describes the random sequence generation method: (patients were divided using a random number table method), indicating a standardized and reproducible process.

R2 (Some concerns): Although the sequence generation is clear, the document lacks detailed description of allocation concealment (e.g., use of sealed envelopes or central randomization). It only states the randomization method without specifying how allocation was concealed.

R3 (Low risk): Baseline characteristics show no significant differences between groups in age, disease duration, and baseline scores (e.g., age: control group 50.63±3.17 vs. study group 50.51±3.08, P>0.05), supporting the effectiveness of randomization.

Consensus: Some concerns​

Rationale: The absence of allocation concealment details introduces uncertainty, but the baseline balance and clear sequence generation mitigate the risk. Overall, some concerns remain, but it is not high risk.

D2 — Bias due to deviations from the intended interventions​

R1 (High risk): The interventions (Baduanjin exercise and Du Mai fumigation) are behavioral, and blinding of participants was not feasible, potentially affecting adherence and introducing performance bias.

R2 (High risk): The primary outcomes (PSQI, SAS, SDS, Kupperman, and SF-36 scores) are subjective patient-reported measures. The document does not mention whether outcome assessors were blinded, raising concerns about detection bias.

Consensus: High risk​

Rationale: The inability to blind participants and unclear assessor blinding for subjective outcomes dominate the risk of bias in this domain.

D3 — Bias due to missing outcome data​

R1 (Low risk): The document reports no dropouts (80 patients completed the study), indicating 0% attrition.

R2 (Some concerns): No intention-to-treat (ITT) analysis or specific methods for handling missing data are described, though the absence of attrition reduces concern.

R3 (Low risk): With no missing data, the risk of attrition bias is minimal.

Consensus: Some concerns​

Rationale: While no attrition supports low risk, the lack of explicit ITT analysis introduces theoretical uncertainty.

D4 — Bias in measurement of the outcome​

R1 (Some concerns): All outcomes are subjective measures (PSQI, SAS, SDS, Kupperman, and SF-36 scores). Although these are validated scales, their subjectivity increases susceptibility to measurement bias.

R2 (Some concerns): No information is provided on assessor training, standardization of assessment procedures, or blinding of assessors.

Consensus: Some concerns​

Rationale: Subjective measurement tools without robust blinding protocols introduce measurement bias risk.

D5 — Bias in selection of the reported result​

R1 (Some concerns): The document mentions funding (Zhuzhou Social Funding Project) but does not indicate prospective trial registration (e.g., in a clinical trials registry).

R2 (Low risk): All pre-specified outcomes (clinical efficacy, PSQI, SAS, SDS, Kupperman, SF-36) are fully reported, including statistical results and between-group comparisons.

R3 (Some concerns): Lack of prospective registration introduces uncertainty, but comprehensive outcome reporting aligns with methods.

Consensus: Some concerns​

Overall (Consensus): High risk​

Rationale: Domain D2 is rated "High risk" due to subjective outcomes without blinding measures. Additionally, D1, D3, D4, and D5 show "Some concerns" related to randomization, missing data handling, subjective measurements, and trial registration. The reliance on subjective outcomes combined with inadequate blinding and methodological reporting limitations fundamentally undermines the study's validity. The results should be interpreted with caution due to these significant methodological limitations.

**10.ROB2 Assessment for Luo et al., 2021**

D1 — Bias arising from the randomization process

R1 (Some concerns): The document states that participants were (randomly divided into two groups) but provides no description of the random sequence generation method (e.g., computer-generated, random number table).

R2 (High risk): No information is provided on allocation concealment (e.g., sealed envelopes or central randomization). The absence of safeguards introduces significant selection bias risk.

R3 (Low risk): Baseline characteristics show no significant differences between groups in age and tumor stage (e.g., age: control group 48.5±3.8 vs. observation group 49.2±3.2, P>0.05), supporting baseline comparability.

Consensus: Some concerns

Rationale: The lack of details on random sequence generation and complete absence of allocation concealment introduce uncertainty, though baseline balance partially mitigates risk.

D2 — Bias due to deviations from the intended interventions

R1 (High risk): The interventions (Baduanjin and Wuxing music) are behavioral, and blinding of participants was not feasible. The exercises were supervised, but the behavioral nature introduces high risk of performance bias.

R2 (High risk): Primary outcomes (SAS, SDS scores) are subjective patient-reported measures. The document does not mention whether outcome assessors were blinded.

Consensus: High risk

Rationale: Subjective outcomes without blinding measures dominate the risk of bias in this domain.

D3 — Bias due to missing outcome data

R1 (Low risk): The document reports no dropouts (70 patients completed the study), indicating 0% attrition.

R2 (Some concerns): No intention-to-treat (ITT) analysis or specific methods for handling missing data are described, though the absence of attrition reduces concern.

R3 (Low risk): With no missing data, the risk of attrition bias is minimal.

Consensus: Some concerns

Rationale: While no attrition supports low risk, the lack of explicit ITT analysis introduces theoretical uncertainty.

D4 — Bias in measurement of the outcome

R1 (High risk): Primary outcomes (SAS and SDS scores) are subjective self-reported measures. Although the scales are validated, their subjectivity increases susceptibility to measurement bias.

R2 (High risk): No information is provided on assessor training, standardization of assessment procedures, or blinding of assessors.

Consensus: High risk

Rationale: Subjective measurement tools without robust blinding protocols introduce significant measurement bias risk.

D5 — Bias in selection of the reported result

R1 (Some concerns): The document mentions funding (Lou Cai Jiao Zhi[2019]) but does not indicate prospective trial registration in a clinical trials registry.

R2 (Low risk): All outcomes described in the methods (SAS, SDS, quality of life scores) are fully reported with statistical results.

R3 (Some concerns): Lack of prospective registration introduces uncertainty, but comprehensive outcome reporting aligns with methods.

Consensus: Some concerns

Overall (Consensus): High risk

Rationale: Domains D2 and D4 are rated "High risk" due to subjective outcomes without blinding measures. Additionally, D1, D3, and D5 show "Some concerns" related to randomization limitations, missing data handling, and trial registration. The reliance on subjective outcomes combined with inadequate blinding and methodological reporting limitations fundamentally undermines the study's validity. The results should be interpreted with extreme caution due to these significant methodological limitations.

**11.ROB2 Assessment for Ma et al., 2010**

D1 — Bias arising from the randomization process​

R1 (Some concerns): The document states that participants were (randomly divided into experimental and control groups) but provides no description of the random sequence generation method (e.g., computer-generated, random number table). The lack of details introduces uncertainty.

R2 (Some concerns): No information is provided on allocation concealment (e.g., use of sealed envelopes or central randomization). The absence of safeguards against selection bias raises concerns.

R3 (Low risk): Baseline characteristics (age) show no significant differences between groups (experimental group 49.08±2.76 vs. control group 49.30±2.59, P>0.05), and baseline Kupperman and CES-D scores appear similar (Table 1), supporting baseline comparability.

Consensus: Some concerns​

Rationale: The lack of details on random sequence generation and allocation concealment introduces uncertainty, though baseline balance mitigates some risk. Overall, some concerns remain due to inadequate randomization reporting.

D2 — Bias due to deviations from the intended interventions​

R1 (Some concerns): The intervention (Baduanjin exercise) is behavioral, and blinding of participants was not feasible, potentially affecting adherence. However, the exercise was supervised by instructors with standardized protocols.

R2 (Some concerns): The primary outcomes (Kupperman score and CES-D score) are subjective patient-reported measures. The document does not mention whether outcome assessors were blinded, which may introduce detection bias.

Consensus: Some concerns​

Rationale: The behavioral nature of the intervention, combined with subjective outcomes and lack of blinding details, raises concerns about performance and detection bias.

D3 — Bias due to missing outcome data​

R1 (Low risk): The document reports no dropouts or missing data (100 participants completed the study), indicating 0% attrition.

R2 (Some concerns): No intention-to-treat (ITT) analysis or methods for handling missing data are described. Statistical analyses (t-tests) appear per-protocol.

R3 (Low risk): With no missing data, the risk of attrition bias is minimal.

Consensus: Some concerns​

Rationale: While the absence of attrition supports low risk, the lack of ITT analysis or explicit missing data methods introduces theoretical uncertainty.

D4 — Bias in measurement of the outcome​

R1 (Some concerns): The primary outcomes (Kupperman score and CES-D score) are subjective measures based on patient-reported scales. Although the scales are validated, their subjectivity increases susceptibility to measurement bias.

R2 (Some concerns): No information is provided on whether assessors were trained to standardize assessments or if blinding was implemented. The document does not mention procedures to minimize observer bias.

Consensus: Some concerns​

Rationale: The reliance on subjective outcomes without robust blinding or assessment protocols introduces measurement bias concerns.

D5 — Bias in selection of the reported result​

R1 (Some concerns): The document mentions funding (Tangshan healthcare research project 09130202A-3-5) but does not indicate prospective trial registration (e.g., in a clinical trials registry).

R2 (Low risk): All pre-specified outcomes (Kupperman score and CES-D score) are fully reported in the results, including statistical comparisons and P-values.

Consensus: Some concerns​

Rationale: Lack of prospective registration introduces uncertainty about selective reporting, though comprehensive outcome reporting aligns with the methods.

Overall (Consensus): Some concerns​

Rationale: The worst-rated domains are D1, D2, D3, D4, and D5 (all "Some concerns"). Key limitations include inadequate randomization details (D1), lack of blinding for subjective outcomes (D2 and D4), no ITT analysis (D3), and no prospective registration (D5). However, the absence of attrition and baseline balance mitigate some risks. The study's design has methodological weaknesses that warrant cautious interpretation, but the biases do not invalidate the results entirely.

**12.ROB2 Assessment for Wen et al., 2024**

D1 — Bias arising from the randomization process

R1 (Low risk): The document explicitly describes the random sequence generation method: (patients were divided into control and study groups using a random number table method), indicating a standardized and reproducible process.

R2 (Some concerns): Although the sequence generation is clear, the document lacks detailed description of allocation concealment (e.g., use of sealed envelopes or central randomization). No information is provided on how the allocation sequence was concealed.

R3 (Low risk): Baseline characteristics show no significant differences between groups in age, BMI, clinical TNM staging, education level, tumor location, or surgical incision type (e.g., age: control group 45.19±5.37 vs. study group 45.38±4.91, P>0.05), supporting the effectiveness of randomization.

Consensus: Some concerns

Rationale: The absence of allocation concealment details introduces uncertainty, but the baseline balance and clear sequence generation mitigate the risk. Overall, some concerns remain, but it is not high risk.

D2 — Bias due to deviations from the intended interventions

R1 (Some concerns): The interventions (early rehabilitation training based on ERAS concept and Baduanjin exercises) are behavioral, and blinding of participants was not feasible, potentially affecting adherence. However, the exercises were supervised with standardized protocols.

R2 (Some concerns): The document does not explicitly mention whether outcome assessors were blinded. While some outcomes are objective (e.g., shoulder joint mobility), others are subjective (SAS, SDS, VAS scores) and may be influenced by lack of assessor blinding.

Consensus: Some concerns

Rationale: The inability to blind participants and unclear assessor blinding for subjective outcomes introduce performance and detection bias risks.

D3 — Bias due to missing outcome data

R1 (Low risk): The document reports no dropouts (128 patients completed the study), indicating 0% attrition.

R2 (Some concerns): No intention-to-treat (ITT) analysis or specific methods for handling missing data are described, though the absence of attrition reduces concern.

R3 (Low risk): With no missing data, the risk of attrition bias is minimal.

Consensus: Some concerns

Rationale: While no attrition supports low risk, the lack of explicit ITT analysis introduces theoretical uncertainty.

D4 — Bias in measurement of the outcome

R1 (Low risk): Some outcomes are objective measures (shoulder joint mobility measured with goniometer, Constant-Murley shoulder function score) using standardized tools and protocols.

R2 (Some concerns): Other outcomes (SAS, SDS, VAS scores, SF-36 scores) are subjective patient-reported measures that may be influenced by lack of blinding.

R3 (Some concerns): The combination of objective and subjective measurements results in mixed risk, with subjective outcomes dominating the concern.

Consensus: Some concerns

Rationale: While objective measurements provide some protection, the substantial number of subjective outcomes without blinding details introduces measurement bias risk.

D5 — Bias in selection of the reported result

R1 (Some concerns): The document mentions funding (Hunan Provincial Health Commission research project 202204135364) but does not indicate prospective trial registration in a clinical trials registry.

R2 (Low risk): All pre-specified outcomes (SDS, SAS, SF-36, Constant-Murley, VAS, shoulder mobility) are fully reported with statistical results, including between-group comparisons.

R3 (Some concerns): Lack of prospective registration introduces uncertainty, but comprehensive outcome reporting aligns with methods.

Consensus: Some concerns

Overall (Consensus): Some concerns

Rationale: All domains (D1-D5) are rated "Some concerns." Key limitations include insufficient allocation concealment details (D1), lack of blinding information for subjective outcomes (D2 and D4), no ITT analysis (D3), and no prospective registration (D5). However, the absence of attrition, baseline balance, and use of some objective measurements provide partial mitigation. The study's limitations warrant cautious interpretation but do not invalidate the results for the primary outcomes.

**13.ROB2 Assessment for Wei et al., 2021**

D1 — Bias arising from the randomization process​

R1 (Some concerns): The document states that participants were "randomly assigned... using SPSS 24.0 software" but provides no detailed description of the random sequence generation method (e.g., computer-generated, random number table). The lack of specifics (e.g., block size or seed values) introduces uncertainty.

R2 (High risk): No information is provided on allocation concealment (e.g., sealed envelopes or central randomization). The absence of safeguards introduces significant selection bias risk.

R3 (Low risk): Baseline characteristics (Table 1) show no significant differences between groups in age, BMI, education, menopausal status, or clinical factors (e.g., age: intervention group 55.4±5.6 vs. control group 56.2±5.3, P>0.05), supporting baseline comparability.

Consensus: Some concerns​

Rationale: The absence of details on random sequence generation and complete lack of allocation concealment introduce uncertainty, though baseline balance partially mitigates risk. Overall, some concerns remain due to inadequate reporting.

D2 — Bias due to deviations from the intended interventions​

R1 (Some concerns): The intervention (Baduanjin exercise) is behavioral, and blinding of participants was not feasible, potentially affecting adherence. However, exercises were supervised with standardized protocols (5 sessions/week, 30 min/session for 12 weeks) and monitored via exercise logs.

R2 (Some concerns): The document does not mention whether outcome assessors were blinded. Primary outcomes (e.g., FACT-Cog, fatigue, anxiety) are subjective patient-reported measures, and lack of assessor blinding may introduce detection bias.

Consensus: Some concerns​

Rationale: The inability to blind participants and unclear assessor blinding for subjective outcomes introduce performance and detection bias risks.

D3 — Bias due to missing outcome data​

R1 (Low risk): The document reports no dropouts (70 participants completed the study), indicating 0% attrition.

R2 (Some concerns): The document states the use of intention-to-treat (ITT) analysis but provides no specific methods for handling missing data (e.g., multiple imputation). Analyses were performed using linear mixed-effects models.

R3 (Low risk): With no missing data, the risk of attrition bias is minimal.

Consensus: Some concerns​

Rationale: While no attrition supports low risk, the lack of detailed ITT methods introduces theoretical uncertainty. Overall, the risk is manageable.

D4 — Bias in measurement of the outcome​

R1 (Some concerns): Primary outcomes (FACT-Cog, fatigue via MFSI-SF, anxiety/depression via HADS) are subjective patient-reported measures. Although scales are validated, their subjectivity increases susceptibility to measurement bias.

R2 (Some concerns): No information is provided on assessor training, standardization of assessment procedures, or blinding of assessors. The document mentions data collection by "trained research assistants" but lacks details.

Consensus: Some concerns​

Rationale: Subjective measurement tools without robust blinding protocols introduce measurement bias risk.

D5 — Bias in selection of the reported result​

R1 (Low risk): The trial was prospectively registered in the Chinese Clinical Trial Registry (ChiCTR 2000033152), with registration prior to participant enrollment. The reported outcomes align with the registry scope.

R2 (Low risk): All pre-specified outcomes (cognitive function, fatigue, anxiety, depression, quality of life) are fully reported in tables and text, including non-significant results and longitudinal data (T0–T3).

Consensus: Low risk​

Rationale: Prospective registration and comprehensive outcome reporting indicate low risk of selective reporting.

Overall (Consensus): Some concerns​

Rationale: Domains D1, D2, D3, and D4 are rated "Some concerns." Key limitations include inadequate randomization details, lack of blinding information, vague ITT methods, and subjective outcomes without assessor blinding. However, the low risk in selective reporting (D5), absence of attrition, and baseline balance support the study's credibility. The use of ITT analysis and longitudinal mixed-effects models partially mitigates biases. The results should be interpreted with caution due to these limitations, but the biases do not invalidate the primary findings.

**14.ROB2 Assessment for Larkey et al., 2014**

D1 — Bias arising from the randomization process​

R1 (Some concerns): The document describes the study as a "double-blind, randomized controlled trial" but provides no description of the random sequence generation method (e.g., computer-generated, random number table). The lack of methodological details introduces uncertainty.

R2 (High risk): No information is provided on allocation concealment (e.g., use of sealed envelopes or central randomization). The absence of safeguards introduces significant selection bias risk.

R3 (Low risk): Baseline characteristics (Table 1) show no significant differences between groups in age, BMI, physical activity, or other factors (e.g., age: QG/TCE group comparable to SQG group, P>0.05), supporting baseline comparability.

Consensus: Some concerns​

Rationale: The absence of details on random sequence generation and complete lack of allocation concealment introduce uncertainty, though baseline balance partially mitigates risk. Overall, some concerns remain, but it is not high risk.

D2 — Bias due to deviations from the intended interventions​

R1 (Low risk): The document mentions "double-blind" design, indicating blinding of participants and assessors. Although the intervention (Qigong/Tai Chi Easy) is behavioral, assessor blinding ("assessors were blind to group allocation") reduces measurement bias.

R2 (Some concerns): Primary outcomes (fatigue via FSI, depression via BDI, sleep via PSQI) are subjective patient-reported measures. While blinding is in place, the subjectivity may introduce detection bias.

R3 (Some concerns): Lack of participant blinding for behavioral interventions may introduce performance bias, but assessor blinding and standardized delivery (supervised sessions, home practice logs) alleviate some risk.

Consensus: Some concerns​

Rationale: The subjective nature of outcomes and behavioral intervention introduce performance and detection bias risks, though blinding measures provide partial protection.

D3 — Bias due to missing outcome data​

R1 (Some concerns): Attrition was moderate (101 randomized, 87 completed, 13.9% dropout), with reasons including personal conflicts and low attendance.

R2 (Low risk): The document states use of hierarchical linear models that "allow inclusion of any individuals with missing data," implying intention-to-treat principles, though no specific methods (e.g., multiple imputation) are described.

R3 (Some concerns): The moderate attrition rate and lack of detailed ITT methods introduce slight uncertainty, but the analytical approach supports manageable risk.

Consensus: Some concerns​

Rationale: Attrition >10% warrants concern, but the use of models accommodating missing data mitigates risk. Overall, the risk is manageable.

D4 — Bias in measurement of the outcome​

R1 (Some concerns): Primary outcomes (FSI, BDI, PSQI) are subjective patient-reported measures, though they are validated scales. Blinding of assessors reduces human bias.

R2 (Low risk): Standardized protocols are used (e.g., validated questionnaires, consistent timing), and assessor blinding is maintained.

R3 (Some concerns): The subjectivity of outcomes introduces measurement bias risk, but blinding and standardization ensure low risk for detection bias.

Consensus: Some concerns​

Rationale: Subjective measurements dominate, but blinding and protocols partially mitigate bias risk.

D5 — Bias in selection of the reported result​

R1 (Some concerns): The document mentions IRB approval but does not indicate prospective trial registration (e.g., in a clinical trials registry).

R2 (Low risk): All pre-specified outcomes (fatigue, depression, sleep) are fully reported with statistical results, including non-significant findings and longitudinal data.

R3 (Some concerns): Lack of prospective registration introduces uncertainty, but comprehensive outcome reporting aligns with methods.Consensus: Some concerns​

Overall (Consensus): Some concerns​

Rationale: The worst-rated domains are D1, D2, D3, D4, and D5 ("Some concerns"). Key limitations include inadequate randomization details, subjective outcomes with behavioral interventions, moderate attrition, and no prospective registration. However, the use of blinding, baseline balance, and analytical methods accommodating missing data provide partial mitigation. The study design has strengths, but the biases warrant cautious interpretation of results.

**15.ROB2 Assessment for Chen et al., 2013**

D1 — Bias arising from the randomization process​

R1 (Some concerns): The document describes randomization using "a form of adaptive randomization called minimization" to balance groups by factors like disease stage and age, but provides no detailed description of the random sequence generation method (e.g., computer-generated, random number table). The lack of specifics introduces uncertainty.

R2 (High risk): No information is provided on allocation concealment (e.g., use of sealed envelopes or central randomization). The absence of safeguards introduces significant selection bias risk.

R3 (Low risk): Baseline characteristics (Table 1) show no significant differences between groups in age, marital status, education, income, disease stage, or other factors (e.g., age: qigong group 45.3±6.3 vs. control group 44.7±9.7, P=0.73), supporting the effectiveness of randomization.

Consensus: Some concerns​

Rationale: The absence of details on random sequence generation and complete lack of allocation concealment introduce uncertainty, but the baseline balance partially mitigates risk. Overall, some concerns remain, but it is not high risk.

D2 — Bias due to deviations from the intended interventions​

R1 (Some concerns): The intervention (qigong) is behavioral, and blinding of participants was not feasible, potentially affecting adherence. However, sessions were supervised with standardized protocols (5 weekly classes over 5–6 weeks) and high attendance (65.2% attended ≥80% of sessions).

R2 (Low risk): The document mentions assessor blinding ("assessors were blind to group allocation"), reducing measurement bias. Primary outcomes include both subjective (e.g., depressive symptoms via CES-D) and objective measures (e.g., cortisol), but assessor blinding protects against detection bias.

R3 (Some concerns): Lack of participant blinding may introduce behavioral bias, particularly for subjective outcomes, but the objective components and supervised delivery alleviate some risk.

Consensus: Some concerns​

Rationale: The inability to blind participants introduces performance bias risk, but assessor blinding and standardized protocols mitigate concerns for objective outcomes. Subjective outcomes remain a source of some concern.

D3 — Bias due to missing outcome data​

R1 (Low risk): Attrition was low (100 randomized, 96 completed assessments; one death after T4, but data included until dropout). The completion rate was high (96%).

R2 (Some concerns): The document states analyses used "multilevel modeling analyses" but does not explicitly mention intention-to-treat (ITT) principles or methods for handling missing data (e.g., imputation). Analyses appear to include all available data.

R3 (Low risk): The low attrition rate minimizes the impact of missing data, supporting low risk.

Consensus: Some concerns​

Rationale: While attrition is low, the lack of explicit ITT methods introduces slight uncertainty. Overall, the risk is manageable.

D4 — Bias in measurement of the outcome​

R1 (Low risk): Primary outcomes include objective measures (cortisol levels via saliva assays) with standardized protocols (e.g., calibrated equipment, multiple time points). Assessor blinding further reduces human bias.

R2 (Some concerns): Secondary outcomes (e.g., depressive symptoms, fatigue, QOL via questionnaires like CES-D, BFI, FACT-G) are subjective and patient-reported, which may be influenced by lack of participant blinding.

R3 (Low risk): The objectivity of cortisol measurements and assessor blinding ensure low measurement bias risk for key outcomes; potential bias in subjective outcomes does not affect the main conclusions.

Consensus: Low risk​

Rationale: Objective laboratory measures and blinding of assessors provide strong protection against measurement bias.

D5 — Bias in selection of the reported result​

R1 (High risk): The document mentions IRB approval but does not indicate prospective trial registration (e.g., in a clinical trials registry). The absence of registration raises concerns about selective reporting.

R2 (Low risk): All pre-specified outcomes (depressive symptoms, fatigue, sleep disturbance, QOL, cortisol) are fully reported in tables and text, including non-significant results (e.g., no differences in sleep or cortisol).

R3 (Some concerns): Lack of prospective registration introduces uncertainty, but comprehensive outcome reporting aligns with methods.

Consensus: Some concerns​

Overall (Consensus): Some concerns​

Rationale: The worst-rated domains are D1, D2, D3, and D5 ("Some concerns"). Key limitations include inadequate randomization details (D1), lack of participant blinding (D2), vague handling of missing data (D3), and no prospective registration (D5). However, the low risk in outcome measurement (D4) due to objective assessments and assessor blinding, along with low attrition and baseline balance, supports the credibility of the primary findings. The study's design has strengths, but the biases warrant cautious interpretation of the results.

**16.ROB2 Assessment for Sun et al., 2019**

D1 — Bias arising from the randomization process​

R1 (Low risk): The document explicitly describes the random sequence generation method: (divided into observation and control groups using a random number table method), indicating a standardized and reproducible process.

R2 (Some concerns): Although the sequence generation is clear, the document lacks detailed description of allocation concealment (e.g., use of sealed envelopes or central randomization). No information is provided on how the allocation sequence was concealed.

R3 (Low risk): Baseline characteristics (Table 1) show no significant differences between groups in age, marital status, education level, or pathology stage (e.g., age: observation group 50.96±13.18 vs. control group 49.14±4.09, P>0.05), supporting the effectiveness of randomization.

Consensus: Some concerns​

Rationale: The absence of allocation concealment details introduces uncertainty, but the baseline balance and clear sequence generation mitigate the risk. Overall, some concerns remain, but it is not high risk.

D2 — Bias due to deviations from the intended interventions​

R1 (Some concerns): The interventions (Baduanjin and resting meditation) are behavioral, and blinding of participants was not feasible, potentially affecting adherence. However, exercises were supervised with standardized protocols (e.g., daily practice, 12 min for Baduanjin and 18 min for meditation, over 28 days).

R2 (Some concerns): The document does not mention whether outcome assessors were blinded. Primary outcomes include subjective measures (e.g., SAS, SDS, QLQ-C30 scores) and objective measures (e.g., immune indicators CD3+, CD4+), and lack of assessor blinding may introduce detection bias, particularly for subjective components.

Consensus: Some concerns​

Rationale: The inability to blind participants and unclear assessor blinding introduce performance and detection bias risks, though objective measurements provide partial protection.

D3 — Bias due to missing outcome data​

R1 (Low risk): The missing data rate is low (70 participants randomized, 67 completed, 4.3% attrition due to dropouts: 2 in observation group, 1 in control group).

R2 (Some concerns): Although attrition is low, the document does not mention the use of intention-to-treat (ITT) analysis or specific methods for handling missing data (e.g., multiple imputation). Analyses appear per-protocol.

R3 (Some concerns): The low missing rate supports low risk, but the lack of ITT analysis introduces slight uncertainty. Overall, the risk is manageable.

Consensus: Some concerns​

Rationale: While low attrition reduces bias risk, the absence of ITT methods warrants caution.

D4 — Bias in measurement of the outcome​

R1 (Some concerns): Primary outcomes include subjective patient-reported measures (SAS, SDS, QLQ-C30 scores) and objective laboratory measures (immune indicators via flow cytometry). The subjectivity of some outcomes increases susceptibility to measurement bias.

R2 (Some concerns): The document describes standardized protocols (e.g., flow cytometry with calibrated equipment) but does not mention assessor blinding or training for subjective assessments, raising concerns about human bias.

Consensus: Some concerns​

Rationale: Subjective measurements without robust blinding protocols introduce measurement bias risk, though objective components provide some protection.

D5 — Bias in selection of the reported result​

R1 (Some concerns): The document mentions ethical approval but does not indicate prospective trial registration (e.g., in a clinical trials registry).

R2 (Low risk): All pre-specified outcomes (anxiety, depression, quality of life, immune indicators) are fully reported with statistical results, including non-significant findings (e.g., CD3+ changes not significant).

R3 (Some concerns): Lack of prospective registration introduces uncertainty, but comprehensive outcome reporting aligns with methods.

Consensus: Some concerns​

Overall (Consensus): Some concerns​

Rationale: The worst-rated domains are D1, D2, D3, D4, and D5 ("Some concerns"). Key limitations include insufficient allocation concealment details (D1), lack of blinding information (D2), no ITT analysis (D3), subjective measurements without assessor blinding (D4), and no prospective registration (D5). However, the low attrition rate, baseline balance, and objective components of outcomes provide partial mitigation. The study's design has strengths in randomization sequence reporting and outcome comprehensiveness, but the biases warrant cautious interpretation of the results. The risks do not invalidate the findings but highlight the need for careful consideration.

**17.ROB2 Assessment for Shi et al., 2025**

D1 — Bias arising from the randomization process​

R1 (Low risk): The document explicitly describes the random sequence generation method: (randomly divided into two groups using a random number table method), indicating a standardized and reproducible process.

R2 (Some concerns): Although the sequence generation is clear, the document lacks detailed description of allocation concealment (e.g., use of sealed envelopes or central randomization). No information is provided on how the allocation sequence was concealed.

R3 (Low risk): Baseline characteristics (Table 1) show no significant differences between groups in age, marital status, education, pathology type, clinical stage, and other factors (e.g., age: observation group 56.73±6.88 vs. control group 53.24±5.61, P>0.05), supporting the effectiveness of randomization.

Consensus: Some concerns​

Rationale: The absence of allocation concealment details introduces uncertainty, but the baseline balance and clear sequence generation mitigate the risk. Overall, some concerns remain, but it is not high risk.

D2 — Bias due to deviations from the intended interventions​

R1 (Some concerns): The interventions (multi-track psychological support and Baduanjin exercise) are behavioral, and blinding of participants was not feasible, potentially affecting adherence. However, exercises were supervised with standardized protocols (e.g., Baduanjin: 30 min/session, twice daily; psychological support: group sessions, art therapy).

R2 (Some concerns): The document does not mention whether outcome assessors were blinded. Primary outcomes (SCL-90, GPS, PFS, PSQI, FACT-G) are subjective patient-reported or clinician-assessed measures, and lack of assessor blinding may introduce detection bias.

Consensus: Some concerns​

Rationale: The inability to blind participants and unclear assessor blinding for subjective outcomes introduce performance and detection bias risks.

D3 — Bias due to missing outcome data​

R1 (Low risk): The document reports no dropouts (118 participants completed the study, with final groups of 54 and 64), indicating 0% attrition.

R2 (Some concerns): No intention-to-treat (ITT) analysis or specific methods for handling missing data (e.g., multiple imputation) are described. Analyses appear per-protocol, using completers only.

R3 (Low risk): With no missing data, the risk of attrition bias is minimal.

Consensus: Some concerns​

Rationale: While no attrition supports low risk, the lack of ITT analysis introduces theoretical uncertainty. Overall, the risk is manageable.

D4 — Bias in measurement of the outcome​

R1 (Some concerns): All primary outcomes (SCL-90, GPS, PFS, PSQI, FACT-G) are subjective measures (patient-reported or clinician-assessed scales). Although some scales are validated, their subjectivity increases susceptibility to measurement bias.

R2 (Some concerns): No information is provided on assessor training, standardization of assessment procedures, or blinding of assessors. The document mentions "assessment" but lacks methodological details.

Consensus: Some concerns​

Rationale: Subjective measurement tools without robust blinding protocols introduce measurement bias risk for all outcomes.

D5 — Bias in selection of the reported result​

R1 (Some concerns): The document mentions funding (Xuzhou Medical University Affiliated Hospital Development Fund XYFZ2021008) but does not indicate prospective trial registration (e.g., in a clinical trials registry).

R2 (Low risk): All pre-specified outcomes (SCL-90 subscales, GPS, PFS, PSQI, FACT-G) are fully reported with statistical results, including non-significant findings and between-group comparisons.

R3 (Some concerns): Lack of prospective registration introduces uncertainty, but comprehensive outcome reporting aligns with methods.

Consensus: Some concerns​

Overall (Consensus): Some concerns​

Rationale: All domains (D1–D5) are rated "Some concerns." Key limitations include inadequate allocation concealment details (D1), lack of blinding information for subjective outcomes (D2), no ITT analysis (D3), subjective measurements without assessor blinding (D4), and no prospective registration (D5). However, the low attrition rate, baseline balance, and standardized intervention delivery partially mitigate risks. The study's design has strengths in randomization sequence reporting and outcome comprehensiveness, but the biases warrant cautious interpretation of the results. The risks do not invalidate the findings but highlight the need for careful consideration due to the subjective nature of the outcomes.

**18.ROB2 Assessment for Ni et al., 2021**

D1 — Bias arising from the randomization process​

R1 (Low risk): The document explicitly describes the random sequence generation method: (evenly divided into control and observation groups using a random number table method), indicating a standardized and reproducible process.

R2 (Some concerns): Although the sequence generation is clear, the document lacks detailed description of allocation concealment (e.g., use of sealed envelopes or central randomization). No information is provided on how the allocation sequence was concealed.

R3 (Low risk): Baseline characteristics show no significant differences between groups in age, education level, pathology type, clinical stage, and other factors (e.g., age: observation group 69.61±6.27 vs. control group 69.27±6.55, P>0.05), supporting the effectiveness of randomization.

Consensus: Some concerns​

Rationale: The absence of allocation concealment details introduces uncertainty, but the baseline balance and clear sequence generation mitigate the risk. Overall, some concerns remain, but it is not high risk.

D2 — Bias due to deviations from the intended interventions​

R1 (Some concerns): The interventions (Honghuang decoction and Baduanjin) are behavioral/pharmacological, and blinding of participants was not feasible, potentially affecting adherence. However, interventions were supervised with standardized protocols (Baduanjin: 30 min/session, twice daily; decoction: standardized preparation).

R2 (High risk): The document describes the study as "前瞻性双盲对照研究" (prospective double-blind controlled study) but provides no details on how blinding was implemented or maintained. No information on assessor blinding is provided for subjective outcomes (SAS, SDS, BFI scores).

R3 (Some concerns): Lack of participant blinding may introduce behavioral bias, particularly for subjective outcomes. The document's claim of "double-blind" design without methodological details raises concerns.

Consensus: Some concerns​

Rationale: The inability to blind participants and unclear implementation of blinding introduce performance and detection bias risks.

D3 — Bias due to missing outcome data​

R1 (Low risk): The document reports complete data for all 162 participants (108 randomized + 54 additional chemotherapy-only participants), indicating 0% attrition.

R2 (High risk): No intention-to-treat (ITT) analysis or specific methods for handling missing data are described. Analyses appear per-protocol.

R3 (Low risk): With no missing data, the risk of attrition bias is minimal.

Consensus: Some concerns​

Rationale: While no attrition supports low risk, the lack of ITT analysis introduces theoretical uncertainty. Overall, the risk is manageable.

D4 — Bias in measurement of the outcome​

R1 (High risk): Primary outcomes (SAS, SDS, BFI scores) are subjective patient-reported measures. Although some objective measures (NO, SOD levels) are included, the main outcomes are subjective and vulnerable to measurement bias.

R2 (High risk): No information is provided on assessor training, standardization of assessment procedures, or blinding of assessors. The document mentions "assessment" but lacks methodological details.

Consensus: High risk​

Rationale: Subjective measurement tools without robust blinding protocols introduce significant measurement bias risk.

D5 — Bias in selection of the reported result​

R1 (Some concerns): The document mentions ethical approval and funding but does not indicate prospective trial registration in a clinical trials registry.

R2 (Low risk): All measured outcomes (SAS, SDS, BFI scores, quality of life domains, NO/SOD levels) are fully reported with statistical results, including between-group comparisons and temporal changes.

R3 (Some concerns): Lack of prospective registration introduces uncertainty, but comprehensive outcome reporting aligns with methods.

Consensus: Some concerns​

Overall (Consensus): High risk​

Rationale: Domain D4 is rated "High risk" due to subjective outcomes without proper blinding measures. Domains D1, D2, D3, and D5 show "Some concerns" due to inadequate allocation concealment details, unclear blinding implementation, no ITT analysis, and no prospective registration. The study's methodological limitations, particularly the subjective outcome measurements without assessor blinding, fundamentally undermine the validity of the results. The findings should be interpreted with extreme caution due to these significant bias risks.

**19.ROB2 Assessment for Li et al., 2022**

D1 — Bias arising from the randomization process​

R1 (Some concerns): The document describes randomization as "a 1:1:1 simple randomization technique was employed" and (using a random number table method), but provides no detailed description of the random sequence generation process (e.g., computer-generated, seed values, or specific random number table implementation). The lack of methodological specifics introduces uncertainty.

R2 (High risk): No information is provided on allocation concealment (e.g., use of sealed envelopes or central randomization). The absence of safeguards introduces significant selection bias risk.

R3 (Low risk): Baseline characteristics (Table 1) show no significant differences between groups in age, occupation, BMI, duration of menopause, or other factors (e.g., age: EXD group 56.41±1.68 vs. BE group 57.02±1.64 vs. EXD+BE group 57.31±1.48, P>0.05), supporting baseline comparability.

Consensus: Some concerns​

Rationale: The absence of details on random sequence generation and complete lack of allocation concealment introduce uncertainty, though baseline balance partially mitigates risk. Overall, some concerns remain, but it is not high risk.

D2 — Bias due to deviations from the intended interventions​

R1 (Some concerns): The interventions (Er Xian decoction and Baduanjin exercise) are behavioral/pharmacological, and blinding of participants was not feasible, potentially affecting adherence. However, interventions were supervised with standardized protocols (e.g., BE: 45 min/session, 5 sessions/week; EXD: daily decoction for 16 weeks).

R2 (Some concerns): The document does not mention whether outcome assessors were blinded. While primary outcomes (BMD via DXA) are objective, secondary outcomes (balance tests, psychological scales) involve subjective components, and lack of assessor blinding may introduce detection bias.

Consensus: Some concerns​

Rationale: The inability to blind participants and unclear assessor blinding introduce performance and detection bias risks, though objective measurements provide partial protection.

D3 — Bias due to missing outcome data​

R1 (Low risk): The document reports no dropouts (50 participants completed the study), indicating 0% attrition.

R2 (High risk): No intention-to-treat (ITT) analysis or specific methods for handling missing data are described. Analyses appear per-protocol.

R3 (Low risk): With no missing data, the risk of attrition bias is minimal.

Consensus: Some concerns​

Rationale: While no attrition supports low risk, the lack of ITT analysis introduces theoretical uncertainty. Overall, the risk is manageable.

D4 — Bias in measurement of the outcome​

R1 (Low risk): Primary outcomes (BMD at lumbar spine and femoral neck) are objective measures using DXA scans (Prodigy-GE Healthcare) with standardized protocols, reducing measurement bias.

R2 (Some concerns): Secondary outcomes (e.g., OLST, BBS, TUG, SAS, SDS) include subjective components, and lack of participant blinding may introduce measurement bias (e.g., expectation effects). However, the primary outcomes are the focus and are highly objective.

R3 (Low risk): The objectivity of primary outcomes and standardized protocols ensure low measurement bias risk; potential bias in secondary outcomes does not affect the main conclusions.

Consensus: Low risk​

Rationale: Objective measurement tools and detailed protocols provide strong protection against measurement bias for key outcomes.

D5 — Bias in selection of the reported result​

R1 (Some concerns): The document mentions ethical approval but does not indicate prospective trial registration (e.g., in a clinical trials registry).

R2 (Low risk): All pre-specified outcomes (BMD, balance tests, psychological scales) are fully reported with statistical results, including non-significant findings and longitudinal data (baseline, 8 weeks, 16 weeks).

R3 (Some concerns): Lack of prospective registration introduces uncertainty, but comprehensive outcome reporting aligns with methods.

Consensus: Some concerns​

Overall (Consensus): Some concerns​

Rationale: The worst-rated domains are D1, D2, D3, and D5 ("Some concerns"). Key limitations include inadequate randomization details (D1), lack of blinding information (D2), no ITT analysis (D3), and no prospective registration (D5). However, the low risk in outcome measurement (D4) due to objective BMD assessments, along with no attrition and baseline balance, supports the credibility of the primary findings. The study's design has strengths in standardized interventions and comprehensive reporting, but the biases warrant cautious interpretation of the results. The risks do not invalidate the findings for the objective BMD outcomes.

**20.ROB2 Assessment for Liu et al., 2025**

D1 — Bias arising from the randomization process

R1 (Low risk): The document explicitly describes the random sequence generation method: "Randomization was conducted by independent researchers via an automated permuted block algorithm with a block size of 4." This indicates a standardized and reproducible computerized process.

R2 (Some concerns): The document does not provide a detailed description of allocation concealment (e.g., the use of sequentially numbered, opaque, sealed envelopes or a central randomization system). It only states that participants were "randomly assigned" after baseline assessment. The lack of detail on the mechanism to conceal the allocation sequence introduces some uncertainty.

R3 (Low risk): The baseline characteristics table (Table 1) shows no statistically significant differences between the experimental and sham groups across all measured demographic and clinical variables, supporting the effectiveness of randomization in creating comparable groups.

Consensus: Some concerns

Rationale: The use of a computer-generated permuted block randomization is a strength. However, the absence of specific details regarding allocation concealment introduces some uncertainty about the potential for selection bias. The excellent baseline balance partially mitigates this concern. Overall, some concerns remain due to inadequate reporting of concealment methods.

D2 — Bias due to deviations from the intended interventions

R1 (Some concerns): The interventions include a behavioral component (Tai Chi Chuan) and a device-based neuromodulation (rTMS). Blinding of participants to the Tai Chi component was not feasible. However, for the rTMS component, a sham coil was used, and the document states: "The tai chi chuan coach was unaware of the group allocation and the nature of the intervention." This suggests an attempt to blind the coach.

R2 (Low risk): The document explicitly states: "The outcome evaluators and statistical analysts were masked from group allocation." This protects against detection bias in outcome assessment and analysis.

R3 (Some concerns): Lack of participant blinding to the Tai Chi component may introduce performance bias (e.g., differential expectations, adherence). The use of a validated sham rTMS procedure and the blinding of the Tai Chi coach are mitigating factors. The risk of deviation from the intended protocol is considered low due to the standardized, supervised nature of both interventions.

Consensus: Some concerns

Rationale: The inability to blind participants to the exercise intervention is an inherent limitation, introducing a risk of performance bias. However, the successful blinding of outcome assessors and the use of a sham-controlled rTMS design with a blinded coach significantly reduce the risks of detection bias and ensure intervention fidelity. Overall, some concerns remain regarding performance bias.

D3 — Bias due to missing outcome data

R1 (Low risk): Attrition was low. Of 110 participants randomized, 103 (93.6%) completed the 6-week intervention and 12-week follow-up, indicating a 6.4% dropout rate.

R2 (Low risk): The document explicitly describes rigorous methods for handling missing data: "All participants who provided informed consent were included in the intention-to-treat (ITT) analysis... Multiple imputation was used for missing data on primary outcome measures at baseline, after the intervention, and at follow-up." Both ITT (with multiple imputation) and per-protocol analyses were performed, with consistent results.

Consensus: Low risk

Rationale: Minimal attrition and the application of rigorous statistical methods (ITT with multiple imputation) to handle missing data ensure a low risk of bias in this domain.

D4 — Bias in measurement of the outcome

R1 (Some concerns): The primary outcomes include both subjective patient-reported measures (Pittsburgh Sleep Quality Index, PSQI) and a clinician-administered cognitive assessment (Montreal Cognitive Assessment, MoCA). While the MoCA is standardized, both outcomes involve an element of subjective reporting or assessment.

R2 (Low risk): As noted in D2, outcome assessors were blinded to group allocation. The use of validated scales (PSQI, MoCA) and actigraphy for objective sleep parameters further standardizes measurement.

R3 (Some concerns): The main concern is the subjective nature of the PSQI. Participants were not blinded, which could influence their self-reported sleep scores. However, the blinding of outcome assessors for the MoCA and the use of objective actigraphy data mitigate measurement bias for other endpoints.

Consensus: Some concerns

Rationale: The use of blinded assessors for the MoCA and actigraphy is a strength. For the subjective PSQI, the lack of participant blinding introduces a risk of measurement bias due to expectation effects. This concern is partially offset by the use of a validated instrument and blinded assessors for data collection.

D5 — Bias in selection of the reported result

R1 (Low risk): The trial was prospectively registered on the Chinese Clinical Trial Registry (Identifier: ChiCTR2200063274). A detailed study protocol and statistical analysis plan were also available (Supplement 1).

R2 (Low risk): All pre-specified primary and secondary outcomes mentioned in the methods are fully reported in the results section (Tables 2, 3, 4) for all time points (baseline, 6 weeks, 12 weeks), including non-significant findings (e.g., Trail Making Test Part B at 6 weeks, several actigraphy parameters).

Consensus: Low risk

Rationale: Prospective registration, availability of a protocol, and comprehensive reporting of all outcomes indicate a low risk of selective reporting.

Overall (Consensus): Some concerns

Rationale: Domains D1, D2, and D4 are rated as having Some concerns. The primary limitations are the lack of detailed allocation concealment (D1), the inherent risk of performance bias due to the inability to blind participants to the Tai Chi intervention (D2), and the potential for measurement bias in the self-reported primary sleep outcome (PSQI) due to lack of participant blinding (D4). The study demonstrates significant strengths: computer-generated randomization with good baseline balance, blinded outcome assessment and statistical analysis (D2, D4), low attrition with rigorous ITT and multiple imputation analysis (D3: Low risk), prospective registration, and transparent reporting of all results (D5: Low risk). The overall study design is rigorous for a complex behavioral-neuromodulation trial. While the noted concerns warrant cautious interpretation, particularly for the subjective sleep outcomes, the methodological strengths support the credibility of the main findings regarding the additive benefit of active rTMS over sham rTMS when combined with Tai Chi Chuan.

**21.ROB2 Assessment for Carcelén-Fraile et al., 2022**

D1 — Bias arising from the randomization process​

R1 (Low risk): The document explicitly describes the random sequence generation method: "using a computerized table of numbers" and "participants were randomly assigned... in a 1:1 ratio," indicating a standardized process.

R2 (Some concerns): Although sequence generation is clear, the document lacks detailed description of allocation concealment (e.g., use of sealed envelopes or central randomization). It only mentions that "assignments were kept in sealed opaque envelopes that were opened by an independent party," but without specifics on envelope security or timing.

R3 (Low risk): Baseline characteristics (Table 1) show good balance between groups in age, years since menopause, BMI, occupation, marital status, education, and clinical factors (e.g., age: EG 69.70±6.15 vs. CG 69.75±6.76, P>0.05), supporting randomization effectiveness.

Consensus: Some concerns​

Rationale: The absence of detailed allocation concealment specifics introduces uncertainty, but the baseline balance and clear sequence generation mitigate the risk. Overall, some concerns remain, but it is not high risk.

D2 — Bias due to deviations from the intended interventions​

R1 (Some concerns): The intervention (BaDuanJin Qigong) is behavioral, and blinding of participants was not feasible, potentially affecting adherence. However, the program was supervised with standardized protocols (2 sessions/week, 60 min/session for 12 weeks) by trained instructors.

R2 (Some concerns): The document does not mention whether outcome assessors were blinded. Primary outcomes include subjective measures (PSQI, HADS scores), and lack of assessor blinding may introduce detection bias.

Consensus: Some concerns​

Rationale: The inability to blind participants and unclear assessor blinding for subjective outcomes introduce performance and detection bias risks.

D3 — Bias due to missing outcome data​

R1 (Low risk): The missing data rate is low (125 randomized, 117 completed, 6.4% attrition), with reasons documented (personal conflicts/low attendance).

R2 (Some concerns): Although attrition is low, no intention-to-treat (ITT) analysis or specific methods for handling missing data are described. Analyses appear per-protocol.

R3 (Some concerns): The low missing rate (<10%) supports low risk, but the lack of ITT details introduces slight uncertainty. Overall, the risk is manageable.

Consensus: Some concerns​

Rationale: While low attrition minimizes impact, the absence of explicit ITT methods warrants caution.

D4 — Bias in measurement of the outcome​

R1 (Some concerns): Primary outcomes (PSQI, HADS scores) are subjective patient-reported measures. Although these are validated instruments, their subjectivity increases susceptibility to measurement bias.

R2 (Low risk): The document used standardized assessment tools (PSQI, HADS) with established validity, though no assessor blinding details are provided.

R3 (Some concerns): The subjectivity of outcomes is partially mitigated by validation, but lack of participant blinding for self-reported measures remains a concern.

Consensus: Some concerns​

Rationale: Subjective measurement tools without participant blinding introduce measurement bias risk, though standardized instruments provide some protection.

D5 — Bias in selection of the reported result​

R1 (Low risk): The trial was prospectively registered in ClinicalTrials.gov (NCT03989453), with registration prior to participant enrollment.

R2 (Low risk): All pre-specified outcomes (PSQI components, HADS anxiety/depression) are fully reported with statistical results, including non-significant findings (e.g., no significant effects for sleeping medication or daytime dysfunction).

Consensus: Low risk​

Rationale: Prospective registration and comprehensive outcome reporting indicate low selective reporting risk.

Overall (Consensus): Some concerns​

Rationale: The worst-rated domains are D1, D2, D3, and D4 ("Some concerns"). Limitations include insufficient allocation concealment details (D1), lack of blinding information for subjective outcomes (D2), no ITT analysis (D3), and subjective measurements without participant blinding (D4). However, the low risk in selective reporting (D5), low attrition rate, baseline balance, and prospective registration support the study's credibility. The biases warrant cautious interpretation but do not invalidate the results for the primary outcomes.

**22.ROB2 Assessment for Chang et al., 2024**

D1 — Bias arising from the randomization process​

R1 (Some concerns): The document describes randomization as "divided into four groups through a digital random assignment method" but provides no detailed description of the random sequence generation method (e.g., computer-generated algorithm, random number table). The term "digital random assignment" is vague and lacks methodological specifics.

R2 (High risk): No information is provided on allocation concealment (e.g., use of sealed envelopes or central randomization). The absence of safeguards introduces significant selection bias risk.

R3 (Low risk): Baseline characteristics (Table 1) show no significant differences between groups in age, height, weight, BDI scores, or PSQI scores (e.g., age: SG 65.3±2.9 vs. CG 66.2±3.4, P>0.05), supporting the effectiveness of randomization.

Consensus: Some concerns​

Rationale: The vague description of sequence generation and complete lack of allocation concealment details introduce uncertainty, but the baseline balance partially mitigates the risk. Overall, some concerns remain due to inadequate reporting of randomization procedures.

D2 — Bias due to deviations from the intended interventions​

R1 (Some concerns): The intervention (Tai Chi) is behavioral, and blinding of participants was not feasible, potentially affecting adherence. However, exercises were supervised with standardized protocols (5 sessions/week for 24 weeks) and heart rate monitoring.

R2 (Some concerns): The document does not mention whether outcome assessors were blinded. Primary outcomes include subjective measures (BDI and PSQI scores) and objective serum biomarkers (TNF-α, IL-6, 5-HT). Lack of assessor blinding may introduce detection bias, particularly for subjective components.

Consensus: Some concerns​

Rationale: The inability to blind participants and unclear assessor blinding for subjective outcomes introduce performance and detection bias risks, though standardized protocols provide partial mitigation.

D3 — Bias due to missing outcome data​

R1 (Low risk): The missing data rate is low (135 randomized, 124 completed, 8.1% attrition), with reasons documented (personal or family reasons).

R2 (Some concerns): No intention-to-treat (ITT) analysis or specific methods for handling missing data (e.g., multiple imputation) are described. Analyses appear per-protocol.

R3 (Some concerns): The low attrition rate (<10%) supports low risk, but the lack of ITT analysis introduces slight uncertainty. Overall, the risk is manageable.

Consensus: Some concerns​

Rationale: While low attrition minimizes bias risk, the absence of ITT methods warrants caution.

D4 — Bias in measurement of the outcome​

R1 (Some concerns): Primary outcomes include subjective patient-reported measures (BDI and PSQI scores) and objective laboratory measures (serum TNF-α, IL-6, 5-HT). The subjectivity of BDI and PSQI increases susceptibility to measurement bias.

R2 (Some concerns): No information is provided on assessor blinding, training, or standardization of assessment procedures for subjective outcomes. The document mentions "assessment" but lacks methodological details.

Consensus: Some concerns​

Rationale: Subjective measurement tools without robust blinding protocols introduce measurement bias risk, though objective biomarkers partially offset this concern.

D5 — Bias in selection of the reported result​

R1 (High risk): The document mentions ethical approval but does not indicate prospective trial registration (e.g., in a clinical trials registry). Only submission and acceptance dates (2024) are referenced.

R2 (Low risk): All pre-specified outcomes (BDI, PSQI, serum biomarkers) are fully reported with statistical results, including non-significant findings and longitudinal data (baseline, 12 weeks, 24 weeks).

R3 (Some concerns): Lack of prospective registration introduces significant uncertainty, but comprehensive outcome reporting aligns with methods.

Consensus: Some concerns​

Overall (Consensus): Some concerns​

Rationale: The worst-rated domains are D1, D2, D3, D4, and D5 ("Some concerns"). Key limitations include inadequate randomization details (D1), lack of blinding information for subjective outcomes (D2), no ITT analysis (D3), measurement bias risk in subjective components (D4), and no prospective registration (D5). However, the low attrition rate, baseline balance, and standardized intervention delivery partially mitigate risks. The study's design has strengths in objective biomarker measurements, but the biases warrant cautious interpretation of the subjective primary outcomes (depression and sleep quality). The risks do not invalidate the results but highlight the need for careful consideration.

**23.ROB2 Assessment for Chen et al., 2013**

D1 — Bias arising from the randomization process

R1 (Some concerns): The document states (random sampling method) for participant selection and then (through random grouping). However, it does not describe the specific method of random sequence generation (e.g., computer-generated, random number table) for assigning participants to groups. The process is not detailed.

R2 (High risk): The document provides no​ description of allocation concealment (e.g., use of sealed envelopes or central randomization).

R3 (Low risk): The baseline characteristics indicate that the gender composition and average age between the two groups showed no significant difference (P>0.05), supporting comparability.

Consensus: Some concerns

Rationale: The lack of detail on the random sequence generation method and the complete absence of allocation concealment introduce uncertainty about the randomization integrity, despite baseline comparability.

D2 — Bias due to deviations from the intended interventions

R1 (Some concerns): The intervention (Baduanjin) is behavioral, and blinding of participants was not feasible, potentially affecting adherence. The intervention was standardized and supervised.

R2 (High risk): The document does not mention​ whether outcome assessors (the investigators filling the forms) were blinded. The primary outcome (SCL-90 scores) is a subjective patient-reported measure. Lack of assessor blinding for subjective outcomes introduces a high risk of detection bias.

R3 (High risk): The combination of an unblinded behavioral intervention and unblinded assessment of subjective outcomes creates a high risk of performance and detection bias.

Consensus: High risk

Rationale: The inability to blind participants, combined with the lack of assessor blinding for the key subjective patient-reported outcome, results in a high risk of bias due to deviations.

D3 — Bias due to missing outcome data

R1 (High risk): Initial numbers: 180 participants were selected, with 80 assigned to the exercise group and 88 to the control group. However, the final analysis included 80 per group. The document states 12 participants were excluded from the control group during the experiment due to significant changes in habits or health, but the final count discrepancy and handling of these exclusions are unclear. Attrition/exclusion appears substantial and is not fully accounted for.

R2 (High risk): The document does not​ describe the use of intention-to-treat (ITT) analysis or any specific methods for handling missing data or exclusions. The analysis appears to be a per-protocol analysis.

Consensus: High risk

Rationale: The unclear handling of participant flow (exclusion of 12 from control, final group sizes) and the absence of ITT analysis result in a high risk of bias from missing data.

D4 — Bias in measurement of the outcome

R1 (High risk): The primary outcome (SCL-90 scores) is a subjective patient-reported measure. Its measurement is susceptible to bias.

R2 (High risk): As noted in D2, there is no mention of outcome assessor blinding. The data collection involved investigators filling in the forms, which, without blinding, could influence the recording.

Consensus: High risk

Rationale: The measurement of the outcome is based on unblinded subjective reporting, which carries a high risk of bias.

D5 — Bias in selection of the reported result

R1 (Some concerns): The document does not​ indicate prospective trial registration. It only mentions dates of submission and revision (2012).

R2 (Low risk): All outcomes mentioned in the methods (SCL-90 factor scores) appear to be fully reported in the results table (Table 1) for both groups before and after the intervention.

Consensus: Some concerns

Rationale: The lack of a prospective protocol introduces uncertainty about pre-specification. However, all measured outcomes seem to be reported.

Overall (Consensus): High risk

Rationale: Domains D2, D3, and D4 are judged to be at High risk​ of bias. The critical limitations are: the lack of blinding for subjective outcomes (D2, D4), unclear handling of exclusions and lack of ITT analysis (D3), and insufficient detail on randomization (D1). While the reported outcomes appear comprehensive (D5), the high risks in core domains related to intervention implementation, missing data, and outcome measurement fundamentally compromise the reliability of the estimated intervention effects. The results should be interpreted with great caution.

**24.ROB2 Assessment for Zhao et al., 2015**

D1 — Bias arising from the randomization process

R1 (Some concerns): The document states participants were (randomly divided into experimental and control groups) but provides no description of the random sequence generation method (e.g., computer-generated, random number table).

R2 (High risk): The document lacks any description of allocation concealment (e.g., use of sealed envelopes or central randomization).

R3 (Low risk): Baseline characteristics (Table 1) show good balance between groups in gender, education level, marital status, income, family relationships, and living environment, supporting the effectiveness of randomization.

Consensus: Some concerns

Rationale: The absence of details on random sequence generation and complete lack of allocation concealment introduce uncertainty, but the baseline balance mitigates the risk. Overall, some concerns remain.

D2 — Bias due to deviations from the intended interventions

R1 (Some concerns): The intervention (24-form Tai Chi) is behavioral, and blinding of participants was not feasible. The intervention was standardized (3 sessions/week, 30 min/session for 1 year) with initial training and supervision.

R2 (High risk): The document does not mention whether outcome assessors were blinded. The primary outcome (GDS score) is a subjective patient-reported measure, and lack of assessor blinding may introduce detection bias.

R3 (Some concerns): Lack of participant blinding may introduce behavioral bias, but the supervised delivery alleviates some risk. Concerns persist due to the subjective primary outcome.

Consensus: Some concerns

Rationale: The inability to blind participants and unclear assessor blinding for the subjective primary outcome introduce performance and detection bias risks.

D3 — Bias due to missing outcome data

R1 (Low risk): The document reports that all 52 participants (26 per group) completed the study, indicating 0% attrition.

R2 (Some concerns): No intention-to-treat (ITT) analysis or specific methods for handling missing data are described. Analyses appear per-protocol.

R3 (Low risk): With no missing data, the risk of attrition bias is minimal.

Consensus: Some concerns

Rationale: While no attrition supports low risk, the lack of ITT analysis introduces theoretical uncertainty. Overall, the risk is manageable.

D4 — Bias in measurement of the outcome

R1 (High risk): The primary outcome (depression severity) is measured entirely by the Geriatric Depression Scale (GDS), a subjective patient-reported measure.

R2 (High risk): No information is provided on assessor blinding, training, or standardization of assessment procedures. The document states participants "filled out the form," but the collection/scoring process is not detailed.

Consensus: High risk

Rationale: Subjective measurement tools without any described blinding protocols introduce a high risk of measurement bias for the primary outcome.

D5 — Bias in selection of the reported result

R1 (High risk): The document mentions funding but does not indicate prospective trial registration (e.g., in a clinical trials registry).

R2 (Low risk): All pre-specified outcomes (GDS scores, treatment efficacy categories) are fully reported with statistical results, including between-group comparisons and longitudinal data.

R3 (High risk): Lack of prospective registration introduces significant uncertainty, but comprehensive outcome reporting aligns with methods.

Consensus: Some concerns

Overall (Consensus): High risk

Rationale: Domain D4 is rated High risk​ due to the critical limitation of using a subjective primary outcome measure (GDS) without any described blinding of participants or assessors, fundamentally compromising the objectivity of the results. Domains D1, D2, D3, and D5 are rated "Some concerns" due to inadequate randomization details, lack of blinding information, no ITT analysis, and no prospective registration. While the study demonstrates strengths in baseline balance, standardized intervention, and no attrition, the high risk in outcome measurement (D4) is a dominant concern that severely undermines the validity of the estimated intervention effect on depression. The findings should be interpreted with extreme caution.

**25.ROB2 Assessment for Siu et al., 2025**

D1 — Bias arising from the randomization process

R1 (Low risk): The document explicitly describes the random sequence generation method: "Randomisation was conducted using an online random generator... with block sizes of four to six." The computer-generated sequence indicates a standardized and reproducible process.

R2 (Low risk): The document provides a clear description of robust allocation concealment: "The computer generated randomised allocation sequence was securely placed in sealed opaque envelopes and kept by an independent researcher... A second independent researcher... needed to contact the first... to retrieve the allocation sequence after the baseline assessments and before the start of the interventions." This process effectively prevents foreknowledge of the upcoming assignment.

Consensus: Low risk

Rationale: The clear description of computer-generated random sequence, a robust allocation concealment mechanism using sealed envelopes managed by independent personnel, and excellent baseline balance collectively ensure a low risk of selection bias.

D2 — Bias due to deviations from the intended interventions

R1 (Some concerns): The interventions (Tai Chi and CBT-I) are behavioral, and blinding of participants was not feasible, potentially affecting adherence. The interventions were standardized, manualized, and delivered in a group format by certified instructors/therapists.

R2 (Low risk): The document explicitly states: "Outcome assessors were blinded to the group allocation and participants were instructed not to disclose their group allocation to the outcome assessors during the outcome assessments." This reduces measurement bias.

R3 (Some concerns): Lack of participant blinding may introduce behavioral bias (performance bias), as participants' knowledge of receiving Tai Chi or CBT-I could influence their behavior, expectations, or co-interventions. The risk of deviation from the intended intervention due to this is present but mitigated by the standardized, instructor-led group format.

Consensus: Some concerns

Rationale: The inability to blind participants is an inherent limitation of behavioral intervention trials, introducing a risk of performance bias. However, the implementation of assessor blinding and standardized intervention protocols reduces the risk of detection bias and ensures intervention fidelity. Overall, some concerns remain.

D3 — Bias due to missing outcome data

R1 (Some concerns): Attrition was observed: Of 200 participants randomised, 166 (83%) completed the month 3 assessment, and 167 (83.5%) completed the month 15 assessment. This represents an overall attrition of 16-17% at each time point.

R2 (Low risk): The document states that the "per protocol principle was adopted" for the primary non-inferiority analysis. Importantly, it also reports that "Results from the intention-to-treat analysis were consistent with the per protocol findings." Sensitivity analyses using Last Observation Carried Forward (LOCF) and Multiple Imputation (MI) methods were also conducted, and results were consistent.

R3 (Some concerns): The attrition rate (≈17%) is not trivial. While multiple analytical approaches (Per Protocol, ITT, LOCF, MI) yielded consistent results—which strengthens the findings—the potential for bias if data are not missing completely at random remains a consideration.

Consensus: Some concerns

Rationale: The use of both per-protocol and ITT analyses, along with sensitivity analyses (LOCF, MI), and the consistency of findings across these methods, appropriately addresses the missing data. However, the level of attrition warrants a cautious interpretation. Overall, the risk is considered manageable, leading to a rating of "Some concerns".

D4 — Bias in measurement of the outcome

R1 (Some concerns): The primary outcome is the Insomnia Severity Index (ISI), a subjective patient-reported measure. Its measurement is susceptible to bias.

R2 (Low risk): As noted in D2, outcome assessors were blinded to group allocation. The ISI is a self-reported questionnaire, so the role of the blinded assessor was likely limited to administration/data collection, reducing measurement bias in that process.

R3 (Some concerns): The main concern is the subjective nature of the ISI. Participants were not blinded, and their expectations or beliefs about Tai Chi vs. CBT-I could influence their self-reported scores. Assessor blinding protects against bias in scoring but not against bias originating from the participant.

Consensus: Some concerns

Rationale: The use of a subjective primary outcome measure in a trial where participants are not blinded introduces a risk of measurement bias. This is partially mitigated by the use of a validated scale (ISI) and blinded assessors, but a degree of uncertainty remains.

D5 — Bias in selection of the reported result

R1 (Low risk): The trial was prospectively registered at ClinicalTrials.gov (NCT04384822) on 12 May 2020, before participant enrolment began (18 May 2020). A detailed protocol paper was also published.

R2 (Low risk): All pre-specified primary and secondary outcomes mentioned in the methods are fully reported in the results (Tables 2 & 3, and Figure 3 for remission/response rates), including non-significant findings for secondary outcomes and detailed longitudinal data.

Consensus: Low risk

Rationale: Prospective registration, a published protocol, and comprehensive reporting of all outcomes indicate a low risk of selective reporting.

Overall (Consensus): Some concerns

Rationale: The worst-rated domains are D2, D3, and D4 ("Some concerns"). The study demonstrates significant methodological strengths: a low-risk randomization process with allocation concealment (D1), prospective registration and transparent reporting (D5), and the use of blinded outcome assessors. The primary limitations are inherent to behavioral intervention trials: the inability to blind participants (creating potential for performance and measurement bias in subjective outcomes, D2/D4) and a non-negligible attrition rate (D3). However, these risks are mitigated by robust study design features such as assessor blinding, the use of both per-protocol and ITT analyses with sensitivity analyses, and the pre-registration of outcomes. The overall study design is rigorous, and the bias risks do not invalidate the results but warrant their considered interpretation.

**26.ROB2 Assessment for Wen et al., 2023**

D1 — Bias arising from the randomization process

R1 (Low risk): The document explicitly describes the random sequence generation method: "a permuted-block randomization method was used" and "The computer-generated random allocation sequence was controlled by an independent researcher... The randomization procedure(block size=4) was implemented with an allocation ratio of 1:1". This indicates a standardized and reproducible process.

R2 (Low risk): The document provides a clear description of allocation concealment: "Allocation concealment was assured by using sealed opaque envelopes that were distributed by other researchers." The sequence was controlled by an independent researcher.

Consensus: Low risk

Rationale: The clear description of a computer-generated permuted-block sequence, robust allocation concealment using sealed opaque envelopes managed independently, and excellent baseline balance collectively ensure a low risk of selection bias.

D2 — Bias due to deviations from the intended interventions

R1 (Some concerns): The intervention (Baduanjin Qigong) is behavioral, and blinding of participants was not feasible, potentially affecting adherence. The intervention was standardized, supervised initially, and included remote monitoring via video conferences.

R2 (Low risk): The document explicitly mentions assessor blinding: "All data collectors were blinded to the group allocation" and "The outcome assessor was unaware of the participants' group allocation."

R3 (Some concerns): Lack of participant blinding may introduce performance bias (e.g., differential enthusiasm, co-interventions). However, the use of a true usual care control group, standardized protocol, and blinded outcome assessment significantly mitigates the risk of detection bias.

Consensus: Some concerns

Rationale: The inability to blind participants is an inherent limitation of behavioral intervention trials, introducing a risk of performance bias. However, the implementation of assessor blinding and a controlled intervention protocol reduces the overall risk. Concerns persist but are not high.

D3 — Bias due to missing outcome data

R1 (Some concerns): Attrition was observed: 8/44 (18.2%) in the Baduanjin group and 5/44 (11.4%) in the control group withdrew, yielding an overall attrition of 14.8% (13/88). Reasons for withdrawal are documented in the flowchart.

R2 (Low risk): The document explicitly states that both intention-to-treat (ITT) and per-protocol (PP) analyses were performed: "In accordance with CONSORT guidelines, both intention-to-treat(ITT) and per-protocol(PP) analyses were performed..." For ITT, "a regression analysis approach to impute missing data" was used.

R3 (Some concerns): The attrition rate is not trivial and is slightly higher in the intervention group. However, the pre-specified use of both ITT (with imputation) and PP analyses appropriately addresses the impact of missing data.

Consensus: Some concerns

Rationale: The use of rigorous analytical methods (both ITT with imputation and PP) mitigates the risk introduced by attrition. The consistency of findings between both analyses strengthens the results. The risk is therefore manageable, leading to a rating of "Some concerns".

D4 — Bias in measurement of the outcome

R1 (Some concerns): The primary outcome (Quality of Life via FACT-H&N) and key secondary outcomes (fatigue, anxiety, depression, sleep quality) are subjective patient-reported measures.

R2 (Low risk): As noted in D2, outcome assessors were blinded to group allocation during data collection.

R3 (Some concerns): The main concern is the subjectivity of the outcomes. Participants were not blinded, and their expectations could influence their self-reported scores. Assessor blinding protects against bias in data collection but not against bias originating from the participant.

Consensus: Some concerns

Rationale: The use of subjective primary and secondary outcome measures in a trial where participants are not blinded introduces a risk of measurement bias. This is partially mitigated by the use of validated scales and blinded assessors, but a degree of uncertainty remains.

D5 — Bias in selection of the reported result

R1 (Low risk): The study was conducted according to a protocol (implied by CONSORT adherence and ethics approval). While a public trial registration number is not explicitly mentioned in the provided text, the detailed methodology and analysis plan suggest a pre-specified approach.

R2 (Low risk): All outcomes mentioned in the methods section (QOL, complications, fatigue, sleep, anxiety, depression) are fully reported in the results for both ITT and PP populations, including non-significant findings (e.g., functional well-being in ITT analysis, anxiety/depression between-group comparisons).

Consensus: Low risk

Rationale: The comprehensive reporting of all pre-specified primary and secondary outcomes for both analytical populations, along with detailed statistical results, indicates a low risk of selective reporting.

Overall (Consensus): Some concerns

Rationale: The worst-rated domains are D2, D3, and D4 ("Some concerns"). The study demonstrates significant methodological strengths: a low-risk randomization process with allocation concealment (D1), prospective use of both ITT and PP analyses (D3), and transparent reporting (D5). The primary limitations are inherent to behavioral intervention trials: the inability to blind participants (creating potential for performance and measurement bias in subjective outcomes, D2/D4) and a non-negligible attrition rate (D3). However, these risks are mitigated by key design features such as assessor blinding, the use of a control group, and rigorous pre-planned analyses. The overall study design is rigorous, and the bias risks do not invalidate the results but warrant their considered interpretation.

**27.ROB2 Assessment for Park et al., 2023**

D1 — Bias arising from the randomization process

R1 (Low risk): The document explicitly describes the random sequence generation method: "Participants were allocated using computerized randomization software in a 1:1 ratio..." indicating a standardized and reproducible process.

R2 (Some concerns): Although the sequence generation is clear, the document lacks a detailed description of allocation concealment (e.g., use of sealed envelopes or central randomization). It only states the allocation was done via software, but does not specify the mechanism to conceal the sequence until after participant enrollment.

R3 (Low risk): The baseline characteristics table (Table 1) shows no statistically significant differences between groups in demographic and clinical variables, supporting the effectiveness of randomization.

Consensus: Some concerns

Rationale: The absence of allocation concealment details introduces uncertainty, but the baseline balance and clear sequence generation mitigate the risk. Overall, some concerns remain, but it is not high risk.

D2 — Bias due to deviations from the intended interventions

R1 (Some concerns): The interventions (Qigong/Tai Chi and light exercise) are behavioral, and blinding of participants was not feasible, potentially affecting adherence.

R2 (Low risk): The document mentions assessor blinding: "assessor-blinded pilot RCT." The primary outcome (MADRS score) is a clinician-rated measure, and assessor blinding protects against measurement bias in its administration.

R3 (Some concerns): Lack of participant blinding may introduce behavioral bias. However, the objective of comparing two active behavioral interventions and the use of standardized, instructor-led group sessions mitigate some risk. Concerns persist overall due to the subjective nature of the primary outcome.

Consensus: Some concerns

Rationale: While assessor blinding reduces detection bias, the inability to blind participants for behavioral interventions introduces performance bias risk.

D3 — Bias due to missing outcome data

R1 (Low risk): The document states 23 participants were randomized and that "all randomized participants completed follow-up assessments," indicating 0% attrition for outcome data collection.

R2 (Low risk): The document explicitly states: "An intent-to-treat analysis was performed."

Rationale: Complete follow-up and the use of ITT analysis support a low risk of bias.

D4 — Bias in measurement of the outcome

R1 (Low risk): The primary outcome (depressive symptoms) was measured using the Montgomery-Asberg Depression Rating Scale (MADRS), a standardized clinician-administered interview.

R2 (Low risk): As noted in D2, outcome assessors were blinded, reducing human bias in the administration and scoring of the MADRS.

Consensus: Low risk

Rationale: Assessor blinding for the primary clinical interview provides strong protection against measurement bias.

D5 — Bias in selection of the reported result

R1 (Low risk): The trial was prospectively registered on ClinicalTrials.gov (NCT04450147) prior to participant recruitment.

R2 (Low risk): All pre-specified primary, secondary, and exploratory outcomes are fully reported in the results (Table 2), including non-significant findings.

Consensus: Low risk

Rationale: Prospective registration and comprehensive outcome reporting indicate a low risk of selective reporting.

Overall (Consensus): Some concerns

Rationale: The worst-rated domains are D1 and D2 ("Some concerns"). Although the objective measurement of the primary outcome (D4), handling of missing data (D3), and reporting transparency (D5) are low risk, uncertainties from insufficient allocation concealment details (D1) and potential performance bias from lack of participant blinding (D2) introduce limitations. However, the overall study design is rigorous (computerized randomization, assessor blinding, ITT analysis, prospective registration), and these bias risks do not invalidate the results of this pilot RCT.

**28.ROB2 Assessment for Ma et al., 2018**

D1 — Bias arising from the randomization process

R1 (Low risk): The document explicitly describes the randomization procedure: eligible participants "were asked to select an opaque envelope to allocate whether they would be in the TC group or the UC group." This implies a random allocation sequence was pre-determined and concealed within envelopes.

R2 (Low risk): The document provides a clear description of allocation concealment: "There was one card in each envelope... All participants and healthcare professionals were masked to the group assignment." The use of sequentially numbered, opaque, sealed envelopes effectively conceals the allocation sequence until after assignment.

R3 (Low risk): The baseline characteristics table (Table 1) shows no statistically significant differences between the Tai Chi (TC) and usual care (UC) groups in all measured demographic and clinical variables, supporting the effectiveness of randomization.

Consensus: Low risk

Rationale: The use of a random allocation method with sealed opaque envelopes ensures robust allocation concealment. The excellent balance in baseline characteristics further confirms the randomization process was effective. Overall, the risk of selection bias is low.

D2 — Bias due to deviations from the intended interventions

R1 (Some concerns): The intervention (group-based Tai Chi) is behavioral, and blinding of participants was not feasible, potentially affecting adherence and expectations.

R2 (Low risk): The document states: "All participants and healthcare professionals were masked to the group assignment." It also specifies that nurses collecting outcome data "were not involved in the study." This indicates outcome assessors (nurses measuring BP, etc.) were likely blinded, reducing detection bias for objective outcomes.

R3 (Some concerns): Lack of participant blinding may introduce performance bias. However, the intervention was highly standardized (trained professional instructors, specific syllabus, 24-form Tai Chi) and adherence was monitored via group logs and attendance cards. The control group received "usual care" without a specific exercise regimen, which is appropriate.

Consensus: Some concerns

Rationale: The inability to blind participants is an inherent limitation of behavioral trials, introducing a risk of performance bias. However, the blinding of healthcare professionals/assessors and the standardized, monitored intervention delivery mitigate the risk of bias due to deviations. Overall, some concerns remain.

D3 — Bias due to missing outcome data

R1 (Some concerns): Attrition was notable: 158 participants were randomized, 113 completed the 6-month study, resulting in a 28.5% attrition rate. The flow diagram (Fig. 1) indicates more dropouts in the UC group (n=21) than the TC group (n=24), but the difference is not large.

R2 (Low risk): The document explicitly states: "The analyses were conducted based on intention-to-treat, with missing values imputed based on an assumption of no change." This is an appropriate, pre-specified method for handling missing data in the ITT analysis.

R3 (Some concerns): The high attrition rate (28.5%) is a concern. While ITT with a "no change" imputation was used, this method is conservative and may underestimate effects if dropout is related to the intervention or outcomes.

Consensus: Some concerns

Rationale: The application of a pre-specified ITT analysis with a defined imputation method appropriately addresses the missing data. However, the high attrition rate introduces uncertainty, particularly regarding whether the "no change" assumption is valid. The risk is therefore considered manageable but present.

D4 — Bias in measurement of the outcome

R1 (Low risk): Primary outcomes include objective measurements: Blood Pressure (BP) was measured with a calibrated sphygmomanometer by trained nurses using a standard protocol; Body Mass Index (BMI) and waist circumference were measured with standardized tools.

R2 (Low risk): As noted in D2, outcome assessors (nurses) were blinded to group allocation. Measurement protocols for BP, height, weight, and waist circumference were standardized.

Consensus: Low risk

Rationale: Objective measurement techniques, blinded assessors, and detailed, standardized measurement protocols provide strong protection against measurement bias for the primary physical health outcomes.

D5 — Bias in selection of the reported result

R1 (Some concerns): The document (published 2018) does not mention prospective trial registration in a public registry (e.g., ClinicalTrials.gov). It states ethical approval was obtained and the study was funded.

R2 (Low risk): All outcomes pre-specified in the methods (BP, BMI, waist circumference, social support, depressive symptoms, quality of life via SF-36 subscales) are fully reported in the results tables (Tables 2, 3, 4), including non-significant findings (e.g., waist circumference, several SF-36 subscales).

R3 (Some concerns): The lack of a prospective protocol or registration in a public trials registry introduces some uncertainty about whether all analyses were pre-specified. However, the comprehensive reporting of all measured outcomes suggests a low likelihood of selective reporting based on the results obtained.

Consensus: Some concerns

Overall (Consensus): Some concerns

Rationale: Domains D2 and D3 are rated as having Some concerns. The primary limitations are the inability to blind participants (performance bias risk, D2) and the high attrition rate, despite the use of ITT analysis (D3). The study has important strengths: a well-described randomization process with allocation concealment (D1: Low risk), blinded assessment of objective primary outcomes (D4: Low risk), and transparent reporting of results (D5: Some concerns). The overall design is rigorous for a behavioral intervention trial. While bias cannot be ruled out, the methodological strengths support the credibility of the main findings regarding the effects of group-based Tai Chi on blood pressure, BMI, and psychosocial outcomes in this population. The results should be interpreted with the noted limitations in mind.

**29.ROB2 Assessment for Liu et al., 2025**

D1 — Bias arising from the randomization process

R1 (Low risk): The document explicitly describes the random sequence generation method: "Randomization was conducted by independent researchers via an automated permuted block algorithm with a block size of 4." This indicates a standardized and reproducible computerized process.

R2 (Some concerns): The document does not provide a detailed description of allocation concealment (e.g., the use of sequentially numbered, opaque, sealed envelopes or a central randomization system). It only states that participants were "randomly assigned" after baseline assessment. The lack of detail on the mechanism to conceal the allocation sequence introduces some uncertainty.

R3 (Low risk): The baseline characteristics table (Table 1) shows no statistically significant differences between the experimental and sham groups across all measured demographic and clinical variables, supporting the effectiveness of randomization in creating comparable groups.

Consensus: Some concerns

Rationale: The use of a computer-generated permuted block randomization is a strength. However, the absence of specific details regarding allocation concealment introduces some uncertainty about the potential for selection bias. The excellent baseline balance partially mitigates this concern. Overall, some concerns remain due to inadequate reporting of concealment methods.

D2 — Bias due to deviations from the intended interventions

R1 (Some concerns): The interventions include a behavioral component (Tai Chi Chuan) and a device-based neuromodulation (rTMS). Blinding of participants to the Tai Chi component was not feasible. However, for the rTMS component, a sham coil was used, and the document states: "The tai chi chuan coach was unaware of the group allocation and the nature of the intervention." This suggests an attempt to blind the coach.

R2 (Low risk): The document explicitly states: "The outcome evaluators and statistical analysts were masked from group allocation." This protects against detection bias in outcome assessment and analysis.

R3 (Some concerns): Lack of participant blinding to the Tai Chi component may introduce performance bias (e.g., differential expectations, adherence). The use of a validated sham rTMS procedure and the blinding of the Tai Chi coach are mitigating factors. The risk of deviation from the intended protocol is considered low due to the standardized, supervised nature of both interventions.

Consensus: Some concerns

Rationale: The inability to blind participants to the exercise intervention is an inherent limitation, introducing a risk of performance bias. However, the successful blinding of outcome assessors and the use of a sham-controlled rTMS design with a blinded coach significantly reduce the risks of detection bias and ensure intervention fidelity. Overall, some concerns remain regarding performance bias.

D3 — Bias due to missing outcome data

R1 (Low risk): Attrition was low. Of 110 participants randomized, 103 (93.6%) completed the 6-week intervention and 12-week follow-up, indicating a 6.4% dropout rate.

R2 (Low risk): The document explicitly describes rigorous methods for handling missing data: "All participants who provided informed consent were included in the intention-to-treat (ITT) analysis... Multiple imputation was used for missing data on primary outcome measures at baseline, after the intervention, and at follow-up." Both ITT (with multiple imputation) and per-protocol analyses were performed, with consistent results.

Consensus: Low risk

Rationale: Minimal attrition and the application of rigorous statistical methods (ITT with multiple imputation) to handle missing data ensure a low risk of bias in this domain.

D4 — Bias in measurement of the outcome

R1 (Some concerns): The primary outcomes include both subjective patient-reported measures (Pittsburgh Sleep Quality Index, PSQI) and a clinician-administered cognitive assessment (Montreal Cognitive Assessment, MoCA). While the MoCA is standardized, both outcomes involve an element of subjective reporting or assessment.

R2 (Low risk): As noted in D2, outcome assessors were blinded to group allocation. The use of validated scales (PSQI, MoCA) and actigraphy for objective sleep parameters further standardizes measurement.

R3 (Some concerns): The main concern is the subjective nature of the PSQI. Participants were not blinded, which could influence their self-reported sleep scores. However, the blinding of outcome assessors for the MoCA and the use of objective actigraphy data mitigate measurement bias for other endpoints.

Consensus: Some concerns

Rationale: The use of blinded assessors for the MoCA and actigraphy is a strength. For the subjective PSQI, the lack of participant blinding introduces a risk of measurement bias due to expectation effects. This concern is partially offset by the use of a validated instrument and blinded assessors for data collection.

D5 — Bias in selection of the reported result

R1 (Low risk): The trial was prospectively registered on the Chinese Clinical Trial Registry (Identifier: ChiCTR2200063274). A detailed study protocol and statistical analysis plan were also available (Supplement 1).

R2 (Low risk): All pre-specified primary and secondary outcomes mentioned in the methods are fully reported in the results section (Tables 2, 3, 4) for all time points (baseline, 6 weeks, 12 weeks), including non-significant findings (e.g., Trail Making Test Part B at 6 weeks, several actigraphy parameters).

Consensus: Low risk

Rationale: Prospective registration, availability of a protocol, and comprehensive reporting of all outcomes indicate a low risk of selective reporting.

Overall (Consensus): Some concerns

Rationale: Domains D1, D2, and D4 are rated as having Some concerns. The primary limitations are the lack of detailed allocation concealment (D1), the inherent risk of performance bias due to the inability to blind participants to the Tai Chi intervention (D2), and the potential for measurement bias in the self-reported primary sleep outcome (PSQI) due to lack of participant blinding (D4). The study demonstrates significant strengths: computer-generated randomization with good baseline balance, blinded outcome assessment and statistical analysis (D2, D4), low attrition with rigorous ITT and multiple imputation analysis (D3: Low risk), prospective registration, and transparent reporting of all results (D5: Low risk). The overall study design is rigorous for a complex behavioral-neuromodulation trial. While the noted concerns warrant cautious interpretation, particularly for the subjective sleep outcomes, the methodological strengths support the credibility of the main findings regarding the additive benefit of active rTMS over sham rTMS when combined with Tai Chi Chuan.

**30.ROB2 Assessment for Tou et al., 2024**

D1 — Bias arising from the randomization process

R1 (Low risk): The document explicitly describes the random sequence generation method: "Randomization sequence was generated based on a computerized block randomization with block sizes of 4." This indicates a standardized and reproducible process.

R2 (Some concerns): The document states the sequence "was concealed from personnel involved in recruitment of participants." However, it lacks a detailed description of the allocation concealment mechanism (e.g., use of sequentially numbered, opaque, sealed envelopes or central randomization). The method of implementing the allocation sequence is not specified.

R3 (Low risk): The baseline characteristics table (Table 1) shows no statistically significant differences between the intervention and control groups in all measured demographic and clinical variables, supporting the effectiveness of randomization.

Consensus: Some concerns

Rationale: The clear description of computer-generated block randomization and excellent baseline balance are strengths. However, the absence of specific details regarding the allocation concealment procedure introduces some uncertainty. Overall, the risk is not high, but some concerns remain.

D2 — Bias due to deviations from the intended interventions

R1 (Some concerns): The intervention (Baduanjin Qigong) is behavioral, and blinding of participants was not feasible, potentially affecting adherence. The exercise protocol was standardized, supervised by certified instructors, and included safety monitoring (blood pressure, heart rate).

R2 (Low risk): The document explicitly states it is an "assessor-blinded" trial and that "All outcome measures were conducted at baseline and 4-month follow-up by trained assessors who were blinded to the participants' group allocation." This reduces the risk of detection bias.

R3 (Some concerns): Lack of participant blinding may introduce behavioral bias. The control group received monthly health education talks and was instructed to maintain usual activity, which is an appropriate comparator. The standardized, supervised delivery mitigates the risk of deviation from the intended protocol.

Consensus: Some concerns

Rationale: The inability to blind participants is an inherent limitation, introducing performance bias risk. However, the implementation of assessor blinding and a controlled intervention protocol reduces the overall risk. Concerns persist but are not high.

D3 — Bias due to missing outcome data

R1 (Low risk): Of 57 participants randomized, 7 dropped out (12.3% attrition), leaving 50 who completed the study. Attrition was slightly higher in the intervention group (4/29, 13.8%) than the control group (3/28, 10.7%), but the difference is small.

R2 (Low risk): The document explicitly states: "Analyses were conducted based on intention-to-treat principle, and all participants with completed baseline outcome measures were included in the analyses." Linear mixed-effect models, which handle missing data under the missing-at-random assumption, were employed.

Consensus: Low risk

Rationale: The use of ITT analysis with linear mixed models appropriately addresses the missing data. The attrition rate, while not trivial, is considered manageable with the analytical methods used.

D4 — Bias in measurement of the outcome

R1 (Some concerns): The primary outcomes include a mix of objective measures (knee extension strength via dynamometer) and subjective patient-reported measures (Maastricht Questionnaire for vital exhaustion, Falls Efficacy Scale-International for fear of falling). The subjectivity of the latter two introduces susceptibility to bias.

R2 (Low risk): As noted in D2, outcome assessors were blinded to group allocation. Measurement procedures for all outcomes (physical tests and questionnaires) are described in detail and standardized.

R3 (Some concerns): The objectivity of the physical performance measures is a strength. For the subjective questionnaires, the blinding of assessors who administered them reduces bias. However, the participants themselves were not blinded, which could influence their self-reported scores for exhaustion and fear of falling.

Consensus: Some concerns

Rationale: The risk of measurement bias is mixed. It is low for the objective physical measures but of some concern for the subjective patient-reported outcomes due to the lack of participant blinding, despite assessor blinding.

D5 — Bias in selection of the reported result

R1 (Low risk): The trial was prospectively registered on ClinicalTrials.gov (NCT04549103) on 16 September 2020, prior to participant enrollment.

R2 (Low risk): All pre-specified primary and secondary outcomes mentioned in the methods are fully reported in the results (Table 2) for both groups at baseline and 4-month follow-up, including non-significant findings for all between-group comparisons. The study also transparently reports exploratory compliance and within-group analyses.

Consensus: Low risk

Rationale: Prospective registration, adherence to CONSORT guidelines, and comprehensive reporting of all outcomes, including null results, indicate a low risk of selective reporting.

Overall (Consensus): Some concerns

Rationale: Domains D1, D2, and D4 are rated as having Some concerns. The main limitations relate to insufficient detail on allocation concealment (D1), the inherent risk of performance bias due to the inability to blind participants (D2), and the potential for measurement bias in the subjective components of the primary outcome (D4). The study demonstrates significant strengths: computer-generated randomization with excellent baseline balance, assessor blinding (D2), appropriate handling of missing data with ITT analysis and mixed models (D3: Low risk), prospective registration, and transparent reporting of all results (D5: Low risk). The overall study design is rigorous. While the noted concerns warrant cautious interpretation, particularly for the subjective outcomes (vital exhaustion, fear of falling), the methodological strengths support the credibility of the main finding that the 16-week community-based Baduanjin program did not demonstrate significant between-group differences in physical performance, psychological measures, or frailty status in this population.
