# Supplementary material for: Effects of traditional Chinese mind–body exercises on depressive symptoms in middle-aged and older adults: a multilevel meta-analysis with exploratory dose–response and machine learning analyses
Source: Front Psychol. 2026 Jul 13;17:1887582. doi: 10.3389/fpsyg.2026.1887582 (PMC13402133; doi:10.3389/fpsyg.2026.1887582)
Supplement: Supplementary file 2 [file Supplementary_file_2.DOCX]

**Table of Contents | Supplementary Materials**

[Table S1. Supplementary document Retrieval Strategy 3](#_Toc232961698)

[Table S2. Risk of Bias Assessment Using the Cochrane RoB 2 Tool 4](#_Toc232961699)

[Text S1. Study selection consistency and Cohen’s κ 5](#_Toc232961700)

[Table S3. Inter-Rater Agreement Results for Each ROB2 Domain 6](#_Toc232961701)

[Fig S1. Variance Decomposition and Multilevel I² Contributions 7](#_Toc232961702)

[Fig S2. Egger’s Test for Publication Bias (Primary Analysis) 7](#_Toc232961703)

[Fig S3. Standardized Residuals and Cook’s Distance (Primary Analysis) 8](#_Toc232961704)

[Fig S4. Leave-one-out Sensitivity Analysis (Primary Analysis) 9](#_Toc232961705)

[Fig S5. Egger’s Test for Publication Bias After Trim-and-Fill Adjustment 9](#_Toc232961706)

[Fig S6. Standardized Residuals and Cook’s Distance After Outlier Removal 10](#_Toc232961707)

[Fig S7. Leave-one-out Sensitivity Analysis 10](#_Toc232961708)

[Fig S8. Trim-and-Fill Funnel Plot 10](#_Toc232961709)

[Fig S9. Trial Sequential Analysis (TSA) of the Cumulative Evidence 11](#_Toc232961710)

[Table S4. GRADE Assessment of the Certainty of Evidence for the Effects of Traditional Chinese Mind–Body Exercise on Depressive Symptoms in Middle-aged and Older Adults 11](#_Toc232961711)

[Table S5. Subgroup analyses for depressive symptoms outcomes in middle-aged and older adults 12](#_Toc232961712)

[Fig S9. Bayesian dose–response model diagnostics and posterior distribution of the exploratory low-point dose 14](#_Toc232961713)

[Fig S9A. Posterior distribution of the exploratory low-point dose 14](#_Toc232961714)

[Fig S9B. Posterior predictive density check 15](#_Toc232961715)

[Fig S9C. Posterior predictive interval check 16](#_Toc232961716)

[Fig S9D. Posterior predictive ECDF check 17](#_Toc232961717)

[Fig S10. Performance diagnostics of the XGBoost prediction model 18](#_Toc232961718)

[Fig S10A. Performance diagnostics of the XGBoost prediction model 18](#_Toc232961719)

[Fig S10B. Learning Curve 19](#_Toc232961720)

[Fig S11. SHAP dependence plots for continuous predictors in the XGBoost model 20](#_Toc232961721)

[Fig S11A. Exercise frequency 20](#_Toc232961722)

[Fig S11B. Intervention duration 21](#_Toc232961723)

[Fig S11C. Exercise time 22](#_Toc232961724)

[Fig S11D. Total intervention dose 23](#_Toc232961725)

[Fig S11E. Participant age 24](#_Toc232961726)

[Fig S12. SHAP dependence plots for categorical study-level predictors in the XGBoost model 25](#_Toc232961727)

[Fig S12A. Country 25](#_Toc232961728)

[Fig S12B. Population type 26](#_Toc232961729)

[Fig S12C. Population stage 27](#_Toc232961730)

[Fig S12D. Intervention type 28](#_Toc232961731)

[Fig S12E. Comparator type 29](#_Toc232961732)

[Fig S12F. Scale type 30](#_Toc232961733)

[PRISMA_2020_checklist 30](#_Toc232961734)

[Reference 34](#_Toc232961735)

# Table S1. Supplementary document Retrieval Strategy

| **Data** | **Query** | **Results** |
| --- | --- | --- |
| **PubMed** | ((("Traditional Chinese mind-body exercise"[Title/Abstract] OR "mind-body exercise"[Title/Abstract] OR "Traditional Chinese exercise"[Title/Abstract] OR "Tai Chi"[Title/Abstract] OR "Taiji"[Title/Abstract]OR"Qigong"[Title/Abstract] OR "Baduanjin"[Title/Abstract] OR "Wu Qin Xi"[Title/Abstract] OR "Yijinjing"[Title/Abstract] OR "Liuzijue"[Title/Abstract] OR "Daoyin"[Title/Abstract] OR"Qigong therapy"[Title/Abstract])) AND (("depression"[Title/Abstract] OR "depressive symptoms"[Title/Abstract] OR "depressive disorder"[Title/Abstract] OR "mood"[Title/Abstract] OR "mental health"[Title/Abstract])) AND (("middle-aged adults"[Title/Abstract] OR "older adults"[Title/Abstract] OR elderly[Title/Abstract] OR aging[Title/Abstract] OR ageing[Title/Abstract] OR "middle-aged people"[Title/Abstract] OR "older people"[Title/Abstract] OR "middle-aged and older adults"[Title/Abstract] OR "middle-aged adults"[Title/Abstract] OR "older adults"[Title/Abstract]) AND ("randomized controlled trial"[Publication Type] OR randomized[Title/Abstract] OR randomised[Title/Abstract] OR RCT[Title/Abstract])) | 139 |
| **Web of Science** | TS=(("Traditional Chinese mind-body exercise" OR "mind-body exercise" OR "Traditional Chinese exercise" OR "Tai Chi" OR "Taiji" OR "Qigong" OR "Qigong therapy" OR "Baduanjin" OR "Wu Qin Xi" OR "Wuqinxi" OR "Yijinjing" OR "Liuzijue" OR "Daoyin") AND ("depression" OR "depressive symptoms" OR "depressive disorder" OR "depressive" OR "mood" OR "mental health") AND (("middle-aged adults" OR "older adults" OR elderly OR aging OR ageing OR "middle-aged people" OR "older people" OR "community-dwelling older adults" OR "middle-aged and older adults" OR "middle-aged people" OR "older adults") AND ("randomized controlled trial" OR randomized OR randomised OR RCT)) | 460 |
| **Embase** | (('traditional chinese mind-body exercise':ti,ab,kw OR 'mind-body exercise':ti,ab,kw OR 'traditional chinese exercise':ti,ab,kw OR 'tai chi':ti,ab,kw OR 'taiji':ti,ab,kw OR 'qigong':ti,ab,kw OR 'baduanjin':ti,ab,kw OR 'wu qin xi':ti,ab,kw OR 'yijinjing':ti,ab,kw OR 'liuzijue':ti,ab,kw OR 'daoyin':ti,ab,kw OR 'qigong therapy':ti,ab,kw)) AND (('depression':ti,ab,kw OR 'depressive symptoms':ti,ab,kw OR 'depressive disorder':ti,ab,kw OR 'mood':ti,ab,kw OR 'mental health':ti,ab,kw)) AND (('middle-aged adults':ti,ab,kw OR 'older adults':ti,ab,kw OR elderly:ti,ab,kw OR aging:ti,ab,kw OR ageing:ti,ab,kw OR 'middle-aged people':ti,ab,kw OR 'older people':ti,ab,kw OR 'middle-aged and older adults':ti,ab,kw)) AND (('randomized controlled trial':ti,ab,kw OR RCT:ti,ab,kw OR 'randomised controlled trial':ti,ab,kw OR 'randomized trial':ti,ab,kw)) | 112 |
| **Cochrane Library** | #1 ("Traditional Chinese mind-body exercise" OR "mind-body exercise" OR "Traditional Chinese exercise" OR "Tai Chi" OR "Taiji" OR "Qigong" OR "Qigong therapy" OR "Baduanjin" OR "Wu Qin Xi" OR "Wuqinxi" OR "Yijinjing" OR "Liuzijue" OR "Daoyin"):ti,ab,kw  #2 ("depression" OR "depressive symptoms" OR "depressive disorder" OR "depressive" OR "mood" OR "mental health"):ti,ab,kw  #3 ("middle-aged adults" OR "older adults" OR elderly OR aging OR ageing OR "middle-aged people" OR "older people" OR "middle-aged individuals" OR "older individuals" OR "community-dwelling older adults" OR "middle-aged and older adults"):ti,ab,kw  #1 AND #2 AND #3 | 265 |
| **CNKI** | #1 主题 =(中国传统身心运动 + 身心运动 + 中国传统运动 + 太极 + 太极拳 + 太极运动 + 气功 + 气功疗法 + 八段锦 + 五禽戏 + 易筋经 + 六字诀 + 导引术)  #2 主题 =(抑郁 + 抑郁症 + 抑郁情绪 + 抑郁症状 + 心境障碍 + 心理健康)  #3 主题 =(中老年 + 中老年人 + 老年 + 中年)  #4 主题 =(随机 + 对照 + RCT + 随机对照试验 + 对照试验 + 临床试验 + 随机研究)  #5：#1 AND #2 AND #3 AND #4 | 41 |
| **VIP** | M=(中国传统身心运动 OR 身心运动 OR 中国传统运动 OR 太极 OR 太极拳 OR 太极运动 OR 气功 OR 气功疗法 OR 八段锦 OR 五禽戏 OR 易筋经 OR 六字诀 OR 导引术) AND M=(抑郁 OR 抑郁症 OR 抑郁情绪 OR 抑郁症状 OR 心境障碍 OR 心理健康) AND M=(中老年 OR 中老年人 OR 老年 OR 中年) AND M=(随机 OR 对照 OR RCT OR 随机对照试验 OR 对照试验 OR 临床试验 OR 随机研究) | 16 |
| **Wanfang** | 主题:(中国传统身心运动 OR 身心运动 OR 中国传统运动 OR 太极 OR 太极拳 OR 太极运动 OR 气功 OR 气功疗法 OR 八段锦 OR 五禽戏 OR 易筋经 OR 六字诀 OR 导引术) AND 主题:(抑郁 OR 抑郁症 OR 抑郁情绪 OR 抑郁症状 OR 心境障碍 OR 心理健康) AND 主题:(中老年 OR 中老年人 OR 老年 OR 中年) AND 主题:(随机 OR 对照 OR RCT OR 随机对照试验 OR 对照试验 OR 临床试验 OR 随机研究) | 416 |

**Note:** CNKI, Chinese National Knowledge Infrastructure; VIP, Chongqing VIP Database; Wanfang, Wanfang Data Platform.

**Additional strategy: We screened the reference lists of included RCTs and relevant reviews/meta-analyses and conducted forward citation tracking of key trials.**

# Table S2. Risk of Bias Assessment Using the Cochrane RoB 2 Tool

| **Authors** | **D1** | **D2** | **D3** | **D4** | **D5** | **Overall** |
| --- | --- | --- | --- | --- | --- | --- |
| Song et al. 2022 (1) | Low risk | Some concerns | Some concerns | Some concerns | Low risk | Some concerns |
| Dong et al. 2013 (2) | Some concerns | Some concerns | Some concerns | Some concerns | Some concerns | Some concerns |
| Liao et al. 2015 (3) | Some concerns | Some concerns | Some concerns | Some concerns | Some concerns | Some concerns |
| Ma et al.2016 (4) | Some concerns | High risk | Some concerns | Some concerns | Some concerns | High risk |
| Mo et al. 2016 (5) | Some concerns | High risk | Some concerns | Some concerns | Some concerns | High risk |
| Xu et al. 2025 (6) | Some concerns | Some concerns | Some concerns | Low risk | Some concerns | Some concerns |
| Yang et al 2023 (7) | Some concerns | Some concerns | Some concerns | Low risk | Some concerns | Some concerns |
| Ma et al. 2010 (8) | Some concerns | Some concerns | Some concerns | Some concerns | Some concerns | Some concerns |
| Shan et al. 2025 (9) | Some concerns | High risk | Some concerns | Some concerns | Some concerns | High risk |
| Luo et al. 2021 (10) | Some concerns | High risk | Some concerns | High risk | Some concerns | High risk |
| Ma et al. 2011 (11) | Some concerns | Some concerns | Some concerns | Some concerns | Some concerns | Some concerns |
| Wen et al. 2024 (12) | Some concerns | Some concerns | Some concerns | Some concerns | Some concerns | Some concerns |
| Wei et al. 2022 (13) | Some concerns | Some concerns | Some concerns | Some concerns | Low risk | Some concerns |
| Larkey et al. 2015 (14) | Some concerns | Some concerns | Some concerns | Some concerns | Some concerns | Some concerns |
| Z. Chen et al. 2013 (15) | Some concerns | Some concerns | Some concerns | Low risk | Some concerns | Some concerns |
| Sun et al. 2019 (16) | Some concerns | Some concerns | Some concerns | Some concerns | Some concerns | Some concerns |
| Shi et al. 2025 (17) | Some concerns | Some concerns | Some concerns | Some concerns | Some concerns | Some concerns |
| Ni et al. 2021 (18) | Some concerns | Some concerns | Some concerns | High risk | Some concerns | High risk |
| Li et al. 2022 (19) | Some concerns | Some concerns | Some concerns | Low risk | Some concerns | Some concerns |
| J. Liu et al. 2025 (20) | Some concerns | Some concerns | Low risk | Some concerns | Low risk | Some concerns |
| Carcelén-Fraile et al. 2022 (21) | Some concerns | Some concerns | Some concerns | Some concerns | Low risk | Some concerns |
| Chang et al. 2024 (22) | Some concerns | Some concerns | Some concerns | Some concerns | Some concerns | Some concerns |
| W. Chen et al. 2013 (23) | Some concerns | High risk | High risk | High risk | Some concerns | High risk |
| Zhao et al. 2015 (24) | Some concerns | Some concerns | Some concerns | High risk | Some concerns | High risk |
| Siu et al. 2025 (25) | Low risk | Some concerns | Some concerns | Some concerns | Low risk | Some concerns |
| Wen et al. 2023 (26) | Low risk | Some concerns | Some concerns | Some concerns | Low risk | Some concerns |
| Park et al. 2023 (27) | Some concerns | Some concerns | Low risk | Low risk | Low risk | Some concerns |
| Ma et al. 2018 (28) | Low risk | Some concerns | Some concerns | Low risk | Some concerns | Some concerns |
| Z. Liu et al. 2025 (29) | Some concerns | Some concerns | Low risk | Some concerns | Low risk | Some concerns |
| Tou et al. 2024 (30) | Some concerns | Some concerns | Low risk | Some concerns | Low risk | Some concerns |

# Text S1. Study selection consistency and Cohen’s κ

1. Title/abstract screening (records screened = 950)

Independent 2×2 table (X.Y. vs J.G.): both include = 110; X.Y. include / J.G. exclude = 10; X.Y. exclude / J.G. include = 18; both exclude = 812 (total n = 950; reports sought for retrieval a + b + c = 138, consistent with the PRISMA flowchart).

Observed agreement: P*o* = (110 + 812) / 950 = 0.9705.

Expected agreement: P*e* ≈ 0.7735.

Cohen’s κ ≈ 0.870 (more precisely 0.8702).

Approximate standard error: SE ≈ 0.024.

95% CI: κ ≈ 0. 870, 95% CI ≈ 0.823–0.917.

z ≈ 36.3 (p＜0.001).

Interpretation (Landis & Koch): κ ≈ 0.87, indicating substantial agreement.

2. Full-text screening (reports assessed = 120)

Independent 2×2 table X.Y. vs J.G.: both include = 28; X.Y. include / J.G. exclude = 4; X.Y. exclude / J.G. include = 2; both exclude = 86 (total n = 120; final included studies a = 28, with 2 additional records included after discussion/adjudication, resulting in 30 included RCTs, consistent with the PRISMA flowchart).

Observed agreement: P*o* = (28 + 86) / 120 = 0.9500.

Expected agreement: P*e* ≈ 0.6167.

Cohen’s κ ≈ 0.870 (more precisely 0.8696).

Approximate standard error: SE ≈ 0.052.

95% CI: κ ≈ 0.870, 95% CI ≈ 0.768–0.971.

z ≈ 16.7 (p＜0.001).

Interpretation (Landis & Koch): κ ≈ 0.87, indicating substantial agreement.

# Table S3. Inter-Rater Agreement Results for Each ROB2 Domain

The table below displays the simple agreement rate, Cohen's Kappa, and Weighted Kappa calculated separately for each domain (D1-D5).

| **Domain** | **Simple Agreement Rate** | **Cohen’s κ** | **Weighted κ** | **Strength of Agreement** |
| --- | --- | --- | --- | --- |
| **D1 (Randomization process)** | 30.0% (9/30) | 0.12 | 0.21 | Slight–Fair |
| **D2 (Deviations from intended interventions)** | 53.3 % (16/30) | 0.31 | 0.46 | Fair- Moderate |
| **D3 (Missing outcome data)** | 16.7 % (5/30) | 0.05 | 0.14 | Slight |
| **D4 (Measurement of the outcome)** | 60.0 % (18/30) | 0.41 | 0.56 | Moderate |
| **D5 (Selection of the reported result)** | 30.0 % (9/30) | 0.15 | 0.24 | Slight–Fair |


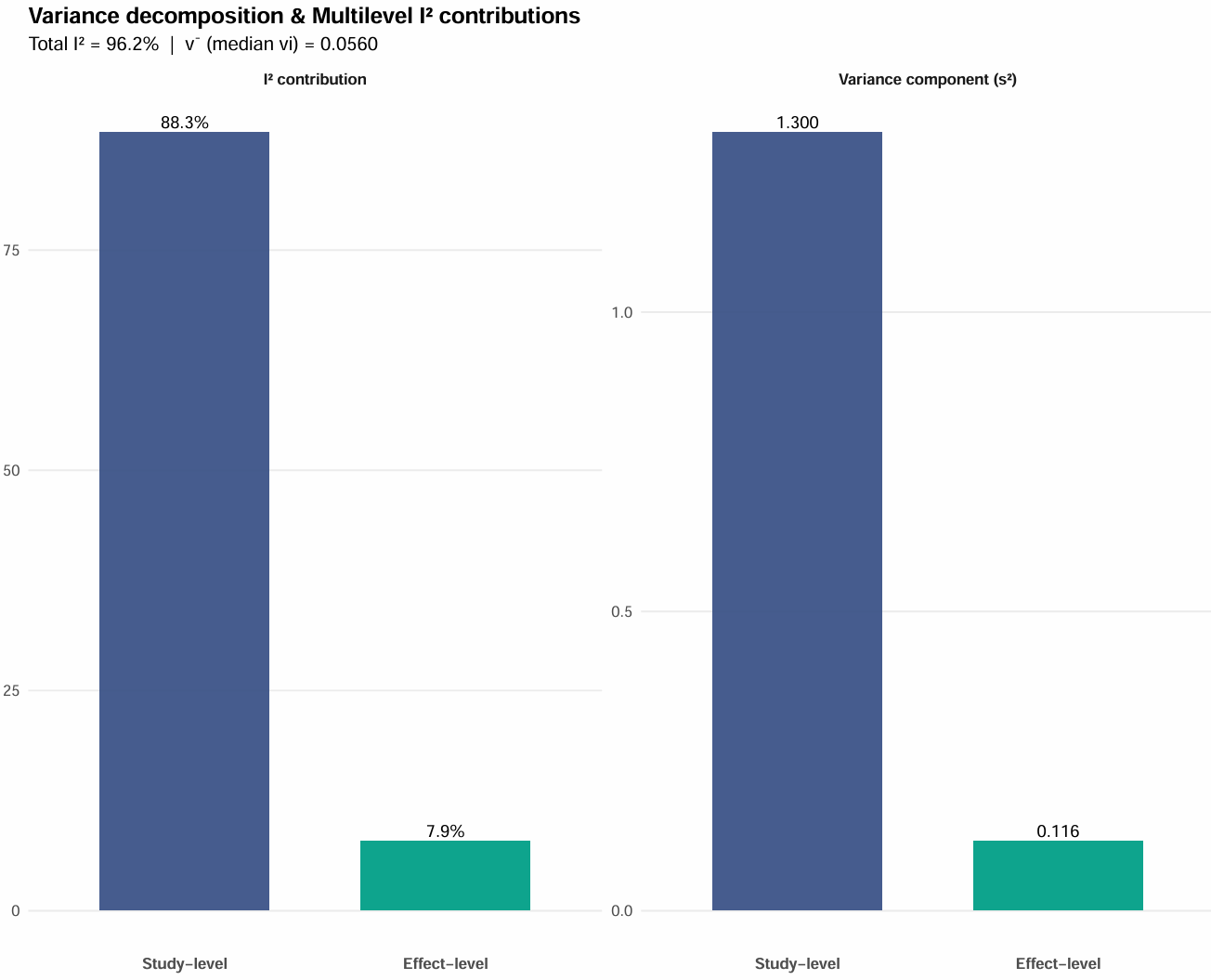


# Fig S1. Variance Decomposition and Multilevel I² Contributions


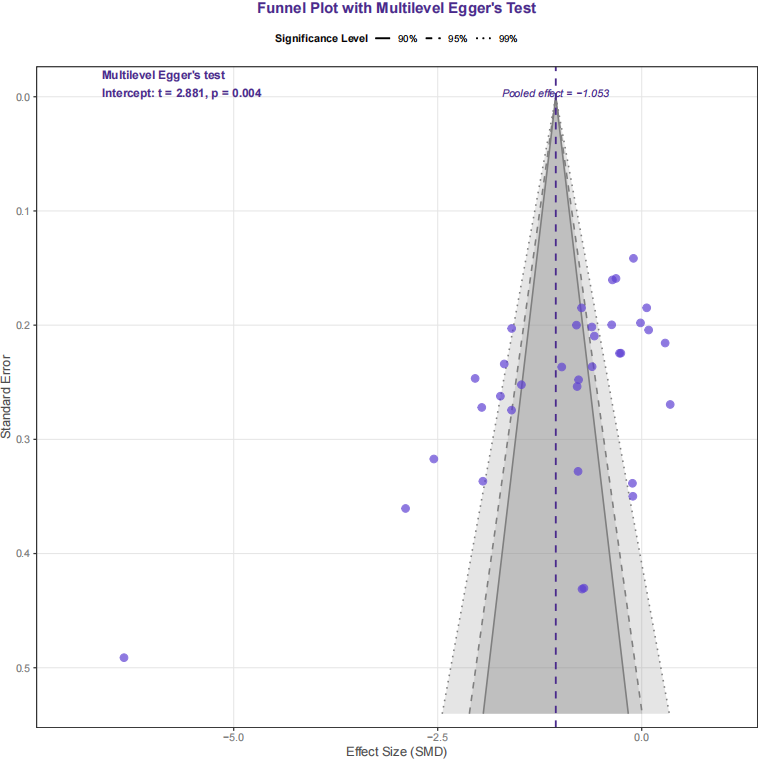


# Fig S2. Egger’s Test for Publication Bias (Primary Analysis)


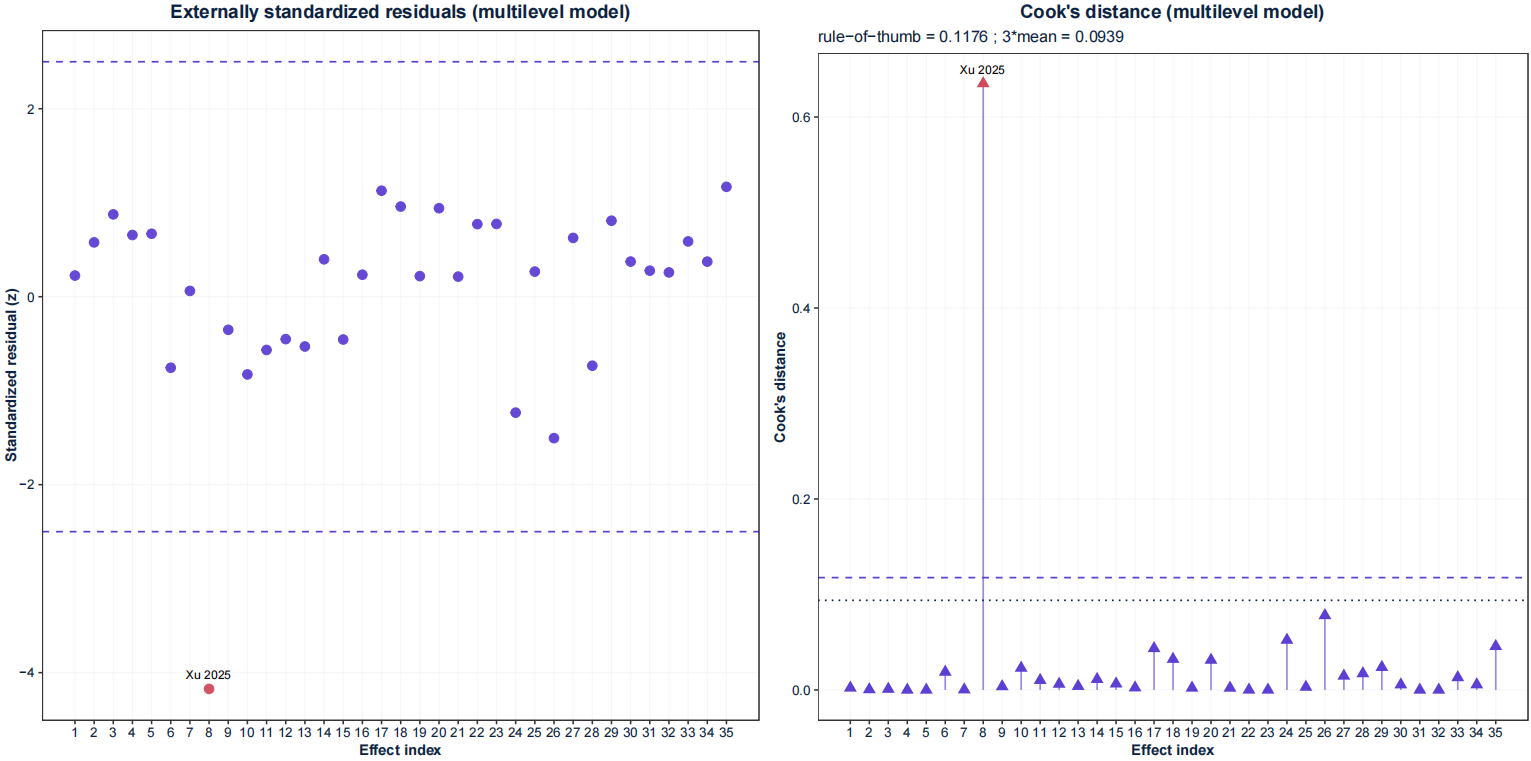


# Fig S3. Standardized Residuals and Cook’s Distance (Primary Analysis)


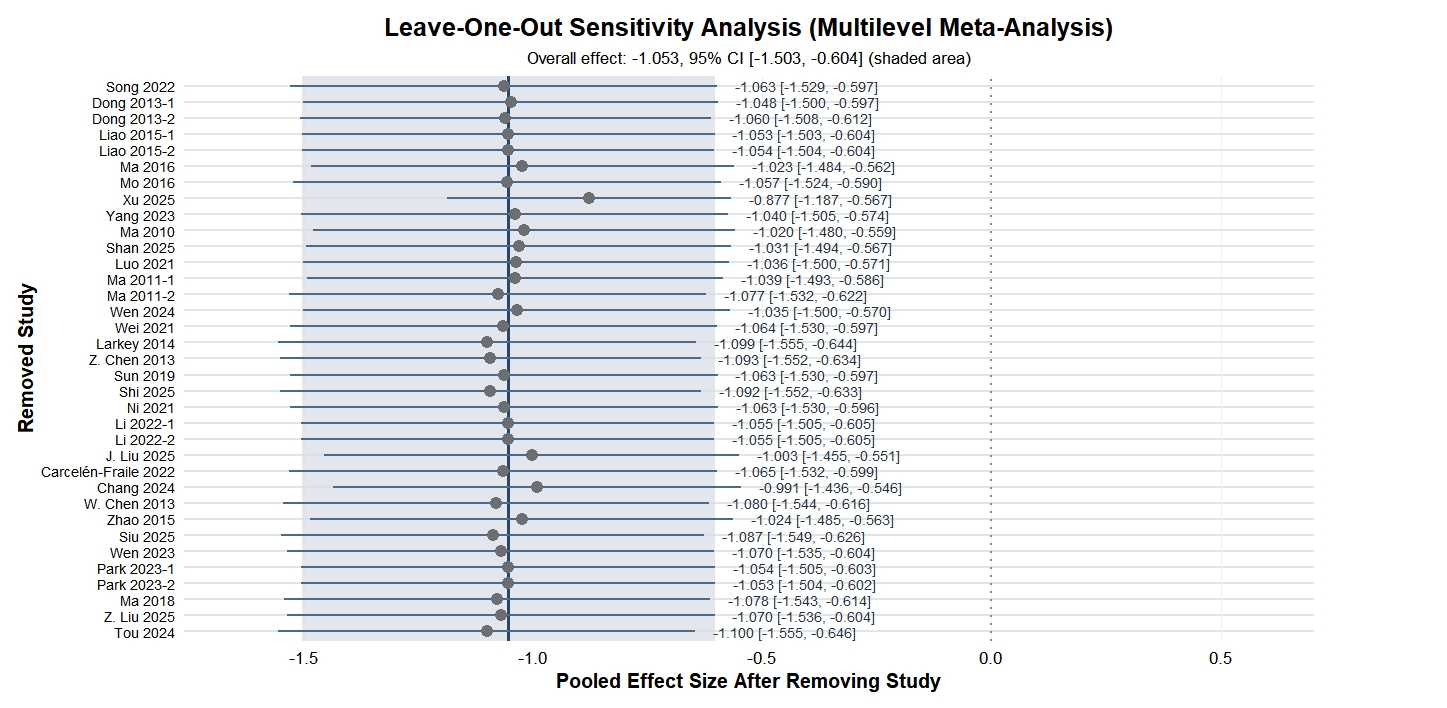


# Fig S4. Leave-one-out Sensitivity Analysis (Primary Analysis)


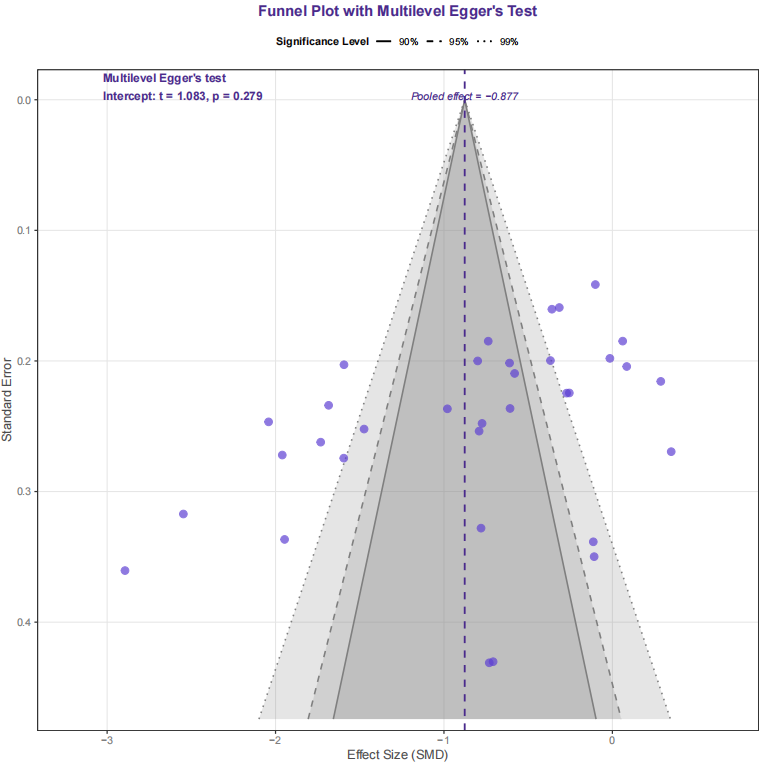


# Fig S5. Egger’s Test for Publication Bias After Trim-and-Fill Adjustment


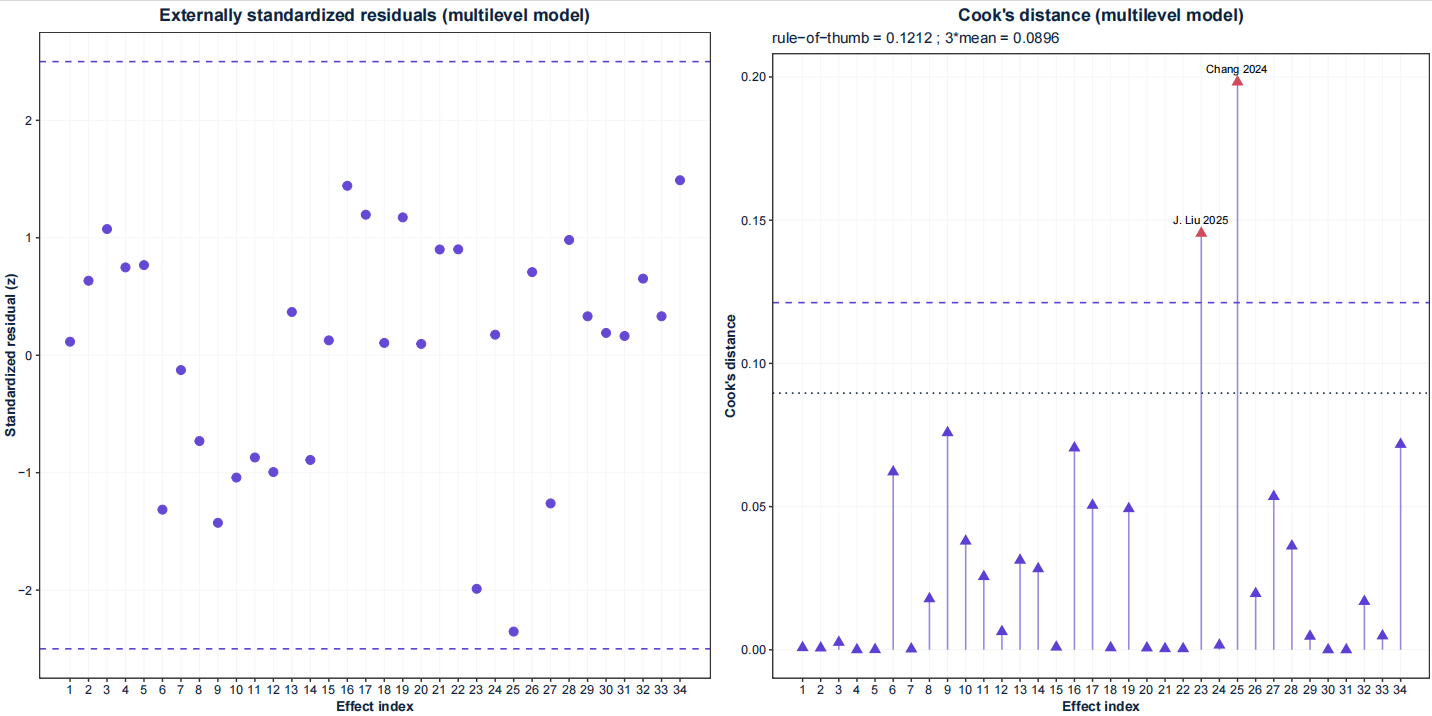


# Fig S6. Standardized Residuals and Cook’s Distance After Outlier Removal


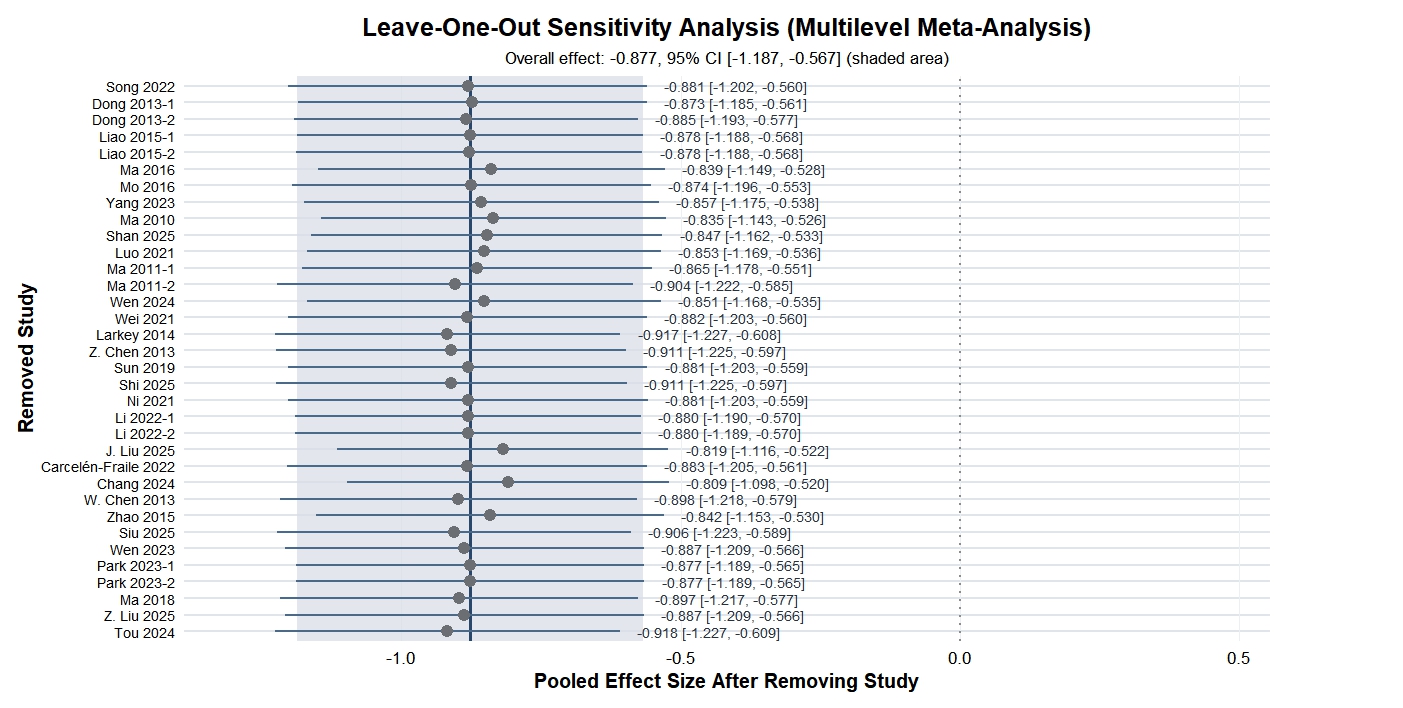


# Fig S7. Leave-one-out Sensitivity Analysis


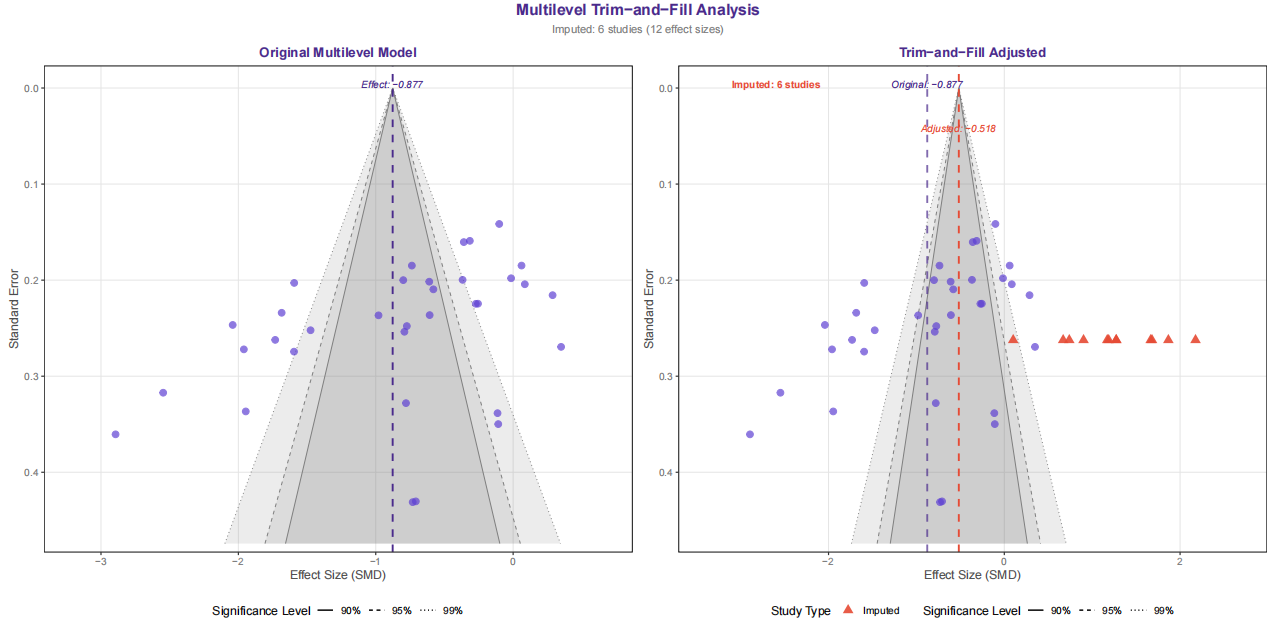


# Fig S8. Trim-and-Fill Funnel Plot


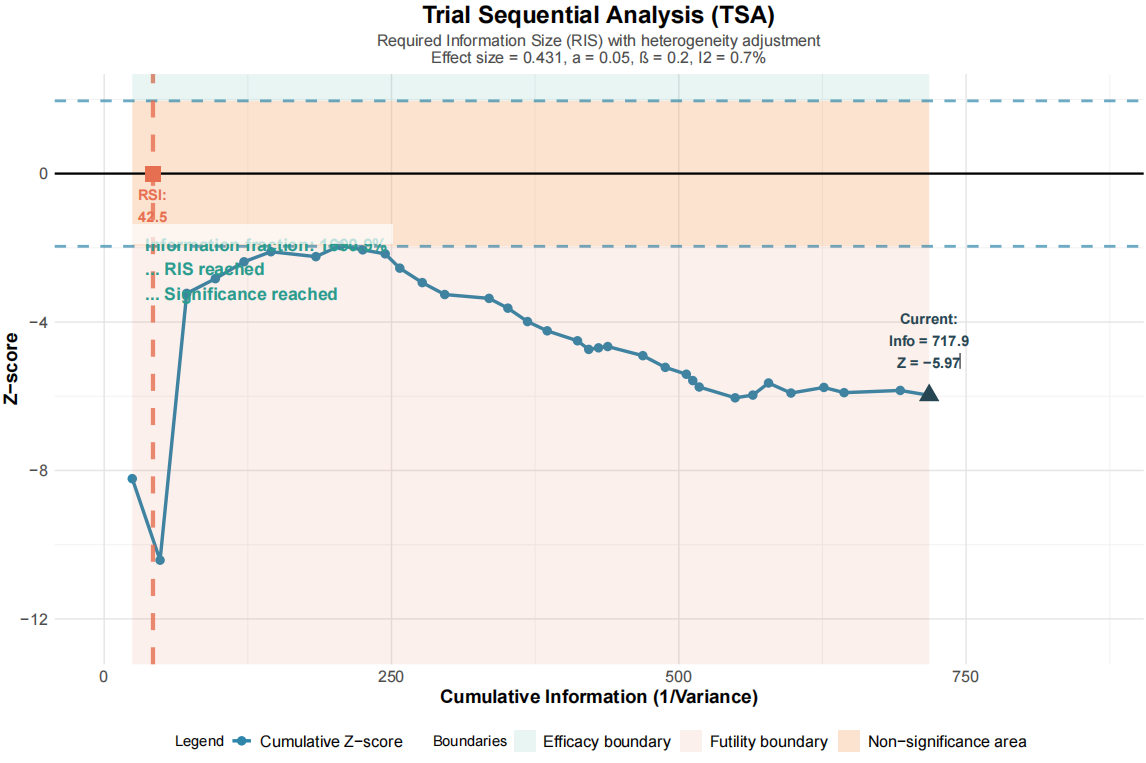


# Fig S9. Trial Sequential Analysis (TSA) of the Cumulative Evidence

# Table S4. GRADE Assessment of the Certainty of Evidence for the Effects of Traditional Chinese Mind–Body Exercise on Depressive Symptoms in Middle-aged and Older Adults

| **GRADE Domain** | **Judgment** | **Rationale (Strictly Based on the Original Article)** |
| --- | --- | --- |
| Risk of Bias | Serious (Downgraded by 1 level) | Among the 30 included RCTs, most studies were rated as “some concerns,” with only a few classified as low risk and several classified as high risk. Key concerns were mainly related to randomization procedures, deviations from intended interventions, missing outcome data, and outcome measurement. Therefore, the certainty of evidence was downgraded by one level for risk of bias. |
| Inconsistency | Serious (Downgraded by 1 level) | Substantial heterogeneity was observed in the primary analysis Q = 467.31, P < 0.001, with a total I² of 96.2%. Variance decomposition further showed that heterogeneity was mainly attributable to between-study differences, with the between-study variance accounting for 91.8% of total heterogeneity. Therefore, the certainty of evidence was downgraded by one level for inconsistency. |
| Indirectness | Not downgraded | The population (middle-aged and older adults), intervention (traditional Chinese mind–body exercises), comparators, and outcomes (standardized depression scales) were directly aligned with the research question, indicating good applicability. |
| Imprecision | Not downgraded | A total of 30 studies (35 effect sizes) were included. The pooled effect remained statistically significant both before sensitivity adjustment SMD = −1.05, 95% CI: −1.49 to −0.62 and after outlier removal SMD = −0.88, 95% CI: −1.18 to −0.58. The confidence intervals did not cross zero, and leave-one-out sensitivity analyses showed stable pooled effects. Therefore, no downgrade was applied for imprecision. |
| Publication Bias | Not downgraded | Egger’s test initially suggested potential publication bias P = 0.004, but it was no longer significant after removing the influential study P = 0.279. However, the possibility of small-study effects or publication bias could not be fully excluded, particularly because trim-and-fill analysis imputed 6 studies (11 effect sizes) before the funnel plot became more symmetrical. Considering that the main effect remained robust across sensitivity analyses, p-curve analysis, TSA, and CRVE, no additional downgrade was applied for publication bias, but this domain was interpreted cautiously. |
| Overall | Low | Overall certainty of evidence was rated as **low**, mainly because the evidence was downgraded by one level for risk of bias and one level for inconsistency. |

# Table S5. Subgroup analyses for depressive symptoms outcomes in middle-aged and older adults

| **Dimensionality** | **Subgroup** | **k** | **N** | **SMD (95% CI)** | **p-value** | **I² (%)** | **GRADE** | **P-interaction** |
| --- | --- | --- | --- | --- | --- | --- | --- | --- |
| **Country** | China | 27 | 1204 | -0.97 (-1.28, -0.67) | <0.001 | 90.6 | Low | **0.001** |
|  | Other Asian Countries | 3 | 129 | -0.04 (-0.43, 0.34) | 0.833 | 57.6 | Low |  |
|  | Western Countries | 4 | 131 | -0.43 (-0.99, 0.14) | 0.137 | 79.3 | Very low |  |
| **Exercise Frequency** | ≤3 sessions/week | 15 | 620 | -0.58 (-0.92, -0.24) | <0.001 | 85.2 | Low | **0.084** |
|  | 4–6 sessions/week | 12 | 527 | -0.86 (-1.39, -0.33) | 0.002 | 90.7 | Low |  |
|  | ≥7 sessions/week | 7 | 317 | -1.30 (-1.84, -0.76) | <0.001 | 91.6 | Low |  |
| **Intervention Duration** | ≤8 weeks | 6 | 248 | -1.00 (-1.57, -0.43) | <0.001 | 89.3 | Low | **0.425** |
|  | 9–16 weeks | 20 | 840 | -0.67 (-1.01, -0.34) | <0.001 | 89.8 | Low |  |
|  | >16 weeks | 8 | 376 | -1.09 (-1.78, -0.40) | 0.002 | 92.2 | Low |  |
| **Session Duration** | ≤30 min | 9 | 382 | -1.16 (-1.59, -0.73) | <0.001 | 88.1 | Low | **0.261** |
|  | 31–45 min | 7 | 264 | -0.73 (-1.35, -0.12) | 0.02 | 91.1 | Low |  |
|  | ≥60 min | 18 | 818 | -0.70 (-1.10, -0.30) | <0.001 | 89.4 | Low |  |
| **Age group** | Middle-aged (45–59) | 16 | 639 | -1.02 (-1.42, -0.61) | <0.001 | 91.5 | Low | **0.190** |
|  | Older adults (≥60) | 18 | 825 | -0.66 (-1.02, -0.30) | <0.001 | 86.8 | Low |  |
| **Intervention Type** | Tai Chi | 9 | 412 | -1.10 (-1.78, -0.41) | 0.002 | 92.9 | Low | **0.777** |
|  | Qigong | 5 | 251 | -0.59 (-1.29, 0.12) | 0.104 | 91 | Very low |  |
|  | Baduanjin | 7 | 327 | -0.81 (-1.40, -0.21) | 0.008 | 91.2 | Low |  |
|  | Combined intervention | 13 | 474 | -0.77 (-1.15, -0.39) | <0.001 | 87.8 | Low |  |
| **Comparator Type** | Active control | 11 | 407 | -0.63 (-1.03, -0.24) | 0.002 | 86.2 | Low | **0.272** |
|  | No intervention | 15 | 679 | -1.09 (-1.57, -0.60) | <0.001 | 92.7 | Low |  |
|  | Usual care | 6 | 281 | -0.77 (-1.29, -0.26) | 0.003 | 87.6 | Low |  |
|  | Sham control | 2 | 97 | -0.16 (-1.04, 0.72) | 0.714 | 89.2 | Very low |  |
| **Population Type** | Chronic disease population | 5 | 171 | -0.58 (-1.10, -0.07) | 0.026 | 78.3 | Low | **0.381** |
|  | Healthy population | 11 | 512 | -0.84 (-1.42, -0.27) | 0.004 | 92.3 | Low |  |
|  | Psychological disorder population | 9 | 374 | -1.17 (-1.71, -0.63) | <0.001 | 91.4 | Low |  |
|  | Cancer population | 9 | 407 | -0.63 (-1.08, -0.18) | 0.006 | 89.9 | Low |  |
| **Outcome Scale** | Self-reported | 28 | 1245 | -0.89 (-1.19, -0.59) | <0.001 | 90.6 | Low | **0.288** |
|  | Clinician-rated | 6 | 219 | -0.52 (-1.13, 0.09) | 0.094 | 85.2 | Very low |  |

# Fig S10. Bayesian dose–response model diagnostics and posterior distribution of the exploratory low-point dose


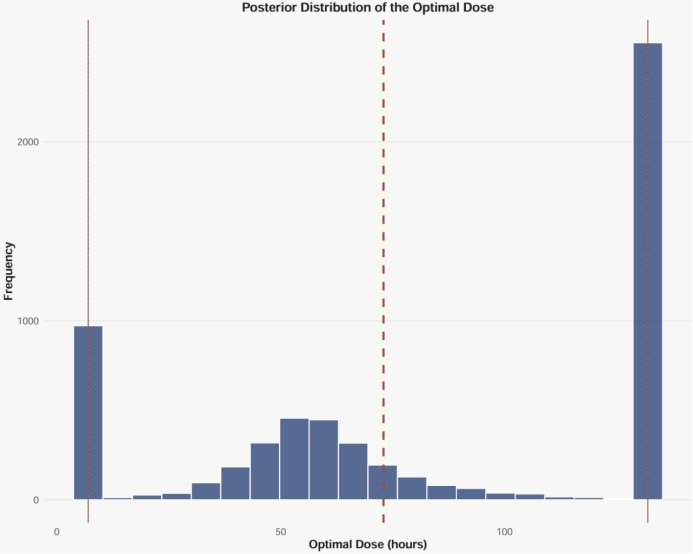


## Fig S10A. Posterior distribution of the exploratory low-point dose

Note: Posterior distribution of the exploratory low-point cumulative intervention dose. The distribution suggests a possible favorable range near the upper observed dose boundary, but the posterior uncertainty remains substantial.


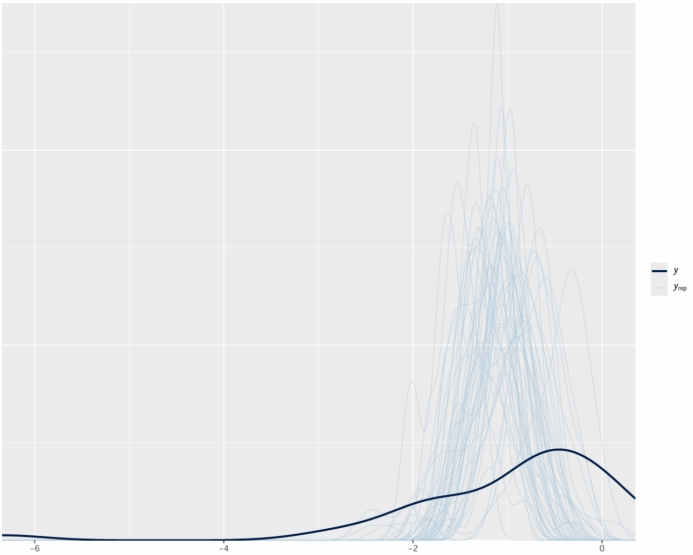


## Fig S10B. Posterior predictive density check

Note: Posterior predictive density check. The model generally captured the central tendency of the observed effect-size distribution, although some discrepancies remained in the distributional tails.


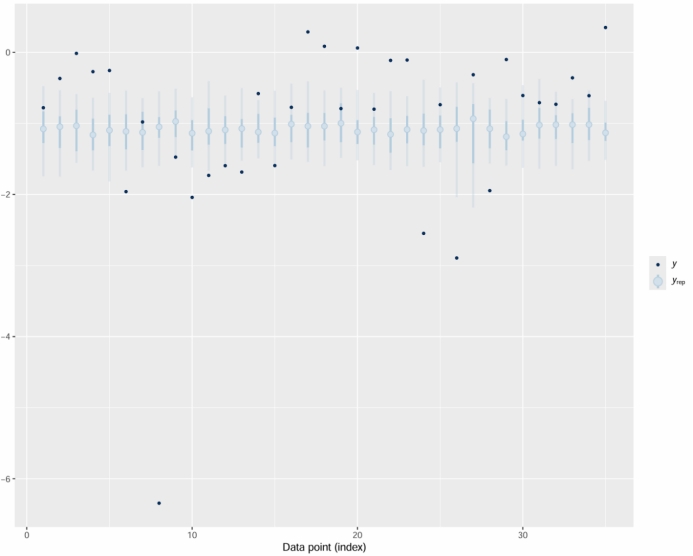


## Fig S10C. Posterior predictive interval check

Note: Posterior predictive interval check across individual data points. Most observed values were covered by the posterior predictive intervals, whereas several extreme negative effects showed poorer model fit.


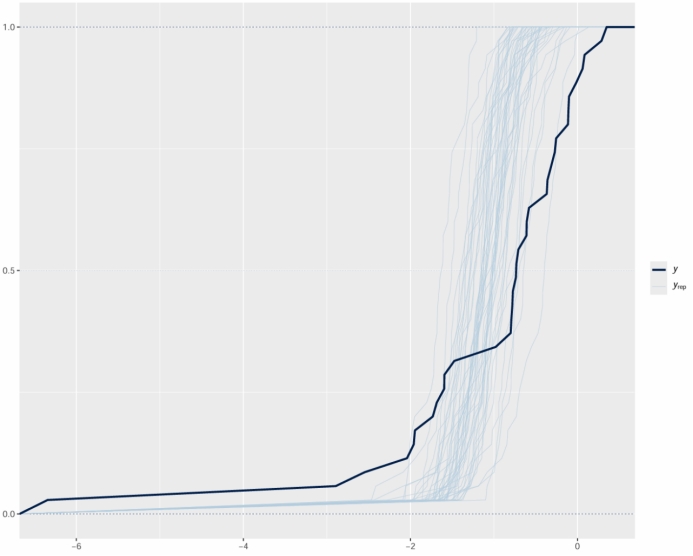


## Fig S10D. Posterior predictive ECDF check

Note: Posterior predictive empirical cumulative distribution function check. The model broadly reproduced the observed cumulative distribution, but uncertainty remained due to the limited number of studies and sparse dose distribution.

# Fig S11. Performance diagnostics of the XGBoost prediction model


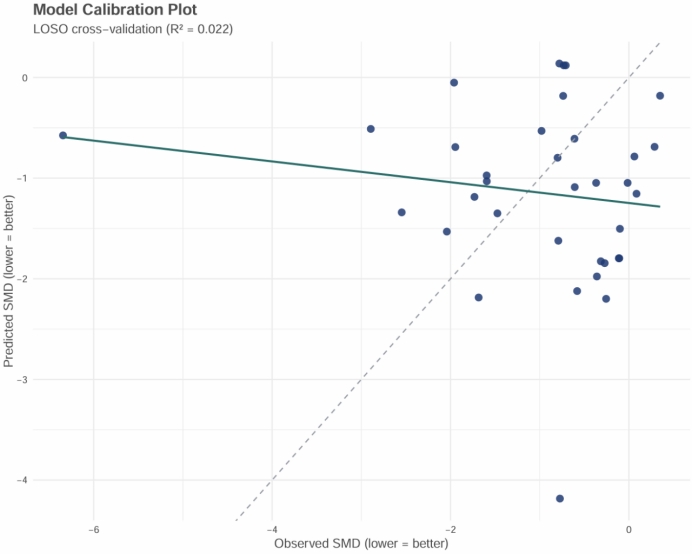


## Fig S11A. Performance diagnostics of the XGBoost prediction model

Notes: Model calibration plot based on leave-one-study-out cross-validation. The low R² value indicates limited predictive accuracy and supports the exploratory nature of the machine-learning analysis.


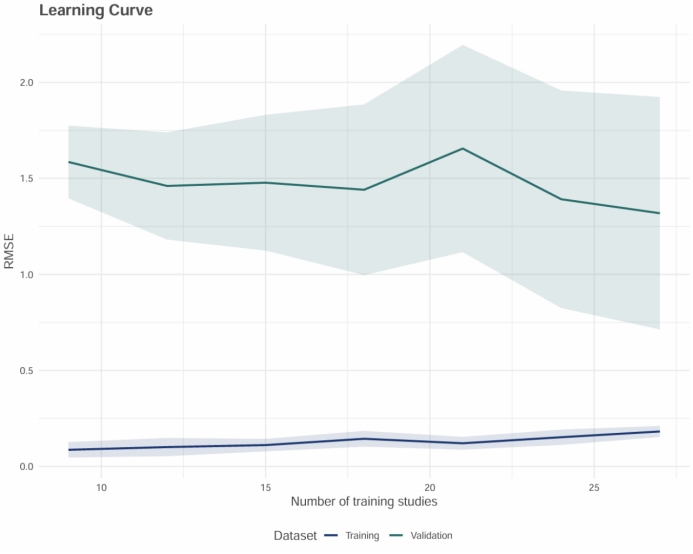


## Fig S11B. Learning Curve

Notes: Learning curve of the XGBoost model. The validation error remained substantially higher than the training error, suggesting limited generalizability and potential overfitting due to the small number of studies.

# Fig S12. SHAP dependence plots for continuous predictors in the XGBoost model


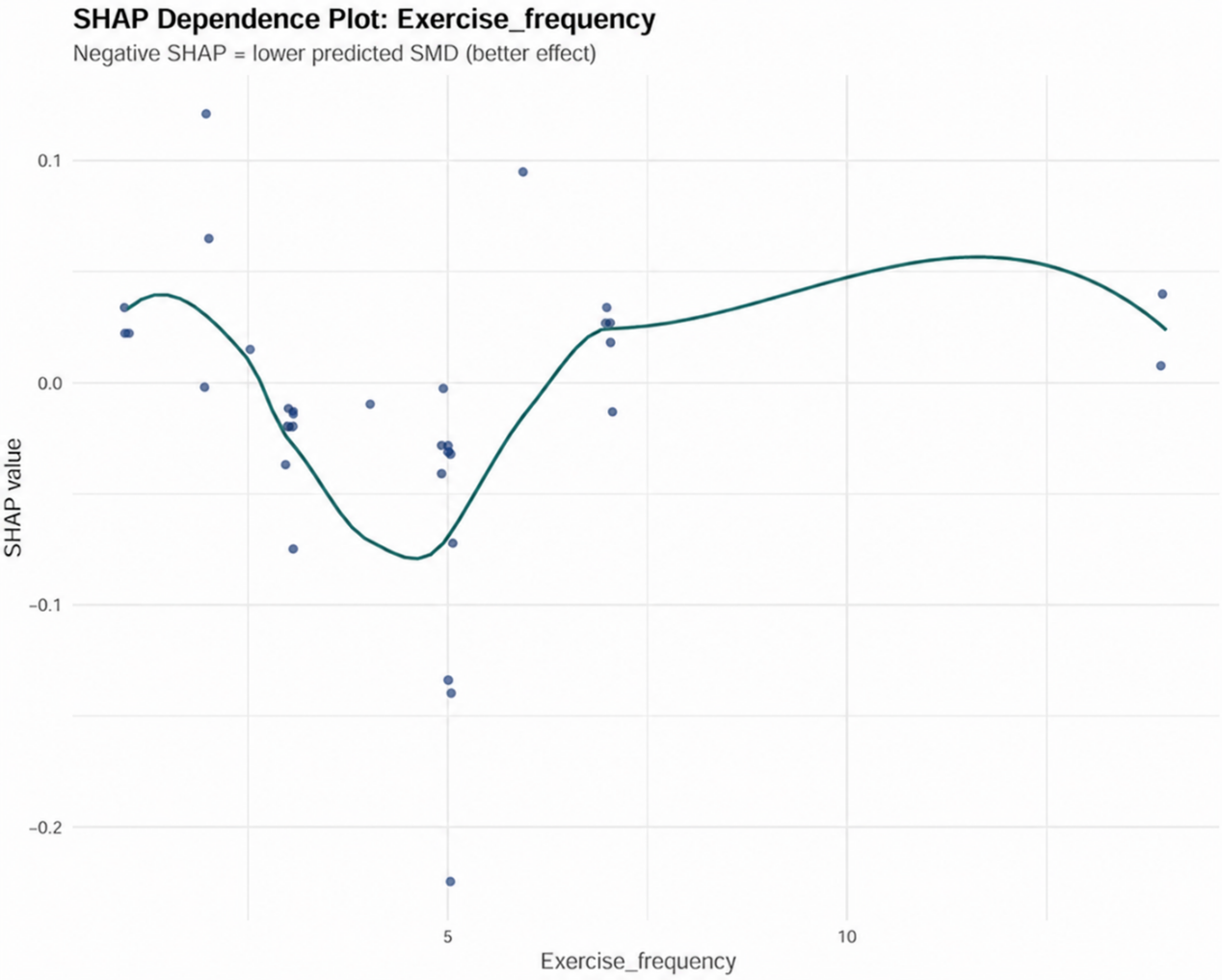


## Fig S12A. Exercise frequency

Notes: Exercise frequency showed a nonlinear and unstable association with predicted effects, suggesting that higher frequency alone may not consistently predict stronger antidepressant effects.


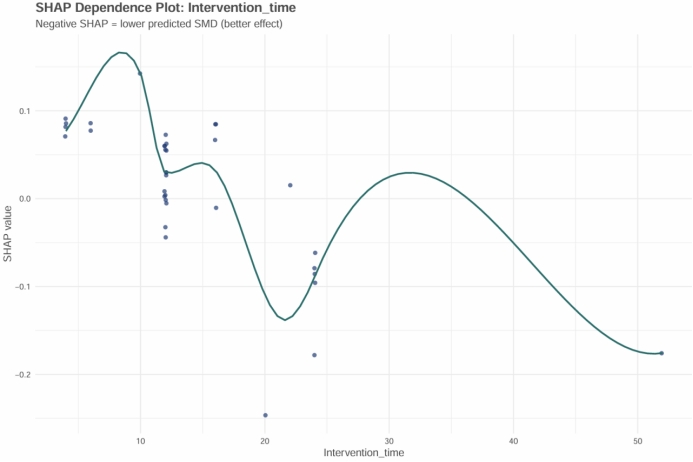


## Fig S12B. Intervention duration

Notes: Intervention duration showed a nonlinear pattern, with lower SHAP values observed around intermediate-to-longer durations. However, the sparse distribution of studies limits causal interpretation.


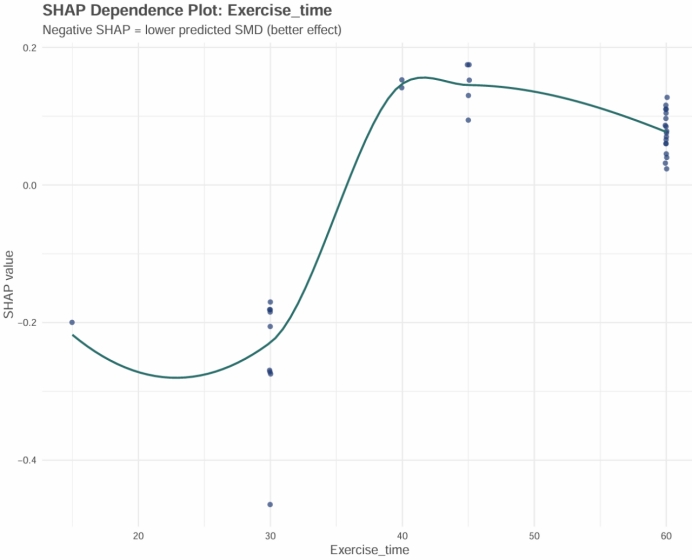


## Fig S12C. Exercise time

Notes: Exercise time showed lower SHAP values at shorter session lengths, suggesting a possible stronger predicted effect for shorter sessions; however, this pattern may be confounded by intervention context and study characteristics.


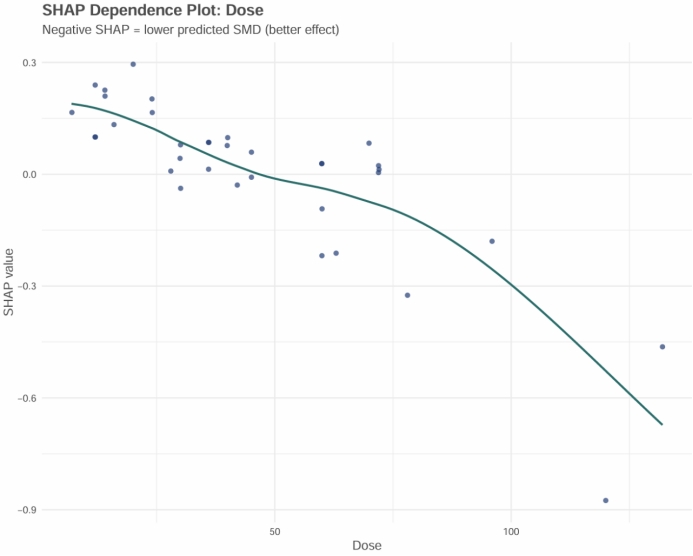


## Fig S12D. Total intervention dose

Notes: Total intervention dose showed a decreasing SHAP trend, suggesting that higher cumulative doses may be associated with stronger predicted effects. This finding should be interpreted with caution because high-dose observations were sparse and near the upper boundary of the observed data.


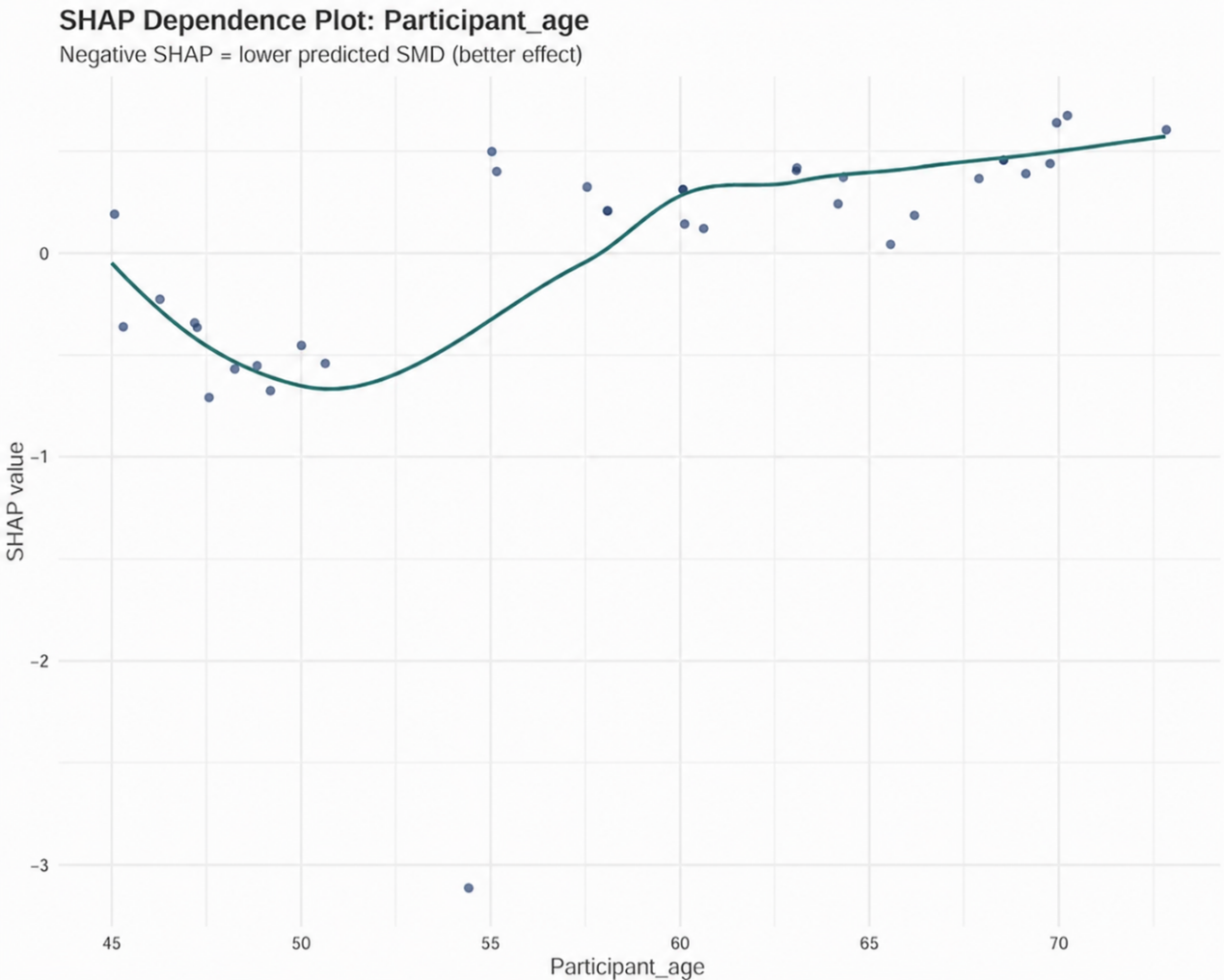


## Fig S12E. Participant age

Notes: Participant age showed a generally increasing SHAP trend, indicating weaker predicted effects with older age, broadly consistent with the meta-regression finding.

# Fig S13. SHAP dependence plots for categorical study-level predictors in the XGBoost model


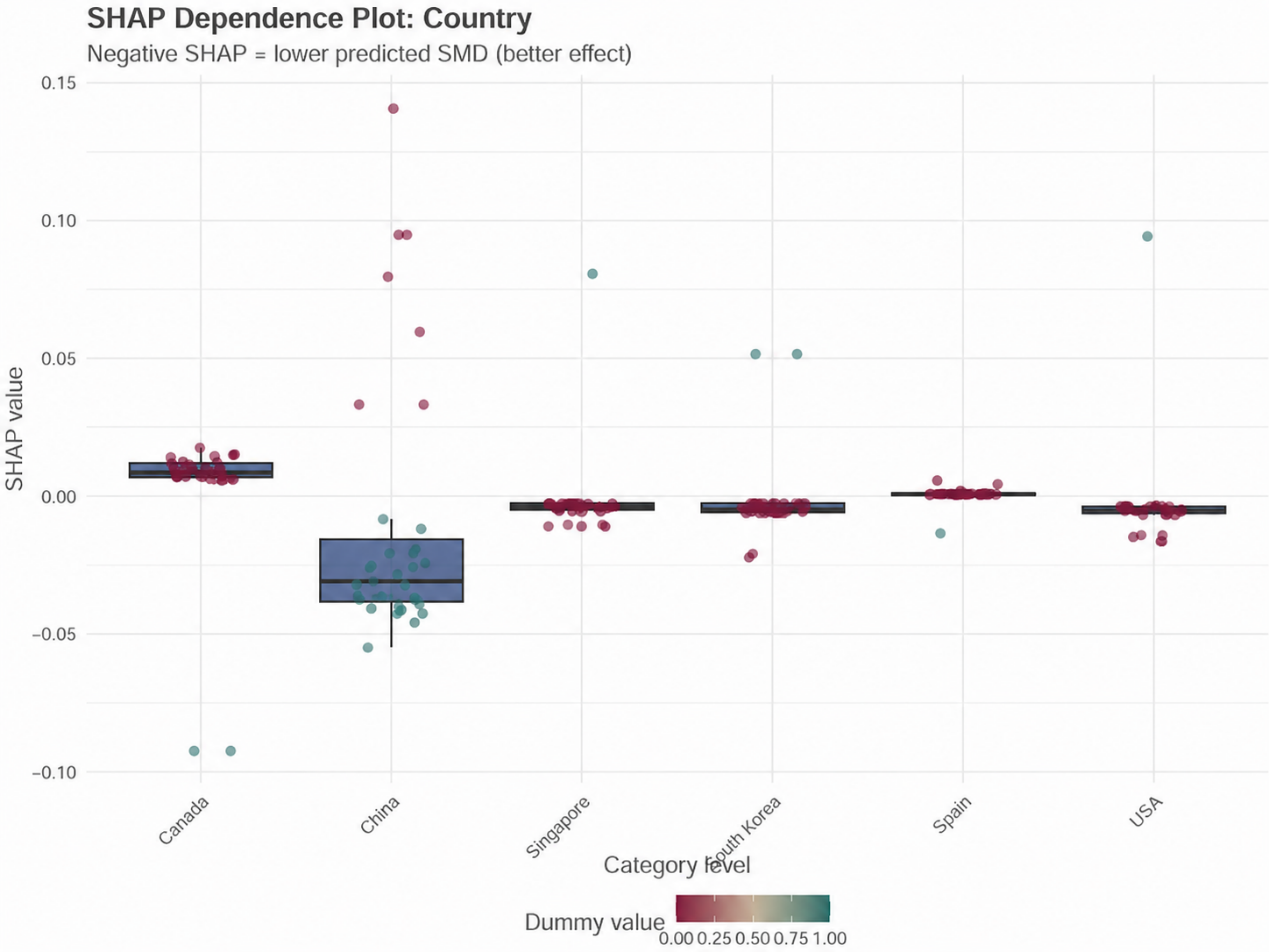


## Fig S13A. Country

Notes: Country or region showed heterogeneous SHAP distributions, but the limited number of non-Chinese studies constrains interpretation.


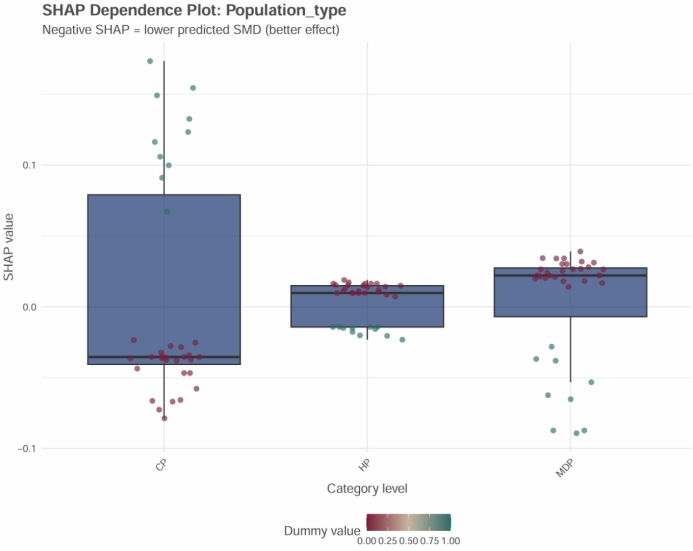


## Fig S13B. Population type

Notes: Population type showed variable SHAP distributions across categories, suggesting potential population-related heterogeneity in predicted intervention effects.


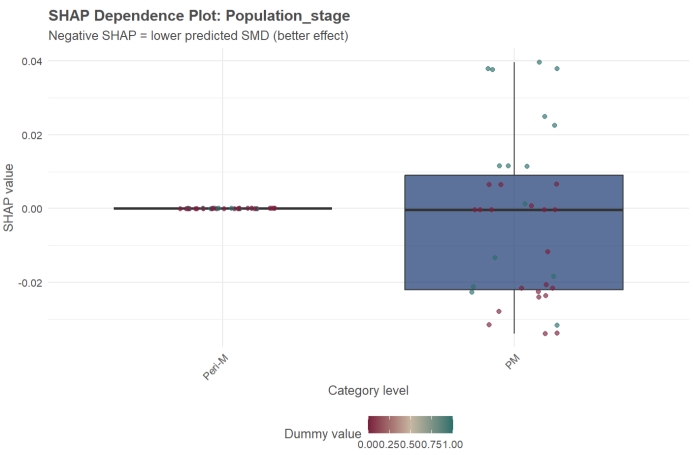


## Fig S13C. Population stage

Notes: The population stage showed limited and unstable SHAP variation, suggesting that sex composition was not a robust predictor in the current dataset.


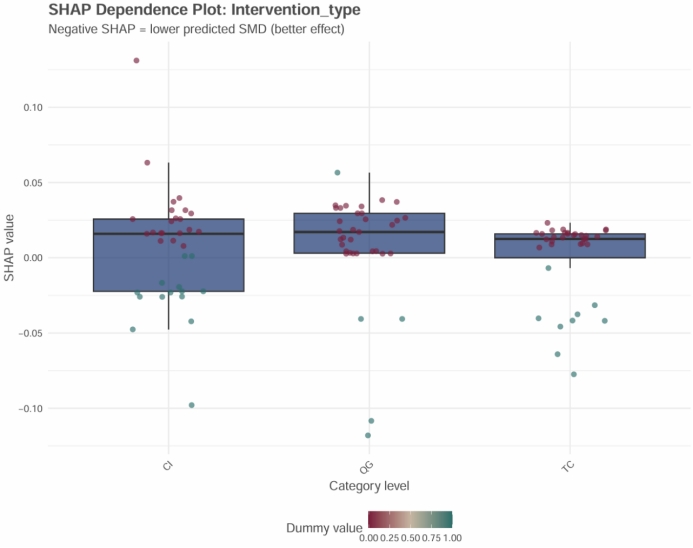


## Fig S13D. Intervention type

Notes: Intervention type showed overlapping SHAP distributions across modalities, indicating no clear modality-specific dominance in predicted effects.


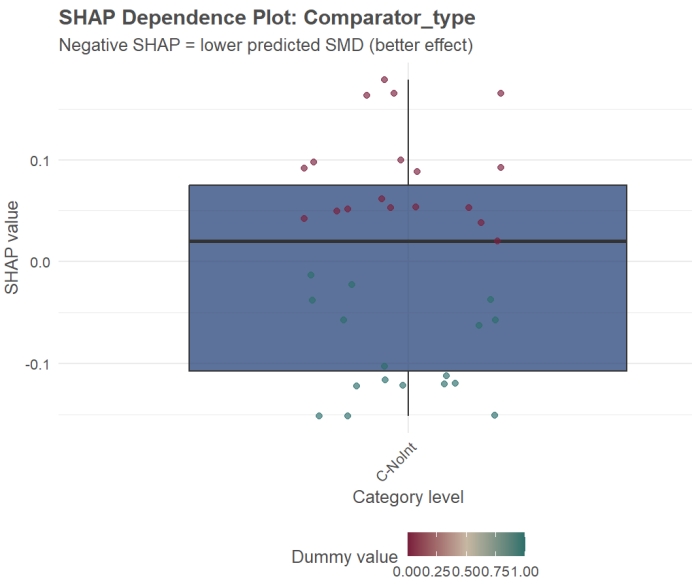


## Fig S13E. Comparator type

Notes: Comparator type showed wide SHAP variation, indicating that control condition may influence predicted effects, although the dummy-coded representation limits direct comparison across all comparator categories.


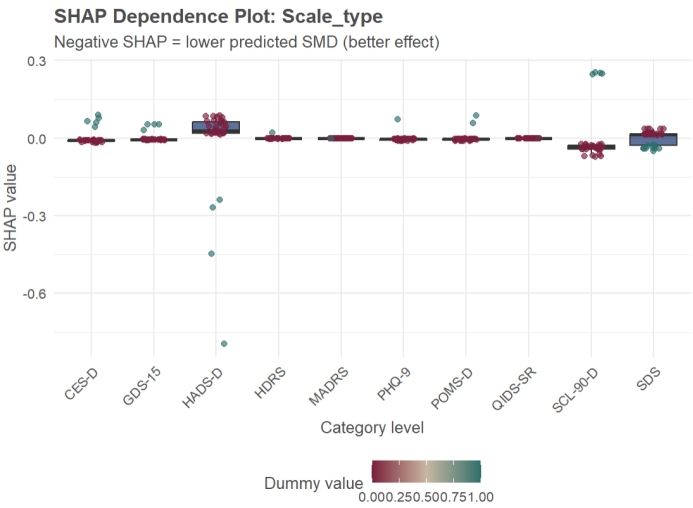


## Fig S13F. Scale type

Notes: Outcome scale type showed notable SHAP variability across depression instruments, suggesting that measurement characteristics may contribute to heterogeneity in predicted effects.

# PRISMA_2020_checklist

| **Section and Topic** | **Item #** | **Checklist item** | **Location where item is reported** |
| --- | --- | --- | --- |
| **TITLE:** Effects of Traditional Chinese Mind–Body Exercises on Depression in Middle-Aged and Older Adults: A Multilevel Dose–Response and Machine Learning Meta-Analysis | | |  |
| Title | 1 | Identify the report as a systematic review. | 1 |
| **ABSTRACT** | | |  |
| Abstract | 2 | See the PRISMA 2020 for Abstracts checklist. | 1-2 |
| **INTRODUCTION** | | |  |
| Rationale | 3 | Describe the rationale for the review in the context of existing knowledge. | 2-3 |
| Objectives | 4 | Provide an explicit statement of the objective(s) or question(s) the review addresses. | 3 |
| **METHODS** | | |  |
| Eligibility criteria | 5 | Specify the inclusion and exclusion criteria for the review and how studies were grouped for the syntheses. | 3-4 |
| Information sources | 6 | Specify all databases, registers, websites, organisations, reference lists and other sources searched or consulted to identify studies. Specify the date when each source was last searched or consulted. | 4 |
| Search strategy | 7 | Present the full search strategies for all databases, registers and websites, including any filters and limits used. | 4 |
| Selection process | 8 | Specify the methods used to decide whether a study met the inclusion criteria of the review, including how many reviewers screened each record and each report retrieved, whether they worked independently, and if applicable, details of automation tools used in the process. | 4-5, 7 |
| Data collection process | 9 | Specify the methods used to collect data from reports, including how many reviewers collected data from each report, whether they worked independently, any processes for obtaining or confirming data from study investigators, and if applicable, details of automation tools used in the process. | 5 |
| Data items | 10a | List and define all outcomes for which data were sought. Specify whether all results that were compatible with each outcome domain in each study were sought (e.g. for all measures, time points, analyses), and if not, the methods used to decide which results to collect. | 4-5 |
|  | 10b | List and define all other variables for which data were sought (e.g. participant and intervention characteristics, funding sources). Describe any assumptions made about any missing or unclear information. | 10-14 |
| Study risk of bias assessment | 11 | Specify the methods used to assess risk of bias in the included studies, including details of the tool(s) used, how many reviewers assessed each study and whether they worked independently, and if applicable, details of automation tools used in the process. | 6 |
| Effect measures | 12 | Specify for each outcome the effect measure(s) (e.g. risk ratio, mean difference) used in the synthesis or presentation of results. | 5 |
| Synthesis methods | 13a | Describe the processes used to decide which studies were eligible for each synthesis (e.g. tabulating the study intervention characteristics and comparing against the planned groups for each synthesis (item #5)). | 3-6 |
|  | 13b | Describe any methods required to prepare the data for presentation or synthesis, such as handling of missing summary statistics, or data conversions. | 5-6 |
|  | 13c | Describe any methods used to tabulate or visually display results of individual studies and syntheses. | 5-6, 10-24 |
|  | 13d | Describe any methods used to synthesize results and provide a rationale for the choice(s). If meta-analysis was performed, describe the model(s), method(s) to identify the presence and extent of statistical heterogeneity, and software package(s) used. | 5-6 |
|  | 13e | Describe any methods used to explore possible causes of heterogeneity among study results (e.g. subgroup analysis, meta-regression). | 5-6 |
|  | 13f | Describe any sensitivity analyses conducted to assess robustness of the synthesized results. | 5-6 |
| Reporting bias assessment | 14 | Describe any methods used to assess risk of bias due to missing results in a synthesis (arising from reporting biases). | 5-6 |
| Certainty assessment | 15 | Describe any methods used to assess certainty (or confidence) in the body of evidence for an outcome. | 6 |
| **RESULTS** | | |  |
| Study selection | 16a | Describe the results of the search and selection process, from the number of records identified in the search to the number of studies included in the review, ideally using a flow diagram. | 7 |
|  | 16b | Cite studies that might appear to meet the inclusion criteria, but which were excluded, and explain why they were excluded. | 7 |
| Study characteristics | 17 | Cite each included study and present its characteristics. | 10-14 |
| Risk of bias in studies | 18 | Present assessments of risk of bias for each included study. | 8-9 |
| Results of individual studies | 19 | For all outcomes, present, for each study: (a) summary statistics for each group (where appropriate) and (b) an effect estimate and its precision (e.g. confidence/credible interval), ideally using structured tables or plots. | 10-16 |
| Results of syntheses | 20a | For each synthesis, briefly summarise the characteristics and risk of bias among contributing studies. | 8-14 |
|  | 20b | Present results of all statistical syntheses conducted. If meta-analysis was done, present for each the summary estimate and its precision (e.g. confidence/credible interval) and measures of statistical heterogeneity. If comparing groups, describe the direction of the effect. | 14-24 |
|  | 20c | Present results of all investigations of possible causes of heterogeneity among study results. | 17-23 |
|  | 20d | Present results of all sensitivity analyses conducted to assess the robustness of the synthesized results. | 15-17 |
| Reporting biases | 21 | Present assessments of risk of bias due to missing results (arising from reporting biases) for each synthesis assessed. | 15-17 |
| Certainty of evidence | 22 | Present assessments of certainty (or confidence) in the body of evidence for each outcome assessed. | 15 |
| **DISCUSSION** | | |  |
| Discussion | 23a | Provide a general interpretation of the results in the context of other evidence. | 24-25 |
|  | 23b | Discuss any limitations of the evidence included in the review. | 26-27 |
|  | 23c | Discuss any limitations of the review processes used. | 26-27 |
|  | 23d | Discuss implications of the results for practice, policy, and future research. | 26-27 |
| **OTHER INFORMATION** | | |  |
| Registration and protocol | 24a | Provide registration information for the review, including register name and registration number, or state that the review was not registered. | 3 |
|  | 24b | Indicate where the review protocol can be accessed, or state that a protocol was not prepared. | 3 |
|  | 24c | Describe and explain any amendments to information provided at registration or in the protocol. | N/A |
| Support | 25 | Describe sources of financial or non-financial support for the review, and the role of the funders or sponsors in the review. | 27 |
| Competing interests | 26 | Declare any competing interests of review authors. | 27 |
| Availability of data, code and other materials | 27 | Report which of the following are publicly available and where they can be found: template data collection forms; data extracted from included studies; data used for all analyses; analytic code; any other materials used in the review. | 27 |

# Reference

1. Song J, Wei L, Cheng K, Lin Q, Xia P, Wang X, Wang X, Yang T, Chen B, Ding A. The effect of modified tai chi exercises on the physical function and quality of life in elderly women with knee osteoarthritis. *Front Aging Neurosci* (2022) 14:860762. doi: 10.3389/fnagi.2022.860762

2. Dong L, Lee J-B, Kim Y-K, Kim Y-S. The effects of health qigong training of elderly single women on pain consciousness and depression. *Int J Appl Sports Sci* (2013) 25:

3. Liao J. Effects of 24-week tai chi exercise on mental health of urban elderly women. *Chin J Gerontol* (2015) 35:7232–7233.

4. Ma Z, Wang B, Xi B. Effects of health qigong mawangdui daoyinshu exercise on mood state and anxiety level in middle-aged and elderly women. *Chin J Gerontol* (2016) 36:3248–3249. doi: 10.3969/j.issn.1005-9202.2016.13.075

5. Mo G, Wang B. Effects of health qigong taiji yangsheng staff exercise on mood state and mental health in elderly women. *Chin J Gerontol* (2016) 36:5401–5403. doi: 10.3969/j.issn.1005-9202.2016.21.086

6. Xu Z, Li Y, Mei X, Li Y, Meng X. Application of ORTCC management combined with baduanjin in postoperative rehabilitation of breast cancer patients. *Beijing J Tradit Chin Med* (2025) 44:697–701. doi: 10.16025/j.1674-1307.2025.06.005

7. Yang X, Wang Y. Effect of baduanjin on pulmonary function and mental state in perimenopausal women with chronic obstructive pulmonary disease. *J Women Child Health* (2023) 2:61–63.

8. Ma S, Dou N, Chen C, Zhao Y, Li S. Effect of the traditional baduanjin exercise in women with peri-menopausal syndrome and depression. *Chin Gen Pract* (2010) 13:2864–2865.

9. Shan B, Tang H, Wang S. Effect of Ba duan jin combined with du mai fumigation on improving sleep quality and negative emotions in perimenopausal insomnia patients. *Prog Mod Biomed* (2025) 25:2351–2356. doi: 10.13241/j.cnki.pmb.2025.14.014

10. Luo Y, Chen S, Shao L, Xie J, Zhu A, Zhu C, Pan T. Effects of baduanjin combined with wuxing music on anxiety and depression in patients with breast cancer chemotherapy. *Chin Community Dr* (2021) 37:181–181. doi: 10.3969/j.issn.1007-614x.2021.19.085

11. Ma S, Chen C, Zhao Y, Guo Q, Li C. Effect of baduanjin on quality of life in perimenopausal women with functional constipation in the community. *Chin J Gerontol* (2011) 31:926–928.

12. Wen C, Jiang W, Li R, Zhang X, Zhou T. Study on the application effect of early rehabilitation training based on the concept of enhanced recovery after surgery combined with the first four types of baduanjin in patients after modified radical mastectomy for breast cancer. *Prog Mod Biomed* (2024) 24:264–267, 279. doi: 10.13241/j.cnki.pmb.2024.02.011

13. Wei X, Yuan R, Yang J, Zheng W, Jin Y, Wang M, Jiang J, Wu C, Li K. Effects of baduanjin exercise on cognitive function and cancer-related symptoms in women with breast cancer receiving chemotherapy: a randomized controlled trial. *Support Care Cancer* (2022) 30:6079–6091. doi: 10.1007/s00520-022-07015-4

14. Larkey LK, Roe DJ, Weihs KL, Jahnke R, Lopez AM, Rogers CE, Oh B, Guillen-Rodriguez J. Randomized controlled trial of qigong/tai chi easy on cancer-related fatigue in breast cancer survivors. *Ann Behav Med* (2015) 49:165–176. doi: 10.1007/s12160-014-9645-4

15. Chen Z, Meng Z, Milbury K, Bei W, Zhang Y, Thornton B, Liao Z, Wei Q, Chen J, Guo X. Qigong improves quality of life in women undergoing radiotherapy for breast cancer: results of a randomized controlled trial. *Cancer* (2013) 119:1691–1698. doi: 10.1002/cncr.27904

16. Sun J, Miao W, Kang C, Yang C, Li X, Gao J, Guo J. Effect of baduanjin combined with resting and meditation on negative emotion and immune function of breast cancer patients. *Tradit Chin Med Chin Mater Med* (2019) 4:131–132. doi: 10.19347/j.cnki.2096-1413.201928053

17. Shi T, Zhou M, He Y, Lin T, Zhu X, Lu J. Intervention effect of multi-track psychological support combined with traditional chinese medicine baduanjin on patients with breast cancer. *Hebei J Tradit Chin Med* (2025) 47:567–571. doi: 10.3969/j.issn.1002-2619.2025.04.009

18. Ni T, Sun L, Gao L, Wang N, Li M, Fu J, Xue J. Effect of honghuang decoction combined with baduanjin on negative emotions, fatigue degree, and quality of life in elderly patients with breast tumors undergoing chemotherapy. *J Clin Pathol Res* (2021) 41:2012–2017. doi: 10.3978/j.issn.2095-6959.2021.09.007

19. Li K, Yu H, Lin X, Su Y, Gao L, Song M, Fan H, Krokosz D, Yang H, Lipowski M. The effects of ER xian decoction combined with baduanjin exercise on bone mineral density, lower limb balance function, and mental health in women with postmenopausal osteoporosis: a randomized controlled trial. *Evidence‐Based Complement Altern Med* (2022) 2022:8602753.

20. Liu J, Si J, Zhao W. Investigation of the effect of tai chi training on depressive symptoms in perimenopausal women on the basis of serum kynurenine metabolites. *Exp Aging Res* (2025) 51:331–349. doi: 10.1080/0361073X.2024.2377427

21. Carcelén-Fraile M del C, Hita-Contreras F, Martínez-Amat A, Loureiro VB, Loureiro NEM de, Jiménez-García JD, Fábrega-Cuadros R, Aibar-Almazán A. Impact of qigong exercises on the severity of the menopausal symptoms and health-related quality of life: a randomised controlled trial. *Eur J Sport Sci* (2023) 23:656–664. doi: 10.1080/17461391.2022.2044915

22. Chang S, Cheng L, Liu H. Effects of three-duration tai-chi exercises on depression and sleep quality in older women. *Eur Geriatr Med* (2024) 15:1141–1148. doi: 10.1007/s41999-024-00981-4

23. Chen W. Effects of baduanjin on mental health of urban community-dwelling older adults. *Chin J Gerontol* (2013) 33:3472–3473. doi: 10.3969/j.issn.1005-9202.2013.14.106

24. Zhao G, Cheng R, Jie C, Sun Q, Chen R, Wu B, Lu M. Effect of shadowboxing on mild depression among middle-aged and senior people. *Chin J Conval Med* (2015) 24:452–454. doi: 10.13517/j.cnki.ccm.2015.05.002

25. Siu PM, Danny JY, Angus PY, Recchia F, Li SX, Chan RN, Fong DY, Chan DK, Hui SS, Chung KF. Tai chi or cognitive behavioural therapy for treating insomnia in middle aged and older adults: randomised non-inferiority trial. *bmj* (2025) 391: doi: 10.1136/bmj-2025-084320

26. Wen L, Chen X, Cui Y, Zhang M, Bai X. Effects of baduanjin exercise in nasopharyngeal carcinoma patients after chemoradiotherapy: a randomized controlled trial. *Support Care Cancer* (2023) 31:79. doi: 10.1007/s00520-022-07548-8

27. Park H, Rigas C, Ibrahim M, Su C-L, Eyler L, Thomas Z, Nassim M, Beaulieu S, Buck G, Rej S. Efficacy of virtually-delivered qigong/tai chi for depression in middle-and older-age adults with bipolar disorder (QT-BD): a pilot randomized controlled trial during the COVID-19 pandemic. *J Affect Disord Rep* (2023) 13:100604. doi: 10.1016/j.jadr.2023.100604

28. Ma C, Zhou W, Tang Q, Huang S. The impact of group-based tai chi on health-status outcomes among community-dwelling older adults with hypertension. *Heart Lung* (2018) 47:337–344. doi: 10.1016/j.hrtlng.2018.04.007

29. Liu Z, Zhang L, Bai L, Guo Z, Gao J, Lin Y, Zhou Y, Lai J, Tao J, Chen L. Repetitive transcranial magnetic stimulation and tai chi chuan for older adults with sleep disorders and mild cognitive impairment: a randomized clinical trial. *JAMA Netw Open* (2025) 8:e2454307. doi: 10.1001/jamanetworkopen.2024.54307

30. Tou NX, Goh SF, Harding S, Tsao MA, Ng TP, Wee S-L. Effectiveness of community-based baduanjin exercise intervention for older adults with varying frailty status: a randomized controlled trial. *Eur Rev Aging Phys Act* (2024) 21:28. doi: 10.1186/s11556-024-00363-6
